# *Gaultheria leucocarpa* var. *yunnanensis* for Treating Rheumatoid Arthritis—An Assessment Combining Machine Learning–Guided ADME Properties Prediction, Network Pharmacology, and Pharmacological Assessment

**DOI:** 10.3389/fphar.2021.704040

**Published:** 2021-10-04

**Authors:** Xiuhuan Wang, Youyi Sun, Ling Ling, Xueyang Ren, Xiaoyun Liu, Yu Wang, Ying Dong, Jiamu Ma, Ruolan Song, Axiang Yu, Jing Wei, Qiqi Fan, Miaoxian Guo, Tiantian Zhao, Rina Dao, Gaimei She

**Affiliations:** ^1^ School of Chinese Materia Medica, Beijing University of Chinese Medicine, Beijing, China; ^2^ Beijing Key Laboratory for Quality Evaluation of Chinese Materia Medica, Beijing, China

**Keywords:** rheumatoid arthritis, Dianbaizhu, anti–rheumatic arthritis fraction (ARF), quantitative structure–activity relationship (QSAR), ADME, network pharmacology

## Abstract

**Background:** Dianbaizhu (*Gaultheria leucocarpa* var. *yunnanensis*), a traditional Chinese/ethnic medicine (TC/EM), has been used to treat rheumatoid arthritis (RA) for a long time. The anti–rheumatic arthritis fraction (ARF) of *G*. *yunnanensis* has significant anti-inflammatory and analgesic activities and is mainly composed of methyl salicylate glycosides, flavonoids, organic acids, and others. The effective ingredients and rudimentary mechanism of ARF remedying RA have not been elucidated to date.

**Purpose:** The aim of the present study is to give an insight into the effective components and mechanisms of Dianbaizhu in ameliorating RA, based on the estimation of the absorption, distribution, metabolism, and excretion (ADME) properties, analysis of network pharmacology, and *in vivo* and *in vitro* validations*.*

**Study design and methods:** The IL-1β–induced human fibroblast-like synoviocytes of RA (HFLS-RA) model and adjuvant-induced arthritis in the rat model were adopted to assess the anti-RA effect of ARF. The components in ARF were identified by using UHPLC-LTQ-Orbitrap-MS^n^. The quantitative structure–activity relationship (QSAR) models were developed by using five machine learning algorithms, alone or in combination with genetic algorithms for predicting the ADME properties of ARF. The molecular networks and pathways presumably referring to the therapy of ARF on RA were yielded by using common databases and visible software, and the experimental validations of the key targets conducted *in vitro*.

**Results:** ARF effectively relieved RA *in vivo* and *in vitro*. The five optimized QSAR models that were developed showed robustness and predictive ability. The characterized 48 components in ARF had good biological potency. Four key signaling pathways were obtained, which were related to both cytokine signaling and cell immune response. ARF suppressed IL-1β–induced expression of EGFR, MMP 9, IL2, MAPK14, and KDR in the HFLS-RA .

**Conclusions**: ARF has good druggability and high exploitation potential. Methyl salicylate glycosides and flavonoids play essential roles in attuning RA. ARF may partially attenuate RA by regulating the expression of multi-targets in the inflammation–immune system. These provide valuable information to rationalize ARF and other TC/EMs in the treatment of RA.

**GRAPHICAL ABSTRACT:**
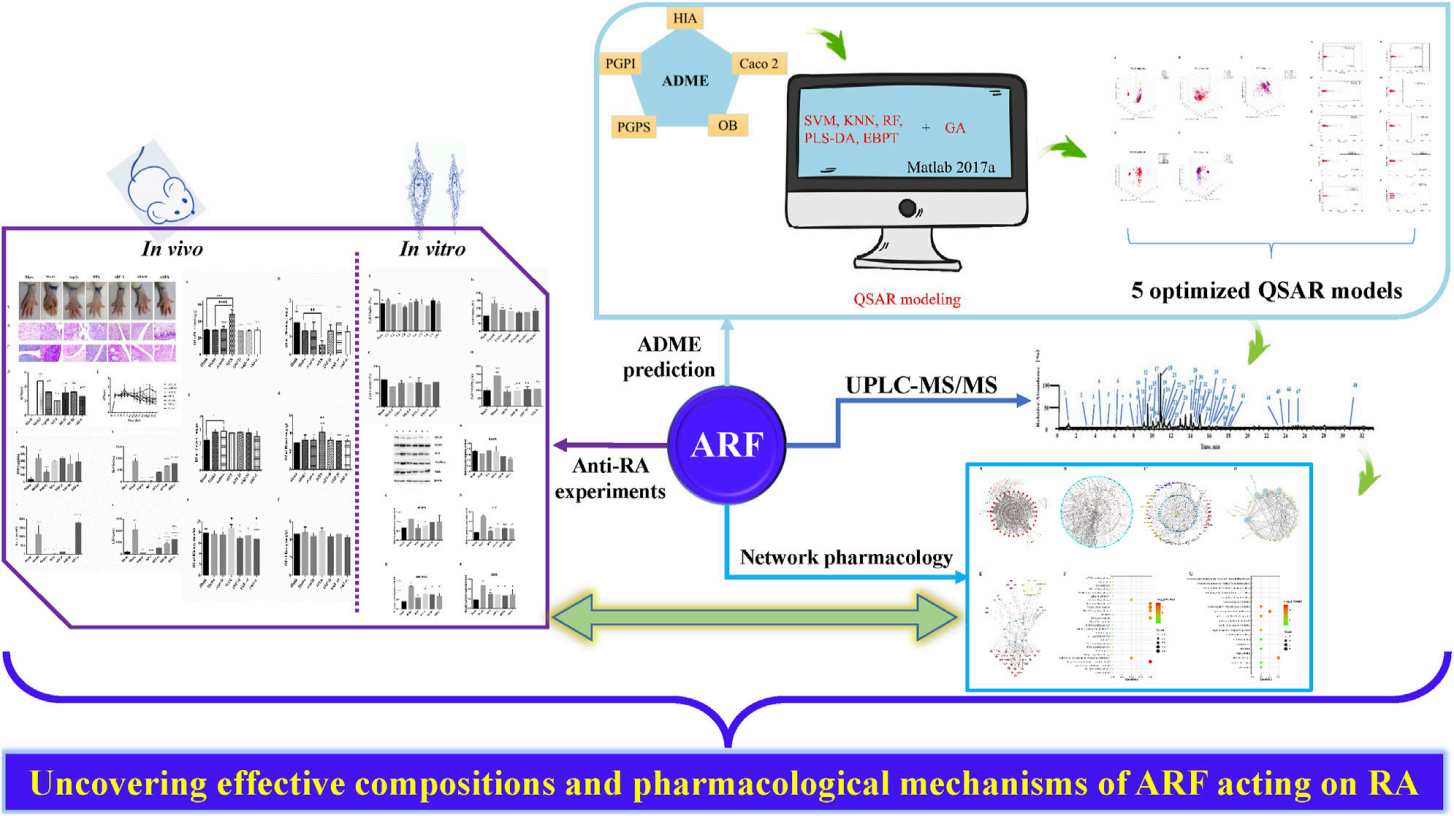
The summary framework of research ideas, the inter-relationships, and main results of this investigation. Purple, blue and light blue arrows represent the four specific implementation contents of this study design. The green arrows reflect the relationship between each section.

## Introduction

Rheumatoid arthritis (RA) is a chronic autoimmune disease with a higher prevalence in women, which according to Oliveira and Fierro (2018) is characterized by an inflammatory process, with a global prevalence ranging from 0.3 to 1%. The current drugs for RA are mainly divided into the following categories: nonsteroidal anti-inflammatory drugs, disease modifying anti–rheumatic drugs, glucocorticoids, and biological response modifiers (Zhang et al., 2015). Limitations with these treatments have been associated with side effects and dosing inconvenience and have been observed in a proportion of patients (Zhang et al., 2015). An increasing number of complementary and alternative drug therapy have been developed to alleviate the severity of RA and to bring improvement in physical conditions of patients. The development of traditional Chinese/ethnic medicines (TC/EMs) featured by multicomponent therapy provides a representative approach for the treatment of RA.

*Gaultheria leucocarpa* var. *yunnanensis* (Franch.), known as “Dianbaizhu” in TC/EMs, belongs to the Ericaceae family and is mainly distributed in Southwest China. It has been widely used as a folk medicine for the treatment of inflammatory diseases, such as RA and chronic tracheitis in the Yi nationality (Liu et al., 2013). Many of the over-the-counter TC/EMs containing Dianbaizhu are used for the treatment of RA, for example, the Touguxiang ointment, Jingulian capsule/tablet, Fenghechubi tincture, and Dianbaizhu syrup. The main constituents of these include salicylate derivatives, lignans, flavonoids, and organic acids that have been isolated and identified from *G. yunnanensis* (Liu et al., 2015; Xu et al., 2016). Isolates obtained from *Gaultheria*, such as MSTG-B, MSTG-A, gaultherin, and chlorogenic acid, have anti-inflammatory and analgesic activities, which have been all verified using *in vivo* and *in vitro* models (Zhang et al., 2011; Xie et al., 2014; Nabavi et al., 2017). The anti–rheumatic arthritis fraction (ARF) of *G*. *yunnanensis* has been ascertained by screening activity and found to exhibit better activity than the above-mentioned singular effective constituents. The research group of this study demonstrated by simulating the gastrointestinal fluid and human gut bacteria models *in vitro* that ARF is relatively stable in the gastrointestinal tract (Wang et al., 2019). The bioactive constituents and molecular mechanisms that underlie the effects of ARF against RA progression remain unclear.

The development of systems biology and bioinformatics has created an opportunity for the discovery of the mechanisms of action of Chinese herbal medicine that are used to treat RA. Network pharmacology is a suitable tool to clarify and interpret the synergistic effects and the underlying mechanisms of multicomponent and multi-target agents from a holistic perspective (Guo et al., 2015; Kibble et al., 2015). In most studies, network pharmacology considers drug-like ingredients in herb databases, while the inclusiveness and coverage ratio of these ingredients are often neglected. Thus, especially for minority drugs, such as ethnic medicine Dianbaizhu, the primary ingredients and targets predicted by network pharmacology may deviate from the truth. Therefore, it becomes necessary to establish a more comprehensive evaluation system of drug-likeness of TC/EMs.

The pharmaceutical industry at present undergoes tremendous pressure, especially in reducing healthcare costs and screening the amount of new compounds (Yang et al., 2019). The molecular properties for absorption, distribution, metabolism, and excretion (ADME) are crucial in the evaluation system for drug design and development. The development of many potential drugs has been discontinued because of their poor absorption. Several screening paradigms, including the ADME properties, have been used to enhance the probability of success through the drug development stage. Traditional research methods and models have been undergoing changes that cannot meet the requirements of rapid advances of new drug research and development, together with operation of massive data. It has become a trend to develop ADME prediction models using machine learning (ML) to process high throughput information, particularly the quantitative structure–activity relationships (QSARs), as a statistical model, which quantitatively correlates chemical structural information (described as molecular descriptors) to the response end points (biological activity, property, toxicity, etc.) (Khan and Roy, 2018). This occupies a crucial position in the establishment and prediction of the ADME data model. Currently, ADME predictions have been also applied to design new compounds against the novel coronavirus disease 2019 (COVID-19) (Hage-Melim et al., 2020). As one of the basic and hot topics in chemometrics research, this has attracted much attention and been widely used, for instance, in treatment technology for organic micropollutants (Huang et al., 2020), migration and transformation of organic pollutants, development and design of drugs, graph signal processing (Matsushita et al., 2019), and environmental-related research (Song et al., 2020). A QSAR model is developed by classification and/or regression analysis of select descriptors contributing toward targeted properties (Gaikwad et al., 2016). The related analyses implemented by ML mainly include the partial least square (PLS), random forest (RF), K-nearest neighbors (KNN), error back propagation training (EBPT), discrimination analysis (DA), PLS-DA, support vector machine (SVM), and other single classifier algorithms (Jiang et al., 2020; Maharao et al., 2020; Spiegel and Senderowitz, 2020). Some impediments in using the reported QSAR models have long existed, including variable selection, data redundancy, and a lack of consistent and homogenous data in the public domain (Lee et al., 2020). The accelerated pace of drug discovery has heightened the need for efficient prediction methods. The genetic algorithm (GA) has great advantages in variable selection, model optimization, and high efficiency. Although the application of the GA alone or in combination with other algorithms in QSAR model building is a crucial end point, little or no data exists in the public domain (Ermondi and Caron, 2019). Therefore, an attempt was made to combine the GA with several single classifiers to build a QSAR model in order to get efficient ADME prediction models with a good prediction performance.

**FIGURE 1:**
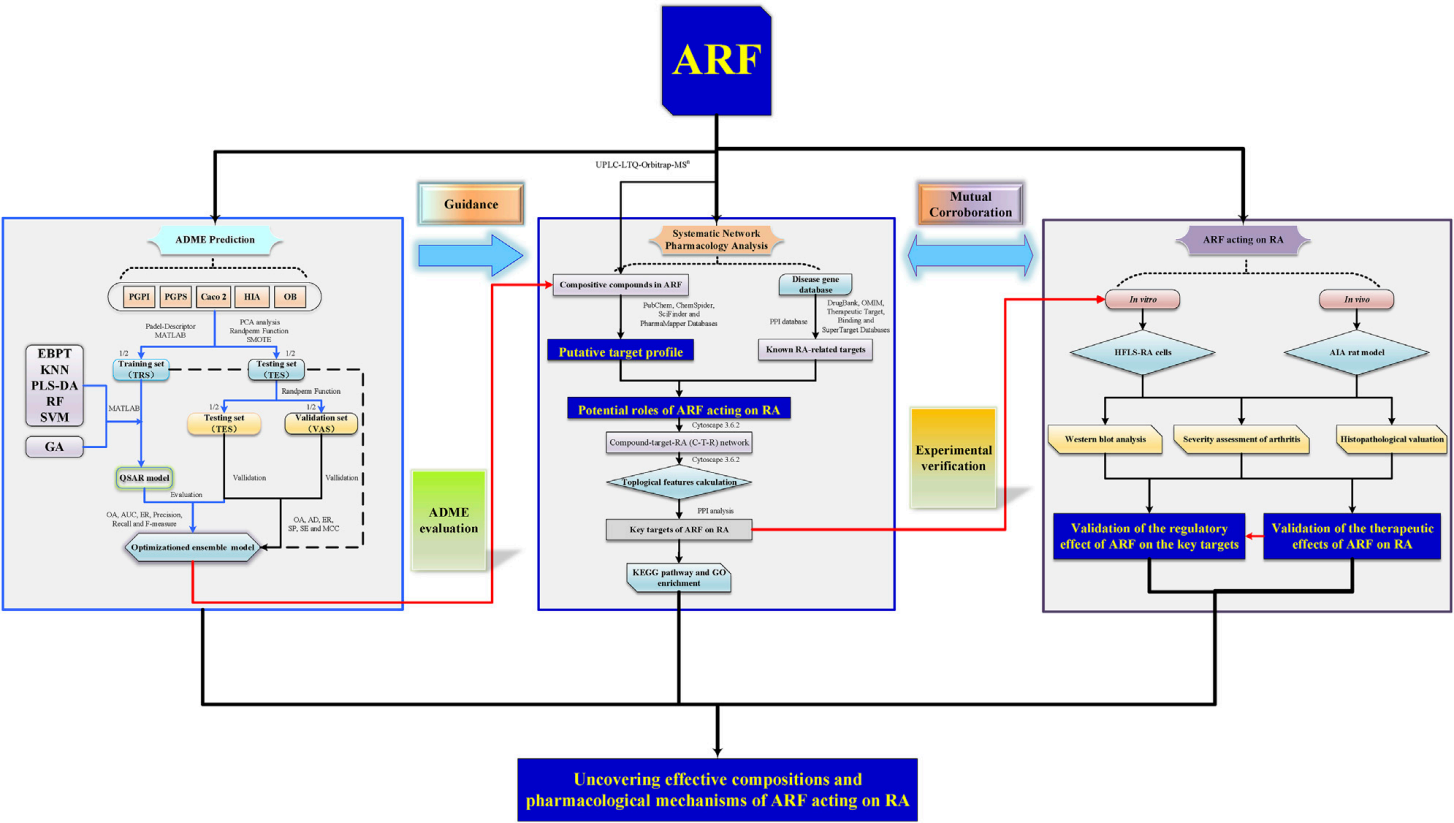
A schematic picture of the whole development strategies in unraveling the pharmacological mechanisms of ARF derived from Dianbaizhu in treating RA. For the ADME prediction section, the blue solid arrows represent the modeling process of the QSAR models, and the black solid line and dotted line arrows represent the evaluation and validation processes of the models. For the other parts, the black solid arrows stand for process transition. The red solid arrows reflect the relevance and progressiveness among the three portions.

A systematic study of the multiple components and multiscale mechanisms was carried out to investigate the remedial effect of ARF on RA, and the following steps were taken: 1) the components of ARF of *G. yunnanensis* were identified using ultra-high-performance liquid chromatography coupled with linear ion trap Orbitrap mass spectrometry (UHPLC-LTQ-Orbitrap-MS^n^); 2) the organ indexes (OIs) in adjuvant-induced arthritis (AIA) in rats were measured *in vivo*, histological and pathological changes of the joints were surveyed, and the repression capability of ARF on RA, as well as the release of inflammatory factors, was analyzed; 3) QSAR models of the ADME features based on using RF, SVM, KNN, PLS-DA, and EBPT alone or in combination with the GA were proposed. The ADME-related parameters of the compounds of ARF were predicted and evaluated; 4) protein–protein interaction (PPI) network analyses, the KEGG pathway, and gene ontology (GO) enrichment analyses were performed for key targets; 5) the compound–common target and RA-related pathway networks were developed to investigate the potential mechanisms, and the key targets were selected for subsequent experimental verification *in vitro*. Consequently, a better comprehension of the underlying pharmacodynamics of ARF could provide new insights for treatment against RA. At present, this exploration strategy could present the critical active ingredients and potential mechanisms of ARF in relieving RA and bring weighty benefits in screening new clinical therapeutic approaches on RA. The whole framework diagram of this study is depicted in Figure 1.

## Materials and Methods

### Reagents and Antibodies

Dianbaizhu was collected from Chuxiong (Yunnan, China). Methotrexate (MTX) was bought from SPH Sine Pharmaceutical Laboratories Co. Ltd. (H3102067804, Shanghai, China). Aspirin was purchased from Jiangsu Pingguang Pharmaceutical Co. Ltd. (H19980197, Jiangsu, China). Freund’s complete adjuvant (FCA, 101722747) and β–actin (A5441) were obtained from Sigma (USA). Human fibroblast-like synoviocytes of RA (HFLS-RA) were purchased from Hunan Fenghui Biotechnology Co. Ltd. (Hunan, China). The DMEM/F12 medium (18219003), FBS (190913), and penicillin streptomycin solution (30002303) were obtained from Beijing BioDee Biotechnology Co. Ltd. (Beijing, China). IL-1β (P01584) was supplied by Novoprotein Technology Co. Ltd. (Jiangsu, China). Rat IL-2 ELISA kit (KA30281115), Rat IL-1β ELISA kit (KA301B90421), Rat IL-6 ELISA kit (KA30690741) and Rat TNF-α ELISA kit (KA38290222) were bought from Beijing Biodragon Immunotechnologies Co., Ltd. (Beijing, China). The primary antibody (anti-rabbit) and secondary antibody (anti-mouse) were bought from Cell Signaling Technology, Inc. (CST, USA). Antibodies against the following proteins were used: EGFR (18986-1-AP), MMP9 (10375-2-AP), IL2 (Ab92381, Abcam), MAPK14 (8690, CST) and KDR (Ab126679, Abcam). These antibodies as well as RIPA Lysis Buffer (S1004), protease inhibitor (KGP603), BCA KIT (Thermo, USA) were provided from Beijing Bioway Biotechnology Co., Ltd. (Beijing, China). The AB-8 resin was obtained from Cangzhou Bon Adsorber Technology Co., Ltd. (Hebei, China).

### Preparation of Anti-Rheumatic Arthritis Fraction

Dianbaizhu was acquired from the dried aerial parts of *Gaultheria leucocarpa var. yunnanensis* (Franch.) TZ Hsu and RC. Fang from *Gaultheria* Kalm ex L. (Ericaceae) (Wang et al., 2019). Samples were authenticated by Professor Shengli Wei (Beijing University of Chinese Medicine). The voucher specimens and herbs were deposited in the Chinese Materia Medica Chemistry laboratory (B417). ARF was prepared as previously described (Wang et al., 2019). Briefly, the dried aerial parts of *G. yunnanensis* (17 kg) were extracted using 238-L 30% ethanol–water three times for 2 h each time by the thermal recycling extract method. The solvent was completely evaporated from the ethanol extraction solution to yield a crude extract (approximate 1.7 kg). The total ethanol extract was dissolved in water, and then adsorbed by the AB-8 resin column. It was eluted with distilled water and 35% ethanol–water in order, yielding the 35% elution fraction (approximate 500 g). This fraction was concentrated under reduced pressure to yield ARF powder. After which it was dissolved in distilled water and passed through a 0.22-μm filter for UHPLC-LTQ-Orbitrap-MS^n^ analyses.

### Identification of Compounds in Anti-Rheumatic Arthritis Fraction by Using UHPLC-LTQ-Orbitrap-MS^n^

For the UPLC-ESI-MS^n^ experiment, most of the analysis conditions were consistent with the reported research of Wang et al. (2019), except for the gradient program and injection volume. The mobile phase system was made up of 0.1% formic acid–aqueous solution (A) and acetonitrile (B). The gradient program is as follows: 0–2 min, 10% B; 2–10 min, 10–20% B; 10–12.5 min, 20% B; 12.5–17.5 min, 20–40% B; 17.5–25 min, 40–80% B; 25–27.5 min, 80–95% B; and 27.5–33 min, 95% B. The injection volume was 5 μL.

### Animal Experiments

#### Animals

The operations were performed on all the animals in accordance with the China Physiological Society's Guiding Principles in the Care and Use of Animals, as well as the authorization of the Animal Care Committee of Beijing University of Chinese Medicine.

The 8-week-old SPF Wistar rats with a mean weight of (200 ± 20) g, equal numbers of male and female rats, were obtained from Beijing Vital River Laboratory Animal Technology Co. Ltd. (certification number SCXK (Jing) 2016-0006). The animals were housed in suitable temperature and humidity conditions with a 12-h light/dark cycle at the Beijing University of Chinese Medicine. All Wistar rats were allowed to acclimatize themselves for 7 days, and had free access to tap water and food.

#### Adjuvant Induced Arthritis Model Establishment and Grouping

ARF was diluted with distilled water for tests *in vivo*. The lowest dosage selection for ARF was twice the daily dose of Dianbaizhu followed for patients with RA (25 g/60 kg body weight). After acclimation for 1 week, the Wistar rats were randomly divided into seven groups (*n* = 8): the control group (Blank), model group (Model), ARF-treated group 1 (608 mg/kg/d, ARF-H), ARF-treated group 2 (304 mg/kg/d, ARF-M), ARF-treated group 3 (152 mg/kg/d, ARF-L), MTX-treated group (0.5 mg/kg/d), and Aspirin-treated group (100 mg/kg/d). The RA model was established with subcutaneous injections of FCA in the right hind foot. After 7 days of the injection, the ARF-, MTX- and Aspirin-treated groups, respectively, were orally administered ARF, MTX, and aspirin for a period of 21 days. The Blank and Model groups were treated to distilled water.

#### Severity Assessment of Arthritis

The rats were observed once every two days after primary immunization. The severity of arthritis was evaluated as in previous studies (Guo et al., 2016; Slovák et al., 2017), including arthritis score, body weight, hind paw volume, percentage of arthritis in limbs, and the time arthritis first appeared.

#### Histopathological Analyses

Rats were sacrificed by cervical dislocation on the 28th day after the first immunization. Both hind limbs, including the paws and ankles, were dissected, fixed immediately in 4% paraformaldehyde, and embedded in paraffin. Tissue sections (5 μm) were mounted on common slides for staining with hematoxylin and eosin (H&E) or Safranin-O–Fast Green. The histopathological characteristics were evaluated blindly. The data were expressed as mean inflammation scores, and all the sections randomized and evaluated by two trained observers who were blinded to the treatment groups and the severity of arthritis of each rat (Zhang et al., 2015).

### Enzyme-Linked Immunosorbent Assay

The blood samples from all the AIA rats were obtained at day 27 and were anticoagulated with sodium heparin. The levels of IL-1β, TNF-α, IL-6, and IL-2 in the plasma were detected using commercially available rat ELISA kits according to the protocol of the kit, and absorbance was determined at 405 nm (Park et al., 2020).

### Absorption, Distribution, Metabolism, and Excretion Evaluation

The ADME properties contribute on vital task in early stages of drug discovery process. To evaluate the biological activity of ARF, KNN, SVM, RF, PLS-DA, EBPT, and GA were used to establish the QSAR prediction model for assessing the pharmacokinetic properties, namely oral bioavailability (OB), P-glycoprotein substrate (PGPS), P-glycoprotein inhibitor (PGPI), human intestinal absorption (HIA), and Caco-2 cell permeability (Caco-2).

The methods of ML were applied to construct QSAR models according to the references previous reported with some changes (Welling et al., 2015; Wenzel et al., 2019). Briefly, several QSAR models were obtained using different combinations of 1D and 2D molecular descriptors (MDs) of 1,100 compounds generated by the PaDEL-Descriptor software, and data pruning and splitting, as well as the ML tool implemented in MATLAB 2017a software (Przybyek and Cysewski, 2018; Chen et al., 2020). Before the development of the models, the list of descriptors was further refined by discarding those with a low variance and high correlation. The leave-one-out cross validation and five-fold cross validation were used for evaluation and validation purposes. The best models were selected on the basis of the accuracy of correct classifications obtained for all sets of compounds; the training set (TRS), test set (TES), and validation set (VAS) are defined in the Supplementary Material. Further validations of the prediction results were performed by comparing these with references. The detailed modeling process, methods of evaluation, validation and application domain analysis on models have been all included in the Supplementary Material.

### Construction of Networks

#### Target Prediction for Anti-Rheumatic Arthritis Fraction Ingredients

The compounds characterized by using UHPLC-LTQ-Orbitrap-MS^n^ were selected for the next protocol. The active compounds were screened according to the results of the ADME properties prediction by the QSAR models. The potential targets of these compounds were identified with a little change following the protocol published by Yang et al. (2019). The PharmMapper server (http://www.lilab-ecust.cn/pharmmapper/, Updated: 2019-04-10) was unitized to predict potential targets of ARF ingredients. In addition, the targets were retained if the fit score was ≥4.0 (if no fit score ≥4.0, the score was then adjusted to 3.5). The other parameters that were set are Generate Conformers: Yes; Maximum Generated Conformations: 300; Select Targets Set: Human Protein Targets Only; Number of Reserved Matched Targets (max 1,000): 300 (Wang et al., 2017).

#### Known Therapeutic Targets of RA

With a keyword “rheumatoid arthritis,” the known RA-related targets were obtained mainly from common existing resources, such as DrugBank and OMIM database. Only those drug–target interactions where the drugs were FDA approved for treatment of RA and those targets that were human genes/proteins were used (Tian et al., 2019). In addition, the known RA-related target data were supplemented by the targets collected from the Therapeutic Target Database, Yaozhi, ChemicalBook, BaseChem, YaoDu, SuperTarget, and Binding Database. The PPI analysis was applied to further screen the core drug targets.

#### Network Construction

The following networks were constructed using Cytoscape software (version 3.6.2): a putative target network of ARF; known RA-related targets network; common target PPI network; compound–target–pathway network of ARF (Franz et al., 2016). The PPI network was constructed on the basis of the information obtained from the STRING database (https://string-db.org/), and then visualized using the Cytoscape software (Mou et al., 2020).

#### Network Topological Feature Set Definition

To assess the topological properties of each node in the networks, four features, namely, the degree value (DV), node degree distribution (NDD), node betweenness centrality (NBC), and node closeness centrality (NCC) were evaluated according to the previous studies (Guo et al., 2016). The importance of a node in a network is determined using the values of these indices, with higher values indicating greater importance (Zheng et al., 2013).

### Enrichment Analyses

The functional annotations of the target compounds of ARF and their roles in signaling transduction were explored to analyze the GO function and KEGG pathway enrichment of the proteins involved in the PPI network by using the DAVID 6.8 database (https://david.ncifcrf.gov) (Banerjee et al., 2019). As described in previous investigations, the *p*-values obtained from the DAVID database were improved Fisher exact *p*-values (Huang et al., 2008). Statistical conspicuousness was authorized with the boundary values of *p* < 0.05.

### Experimental Validation *in vitro*

#### Cell Culture and Drug Treatment

HFLS-RA were cultured in the DMEM/F12 medium, with 10% FBS, 100 μg/ml streptomycin, and 100 U/mL penicillin under 5% CO_2_ at 37°C (Wang et al., 2020). The cells were used between passages 4 and 8 for all experiments. About 3.3252 g of ARF was ultrasonically dispersed using 50-ml DMEM/F12 medium with FBS. The suspension was diluted 10 times using the same medium to obtain an initial ARF solution (A0, 6.6504 mg/ml). A series of administration concentrations of ARF, namely 3.3252 (A1), 1.6626 (A2), 0.8313 (A3), 0.4157 (A4), 0.2078 (A5), 0.1039 (A6), 0.05196 (A7), 0.0260 (A8), 0.0130 (A9), and 0.0065 mg/ml (A10) were obtained by the two-fold dilution method. The different concentrations (A1–A10) of ARF were filtered and sterilized using a 0.22-μm filter membrane for further experiments *in vitro*. Similarly, the cytotoxicity of 5 × 10^−3^ μM (MTX-1), 1 × 10^−3^ μM (MTX-2), 5 × 10^−4^ μM (MTX-3), 5 × 10^−5^ μM (MTX-4), 5 × 10^−6^ μM (MTX-5), 1 × 10^−6^ μM (MTX-6) to HFLS-RA was also investigated. Experiments were performed at 12 h after HFLS-RA seeding. HFLS-RA (6.6 × 10^4^ cells/dish) were incubated with 1 ng/ml IL-1β and at different concentrations of ARF (1.6626, 0.4157, and 0.05196 mg/ml) or MTX (1 × 10^−5^ μM) for 48 h. To estimate cell proliferation capability, the culture media from the cells exposed to the different concentrations of ARF/MTX, with or without IL-1β, were detected by using the MTT assay (Zhai et al., 2017).

### Western Blotting

Western blotting was conducted following the methods described by Guo et al. (2017) and Zhao et al. (2018). Briefly, HFLS-RA were harvested and lysed in RIPA Lysis Buffer. The lysates were centrifuged at 16,000 × *g* for 20 min at 4°C to separate the supernatant. Firstly, the total proteins from the HFLS-RA were collected and quantified by using the BCA kit. The quantitative protein sample was subjected to sodium dodecyl sulfate–polyacrylamide gel electrophoresis, then transferred to the polyvinylidene difluoride (PVDF) membrane, and sealed with skimmed milk (5%) (Wang et al., 2020). Secondly, the PVDF membrane was incubated overnight at 4°C with the corresponding primary antibodies, then incubation with horseradish peroxidase–conjugated secondary antibody (1:20,000) (Wang et al., 2020). The membranes were then measured with a visualized chemiluminescence instrument and analyzed using the ImageJ software. β-actin was adopted as a reference.

### Statistical Analyses

Data were subjected to the Student's *t*-test or one-way analysis of variance at a significance level of *p* < 0.05 (Chen et al., 2019) using SPSS 22.0 and GraphPad Prism 6.0, and the values expressed as mean ± SD.

## Results

### Characterization of Chemical Compounds in Anti-Rheumatic Arthritis Fraction by using UHPLC-LTQ-Orbitrap-MS^n^

**FIGURE 2:**
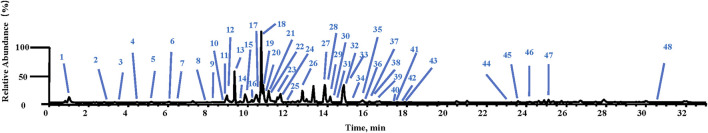
The total ion chromatogram of ARF.

In the negative ion mode, the chemical ingredients in ARF were rapidly identified by using UHPLC-LTQ-Orbitrap-MS^n^; the identification method followed for the chemical constituents in ARF has been consistent with the previously reported method by Wang et al. (2019). The mass error threshold was fixed at ± 5 ppm. Forty-eight compounds were identified and tentatively characterized, which consisted of seven methyl salicylate glycoside derivatives, 13 flavonoids, and 12 organic acids, together with 16 other compounds. The MS data of these components are listed in Supplementary Table S2, and the total ion chromatogram of ARF is shown in Figure 2.

### Anti-Rheumatic Arthritis Fraction Ameliorated Symptoms of Adjuvant Induced Arthritis Rats

#### Inference of RA-Related Pathological Processes Affected by Anti-Rheumatic Arthritis Fraction

**FIGURE 3:**
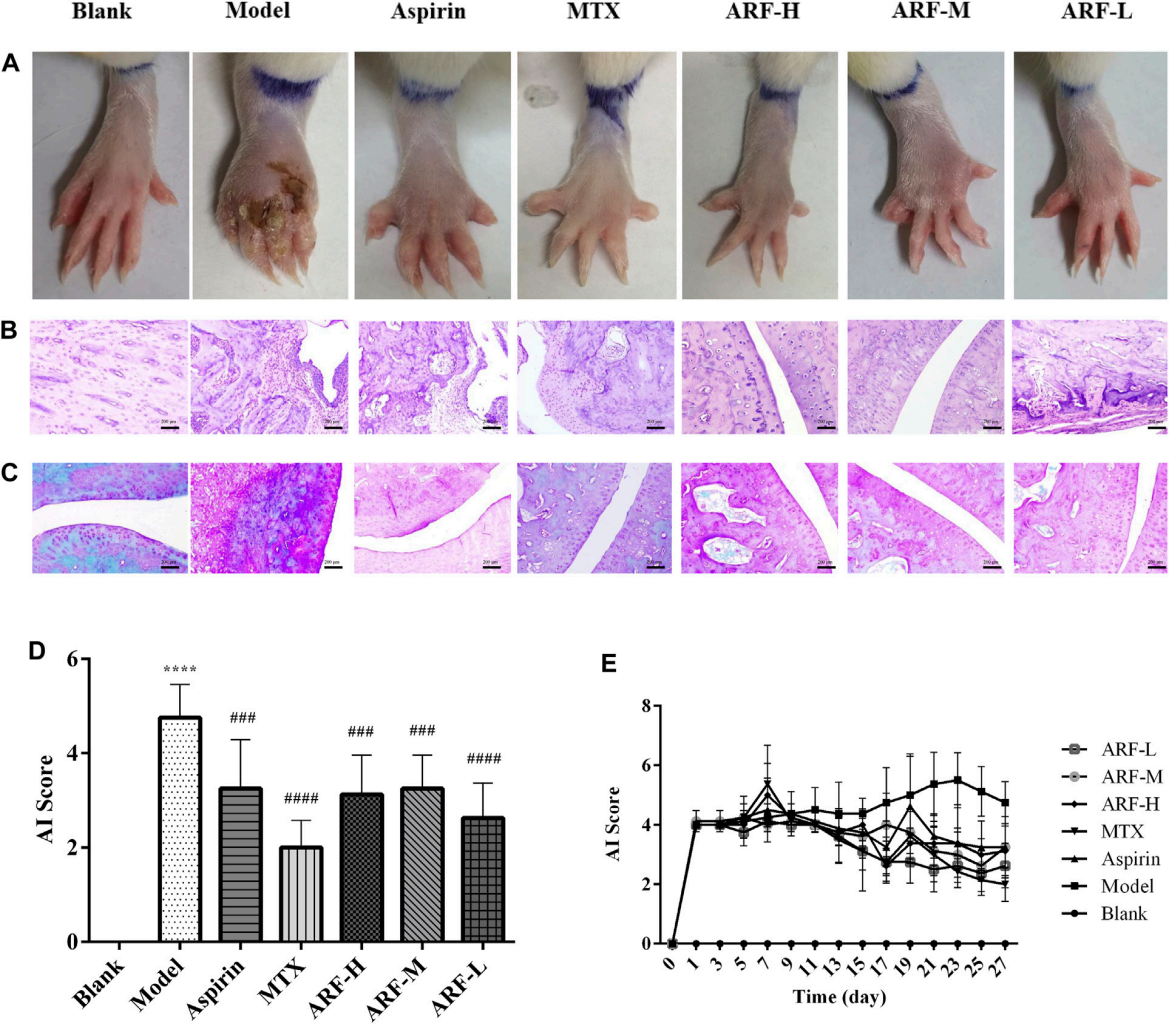
Effect of orally administered ARF on morphological changes and histologic lesions in AIA rats. **(A)** External manifestation of AIA rats on day 27 after immunization, erosion, and red swelling in the paws apparently ameliorated in the ARF-treated group. **(B)** Histological survey of the joints in rats (H&E staining). Original magnification ×100. **(C)** Results of Safranin-O–Fast Green staining in cartilage of joints. Original magnification ×100. **(D, E)** AI scores in joints on day 27 and on every other day, as has been described in the Materials and Methods section, respectively. Data are expressed as mean ± SD (*n* = 8). Comparison with the Blank group, ^****^ *p* < 0.001. Comparison with the Model group, ^###^ *p* < 0.005, ^####^ *p* < 0.001.

**FIGURE 4:**
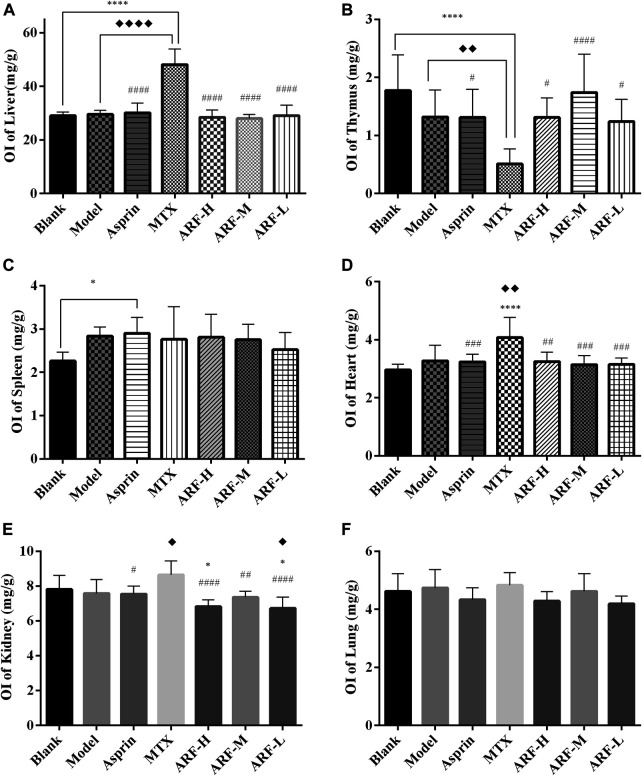
After 21 days of treatment, the effects of each treatment group on the indexes of the thymus, spleen, liver, heart, kidneys, and lungs in AIA model rats. **(A)** liver, **(B)** thymus, **(C)** spleen, **(D)** heart, **(E)** kidneys, and **(F)** lungs. The treatment groups vs. Blank group, ^*^ *p* < 0.05, ^**^ *p* < 0.01, ^***^ *p* < 0.001, ^****^ *p* < 0.001. Data are exhibited as mean ± SD (*n* = 8). Comparison with the Model group, ^◆^ *p* < 0.05. Contrasted with the MTX group, ^#^ *p* < 0.05, ^##^ *p* < 0.01, ^####^ *p* < 0.001.

The FCA-induced AIA rats were used in this study to investigate the therapeutic effect of ARF on RA. The morphological characteristics of arthrophlogosis, such as fester and swelling, could be markedly observed in the Model group. However, the treatment groups obviously relieved arthritis severity in the joints of AIA rats (Figure 3A). Histopathological results (Figures 3B,C) indicate the presence of massive influx of inflammatory cells, synovial hyperplasia, and severe erosion of cartilage and bone in the ankle joints of AIA rats. The symptoms of inflammation and synovial hyperplasia, as well as the joint destruction in rats treated by ARF were significantly alleviated. ARF had a protective effect on cartilage damage, which could relieve the degree of bone damage and cartilage damage seen in AIA rats. In therapy processing, the AI scores were apparently lower in ARF-administrated AIA rats than in the vehicle-treated AIA rats (Figures 3D,E). ARF did no obvious damage to the thymus, spleen, liver, heart, kidneys, and lungs, as could be easily found by comparing the OIs obtained for the AIA model rats (Figures 4A–F). It should be noted that the OI values of the heart, liver, and kidneys of the rats in the MTX group were more than those of the other groups; however, the OI values of the thymus were less than those obtained for the remaining six groups (*p* < 0.05). This indicates that MTX was harmful to these viscera, while ARF was safe for treating AIA rats. These results show that ARF had reduced the AI score, inhibited synovial hyperplasia, ameliorated cartilage damage, and protected the internal organs of the model rats, yet apparently had not depended on the dosage used.

### Effects of Anti-Rheumatic Arthritis Fraction on Production of Cytokines in Adjuvant Induced Arthritis Rats

**FIGURE 5:**
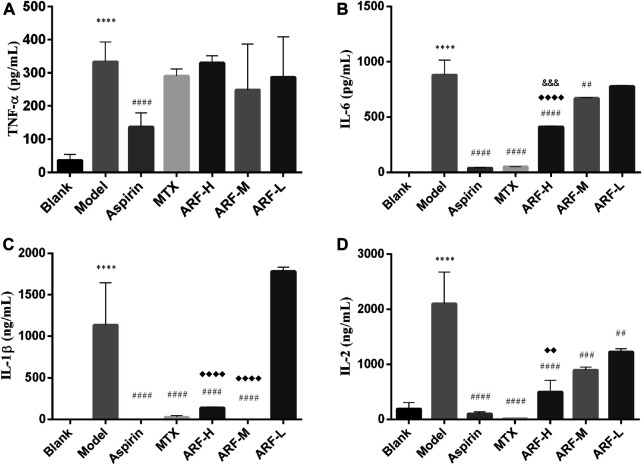
Regulation of ARF on levels of IL-6, TNF-α, IL-1, and IL-2 in AIA model rats after 21 days of treatment. **(A–D)** Levels of TNF-α, IL-6, IL-1β, and IL-2 in the plasma, respectively. ^****^ *p* < 0.001 vs. Blank group. Comparison with the Model group, ^##^ *p* < 0.01, ^##^ *p* < 0.005, ^####^ *p* < 0.001. ^♦♦^ *p* < 0.01, ^♦♦♦♦^ *p* < 0.001, compared with the ARF-L group. ARF-H group vs. ARF-M group, ^&&&^ *p* < 0.005 is considered significant. Data are expressed as mean ± SD (*n* = 8).

Four cytokines were measured by ELISA to assess the effects of ARF on production of pro-inflammatory cytokines in AIA rats. After treating AIA animals for 3 weeks, it was found that except for aspirin, the levels of TNF-α in the groups treated with ARF and MTX were not significantly different from those in the Model group (*p* > 0.05, Figure 5A). There is no significant difference among the three dosages of ARF (*p* > 0.05). The oral administration with high and middle dosages of ARF dramatically repressed the levels of IL-1β and IL-6 in the AIA rats (*p* < 0.01) compared with those of the Model group (Figures 5B,C). Compared with the Model group, the treatment with all three ARF doses obviously reduced the levels of IL-2 in AIA rats (*p* < 0.01) (Figure 5D). Across the board, the effect of ARF was dose dependent for ameliorating the contents of IL-1β, IL-2, and IL-6 in the plasma, suggesting that ARF exerted its action on RA by inhibiting the upregulation of the three cytokine's levels mainly. Interestingly, though the active components of ARF have the same structural unit as aspirin, this action mechanism of ARF is different from that of aspirin, which is more similar to that of MTX.

### Prediction of Absorption, Distribution, Metabolism, and Excretion Properties of Chemical Constituents in Anti-Rheumatic Arthritis Fraction

Considering prediction accuracy, the number of variables, operation time, model stability, and other factors, the optimized five-property prediction models were finally obtained, which were recorded as PGPI-SVM, PGPS-RF + GA, Caco 2-SVM, HIA-SVM, and OB-PLS-DA + GA, respectively. The detailed information in screening and optimization of the QSAR models of the five properties has been referred to in the results provided in Supplementary Tables S1, S3–S13 and Supplementary Figures S1–S8.

The MDs of 47 components in ARF were calculated according to the PaDEL-Descriptor software. About 703 MD variables were excluded by the MATLAB software according to the pretreatment method described in the Supplementary Methods. Finally, a data matrix of 47 × 741 (compound number × MDs) was obtained. After pretreatment of MD and PCA analysis, the ADME properties prediction results of the compounds in ARF were obtained by using the optimal five models of each property established in this study. The detailed data are shown in Table 1. The method of literature validation was applied to verify the ADME properties prediction results of each component in ARF. About 47 chemical constituents and five ADME property names were used as keywords to search the related literature in CNKI (https://www.cnki.net) and PubMed database (NCBI, https://www.ncbi.nlm.nih.gov). The validation results of some compounds are listed in Supplementary Table S13, which is consistent with the prediction results of this study. The overall prediction accuracy rate was 95.56% in comparison with the reference, and demonstrates that the five models are relatively accurate and reliable. There were 25 OB (+) and 22 OB (−), 42 Caco 2 (+) and five Caco 2 (−), 16 PGPS (+) and 31 PGPS (−), 21 PGPI (+) and 26 PGPI (−) components in ARF. The results demonstrate that most of the components in ARF have higher membrane permeability, better intestinal absorption characteristics and better oral bioavailability.

**TABLE 1:** ADME properties prediction results of the compounds in ARF.

Components	Caco 2	PGPS	PGPI	OB	HIA
*p*-Coumaric acid	+^△^	−^△^	−	+^△^	+^△^
Catechol-β-D-glucopyranoside/arbutin	+	+	−	+	+^△^
Vanillic acid	−	+	−	+	+^△^
Citric acid	+	−	−	+	+
3,4,5-Trimethoxybenzoic acid	+	−	−	+	+
Gallic acid	+^△^	−^△^	−	+	+
Protocatechuic acid	−	−	+	+	+^△^
9-Octadecenic acid	+	−	−	+	+
(+)-Catechin	+^△^	−^△^	−	+^△^	+
4-*O*-*p*-coumaroyl quinic acid	+	−	−	+	+
Neochlorogenic acid	+^△^	−	+	+	+^△^
4-Hydroxy-2,6-dimethoxyphenol-1-*O*-*β*-D-glucopyranoside	+	+	−	+	+
Quercetin-3-*O*-rutose-7-*O*-glucoside	+^△^	−	+	+	+
MSTG-B	+	−	+	−	+
Methyl salicylate lactoside/methyl salicylate gentiobioside	+	+	+	−	+
Roseoside	+	+	+	−	+
MSTG-A	+	+	+	−	+
Gaultherin	+	−	−	+	+
Chlorogenic acid	+	−	+	+^△^	+
(−)-Catechin	+	−	−	+	+
5-*O*-*p*-Coumaroyl quinic acid	−	−	+	+	+
(+)-Lyoniresinol	+	−	−	+	+
(+)-Homoeriodictyol	+	−	−	+	+
(−)-Isolariciresinol-2-*α*-*O*-*β*-D-xylopyranoside	+	+	+	−	+
Methyl salicylate vicianoside	+	+	−	−	+
Methyl salicylate-*β*-glucoside	+	+	−	+	+
(+)-Lyoniresinol-2-*α*-*O*-*β*-D-glucopyranoside	+	−	−	−	+
Hesperetin	+^△^	−	+^△^	+^△^	+^△^
Paeoniflorin	+^△^	+^△^	−	−^△^	+^△^
(−)-5′-Methoxyisolariciresinol-2*α*-*O*-*β*-D-xylopyranoside	+	+	+	−	+
(+)-Lyoniresinol-2-*α*-*O*-*β*-L-arabinopyranoside	+	+	+	−	+
Myricetin	+	−	+^△^	+	+^△^
2-Hydroxy-4-methoxyacetophenone	+	+	−	+	+
Quercetin	+^△^	−^△^	+^△^	+^△^	+^△^
Hyperoside	+^△^	−^△^	−	−^△^	+
Fraxinellonone	−	+	+	+	+
Quercetin-3-*O*-glucuronide	+	−	−	−	+
Avicularin	+	−	+	+	+
Kaempferol-3-*O*-*β*-D-glucuronide	+	−	+	+	+
Quercitrin	+^△^	−^△^	−	−^△^	+^△^
4-Hydroxybenzoic acid	+	−	−	+	+
Gaultherin A	+	−	+	+	+
Hexanal/3-hexen-1-ol	+	−	−	+	+
Kaempferol	+^△^	−^△^	+^△^	+	+^△^
Gaultherin C	+	+	−	+	+
Elemicin	+	+	+	+^△^	+
Bornyl acetate/geranyl acetate	−	−^△^	−^△^	−	+

“+”, represents the large category of each property; “−”, represents the small category of each property. “^△^”, represents that the result has been verified in the literature, and the details are displayed in Supplementary Table S14.

### Compound-Common Target Network Analysis

**FIGURE 6:**
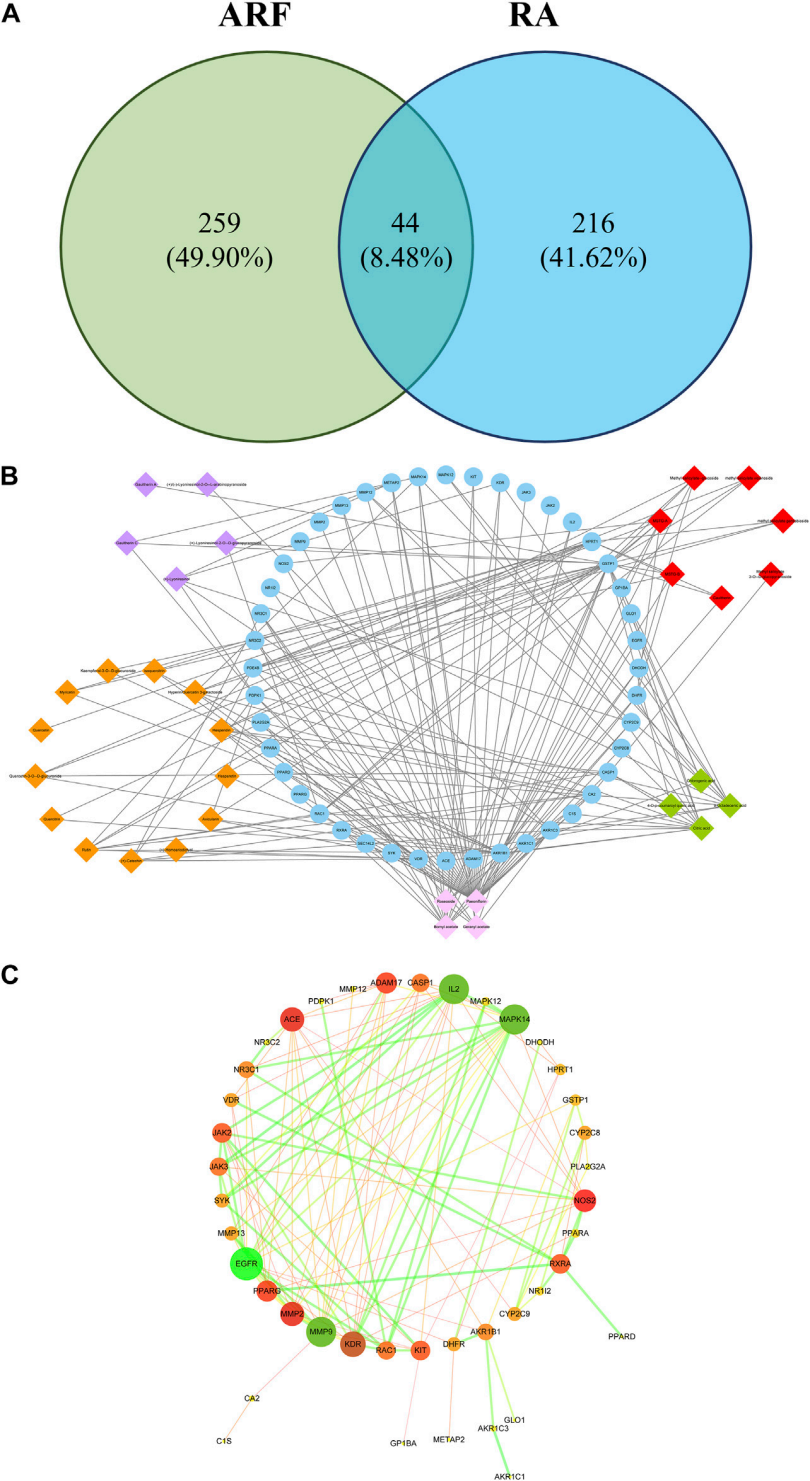
The network analysis of ARF involved in RA progression. **(A)** The relationship of ingredient targets and disease targets. There are 44 overlapping targets between RA and ARF. **(B)** The CCTN. The 33 diamond nodes indicate the active constituents of RA during drug treatment. The 44 blue nodes represent coincident targets between RA and ARF. The edges denote the association between nodes. The orange, red, purple, green, and light pink, respectively, represent flavonoids, methyl salicylate glycosides, lignans, organic acids, and the other-class compounds. **(C)** The overlapping targets of PPI network of ARF. The color and size of the nodes are in directly proportion to DV, and the color and thickness of the bridge line are proportionate to the values of the combined score. The larger and darker the node, the more important position it occupied in this PPI network. The thicker and darker the edge, the stronger the interaction between the nodes.

A total of 44 overlaps of 260 targets were noted for RA and 303 targets for ARF (Figure 6A), indicating that they may be the key for ARF in RA therapy. To show how ARF acts on RA, the targets of the active compounds in ARF, together with the common targets network (for short CCTN) are displayed in Figure 6B. The 33 active constituents with a therapeutic effect on RA have been identified. The 44 blue nodes represent overlapping targets between RA and ARF. The diamond nodes represent the active constituents in ARF: the red, orange, purple, green, and pink diamond nodes represent methyl salicylate glycosides (7), flavonoids (13), lignans (5), organic acids (4), and the other category components (4), respectively. The edges indicate the influence between the nodes with each other.

The network centralization and heterogeneity were 0.476 and 1.222, respectively, suggesting that the contribution of some nodes for this network was bigger than that of the others. It was reckoned that these nodes may be pivotal and merit further investigation. As shown in Supplementary Table S15, several components had multiple targets, such as paeoniflorin (degree = 40), bornyl acetate (degree = 16), citric acid (degree = 13), 9-octadecenic acid (degree = 10), hesperidin (degree = 8), and rutin (degree = 8). Simultaneously, the same target was affected by multi-compounds, for instance, GSTP1 (degree = 25), AKR1B1 (degree = 18), VDR (degree = 12), and HPRT1 (degree = 10). These findings reveal that the multidimensional effect of ARF in relieving RA is by the style of one ingredient–multiple targets and one target–multiple ingredients. It needs to be illustrated that due to the limitation of the database and existing literature, the number of targets of paeoniflorin was significantly more than that of the other compounds. This is not only the advantage of using the network pharmacology method, but also a shortcoming, indicating that these components and targets should be validated by an in-depth study.

### Common Target Protein-Protein Interaction Network Analysis

In this article, a PPI network, including the common 44 target proteins of ARF and RA disease, was established to appraise the cellular functions and processes of ARF. A detailed information is provided in Supplementary Table S16. The Cytoscape software was used to perform visualization and topology analysis for this PPI network. The combined score and DV were used to evaluate the size of edges and nodes. In removing the two disconnected nodes (PDE4B and SEC14L2), the results showed that this network contained 42 nodes and 125 edges (Figure 6C). According to the results of the topology analysis, the nodes in the PPI network, whose three corresponding indices were higher than the average DV 5.95), node betweenness centrality (NBC, 3.97 × 10^−2^), and node closeness centrality (NCC, 0.40), were regarded as the main nodes. A total of 10 targets, including EGFR, MMP 9, IL-2, Mapk14, KDR, and the other five proteins (as shown in Table 2), were selected finally. It is reckoned that these 10 targets are likely to be the core targets for ARF in exerting the anti-RA effect. As a result, the 10 crucial nodes were chosen for enrichment analyses of the GO and KEGG pathway.

**TABLE 2:** The topological parameters of the 10 key targets.

Target name	Gene name	DV	NBC	NCC
Epidermal growth factor receptor	EGFR	17	2.40E-01	5.77E-01
Matrix metalloproteinase-9	MMP9	15	1.54E-01	5.32E-01
Interleukin-2	IL2	15	9.11E-02	5.26E-01
Mitogen-activated protein kinase 14	MAPK14	15	5.74E-02	5.06E-01
Vascular endothelial growth factor receptor 2	KDR	12	5.28E-02	5.00E-01
Angiotensin converting enzyme	ACE	11	1.53E-01	5.32E-01
Nitric oxide synthase, inducible	NOS2	10	5.60E-02	4.82E-01
ADAM17	ADAM17	9	4.54E-02	4.66E-01
Mast/stem cell growth factor receptor kit	KIT	8	6.45E-02	4.23E-01
Aldose reductase	AKR1B1	6	1.57E-01	4.46E-01

### KEGG Pathway Enrichment Analysis

**FIGURE 7:**
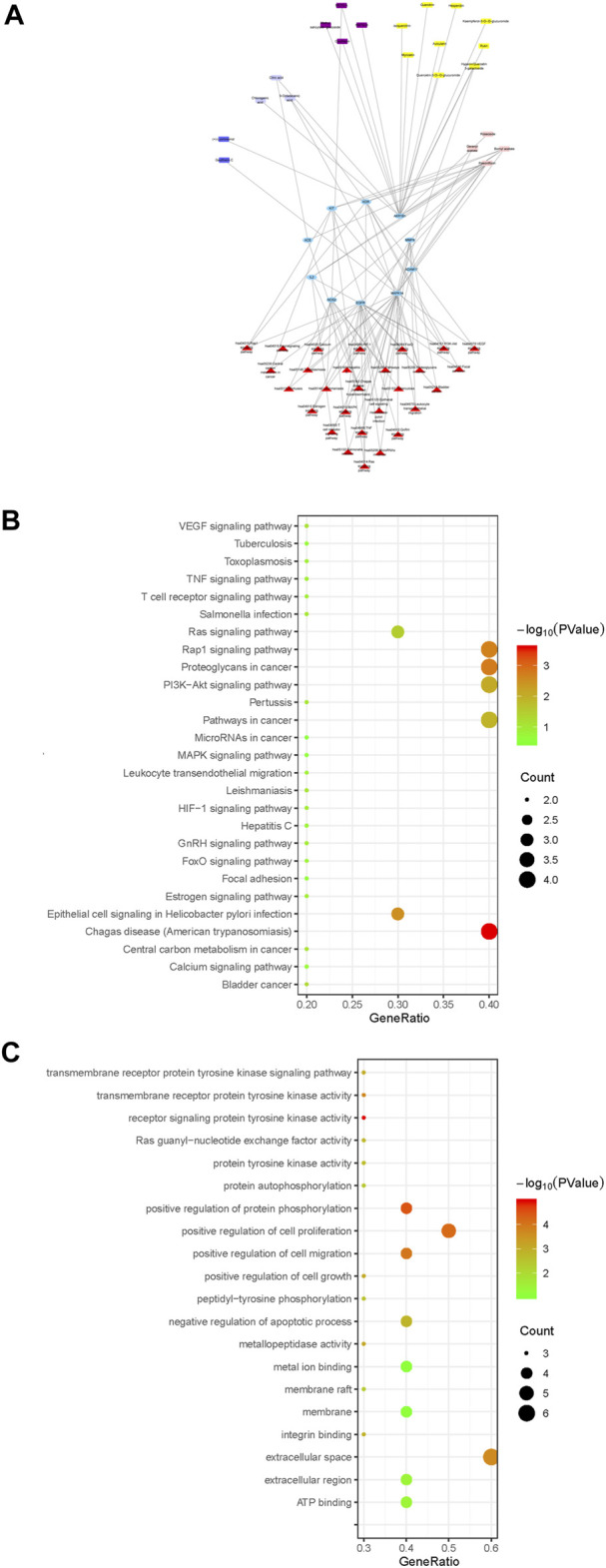
The profile of the ingredients–targets–pathways relationships of ARF. **(A)** Components–core target–pathway network of ARF. Blue elliptical nodes represent the key target, red triangles represent pathways, the other colors represent different types of components in ARF (yellow, purple, blue, lavender, and light pink rectangle, respectively, represent flavonoids, methyl salicylate glycosides, lignans, organic acids, and other compounds). **(B)** KEGG pathway analyses of the ARF key target genes. The vertical and horizontal axes represent the pathway name and enrichment factors, respectively. The size of the dots demonstrates the amount of targets enriched. The color of the circular dots represents the *p*-value, and red to green indicate *p*-values from small to large. **(C)** GO analysis of ARF key target genes. The vertical axis is the name of the GO items, and the horizontal axis the enrichment factor. The size of the points indicates the number of the targets enriched. The color of the dot represents the *p*-value, and red to green represent *p*-values from small to large.

Cytoscape 3.6.2 software was used to draw the component-key target-pathway network diagram of ARF, as shown in Figure 7A. The signaling pathways closely related to 10 core targets and RA were filtered from KEGG on DAVID platform to fully appreciate the function and role of ARF in ameliorating RA. A total of 27 pathways were obtained by KEGG enrichment, and the bubble diagram of KEGG pathway were drawn in Figure 7B. With *FDR* < 0.05 as the critical value, seven signal paths were screened out (Supplementary Table S17). The key targets primarily related to those signaling pathways, such as Proteoglycans in cancer (Fold enrichment = 13.8, *p* < 0.01) and Rap1 Signaling pathway (Fold enrichment = 13.1, *p* < 0.01), PI3K-Akt signaling pathway (Fold enrichment = 7.98, *p* < 0.01) and Ras signaling pathway (Fold enrichment = 9.13, *p* < 0.05). These results suggested that ARF may act on these signaling pathways to achieve the treatment purpose for RA.

Different types of compounds in ARF have been represented by different colors and the component–core target–pathway network drawn (Figure 7A). The main chemical composition types related to the core target and pathway were found to be flavonoids and methyl salicylate glycosides in ARF, with nine and four components, respectively. Moreover, three organic acids, two lignans, and four other components were slightly related to the core target and pathway. The results showed that flavonoids and methyl salicylate glycosides might be the key components for the anti-RA effect of ARF. It has been verified that the pharmacological action of ARF is produced by multiple components and multiple targets. These constituents and targets can partly or contemporaneously have an effect on similar or identical biological processes. As shown in Figure 7B, there were four target genes each in the KEGG pathway analyses of proteoglycans in cancer, Rap1 signaling pathway, and PI3K-Akt signaling pathway. The epithelial cell signaling in the *Helicobacter pylori* infection pathway and the Ras signaling pathway contained three target genes each. Two target genes were present in other pathways such as for bladder cancer. Particularly, according to the above-mentioned network pathway analyses and published literature, it was found that the critical core proteins or pathways are related to cell physiological processes or inflammation (Chen et al., 2020; Ney et al., 2020; Liu et al., 2021). For instance, PI3K/AKT, Rap1, and Ras signaling pathways have a strong relationship with the occurrence and development of inflammation progressing, which can regulate the balance of pro-inflammatory and anti-inflammatory factors, along with perpetuation or mitigation of inflammation in tissues (Shah et al., 2018; Liu et al., 2021). Thus, it is reckoned that ARF relieved RA by anti-inflammatory effects; therefore, the five key targets were selected for the next experiment to validate this point.

### Gene Ontology Enrichment Analyses

The GO analysis function of the DAVID V6.8 database was used to carry out enrichment analysis on 10 core targets for the anti-RA effect of ARF of Dianbaizhu. About 63 GO items (including 42 BP, 13 MF, and 8 CC items) were obtained (Supplementary Table S18), and the GO bubble diagram derived as shown in Figure 7C. The *p*-value was calculated by Fisher’s exact test algorithm applied to rank the GO enrichment results in order to identify and distinguish their significance from each other. In addition to the regulation of cytokines involved in inflammatory reaction and protein self-phosphorylation, the BP is mainly related to the positive regulation of the cell cycle protein serine/threonine kinase activity, protein phosphorylation, G1/S transition of the mitotic cell cycle, as well as cell growth, proliferation and migration, together with neutrophil-mediated immunity (as shown in Supplementary Table S18). The MF is mainly involved in various protein binding, enzyme activity, ATP binding, receptor activation, and other aspects. The CC includes extracellular space, membrane rafts, endosomes, mast cell granules, the perinuclear area, extracellular regions, information nodes, membranes, and so on. With *p* < 0.01 and FDR <0.05, two GO items were further determined, as shown in Supplementary Table S18. Among these, there is one item related to biological processes, which mainly involves positive regulation of protein phosphorylation (GO ID: 0001934, Fold enrichment = 506, *p* < 0.001). There is one item related to molecular function, which is the receptor signaling protein tyrosine kinase activity (GO ID: 0004716, Fold enrichment = 52.9, *p* < 0.001). Taken together, it is speculated that the anti-RA effect of ARF is mainly bound with the biological processes of protein phosphorylation and the activity of receptor signal protein tyrosine kinase.

### Anti-Rheumatic Arthritis Fraction Restrained IL-1β-Induced Proliferation in HFLS-RA

**FIGURE 8:**
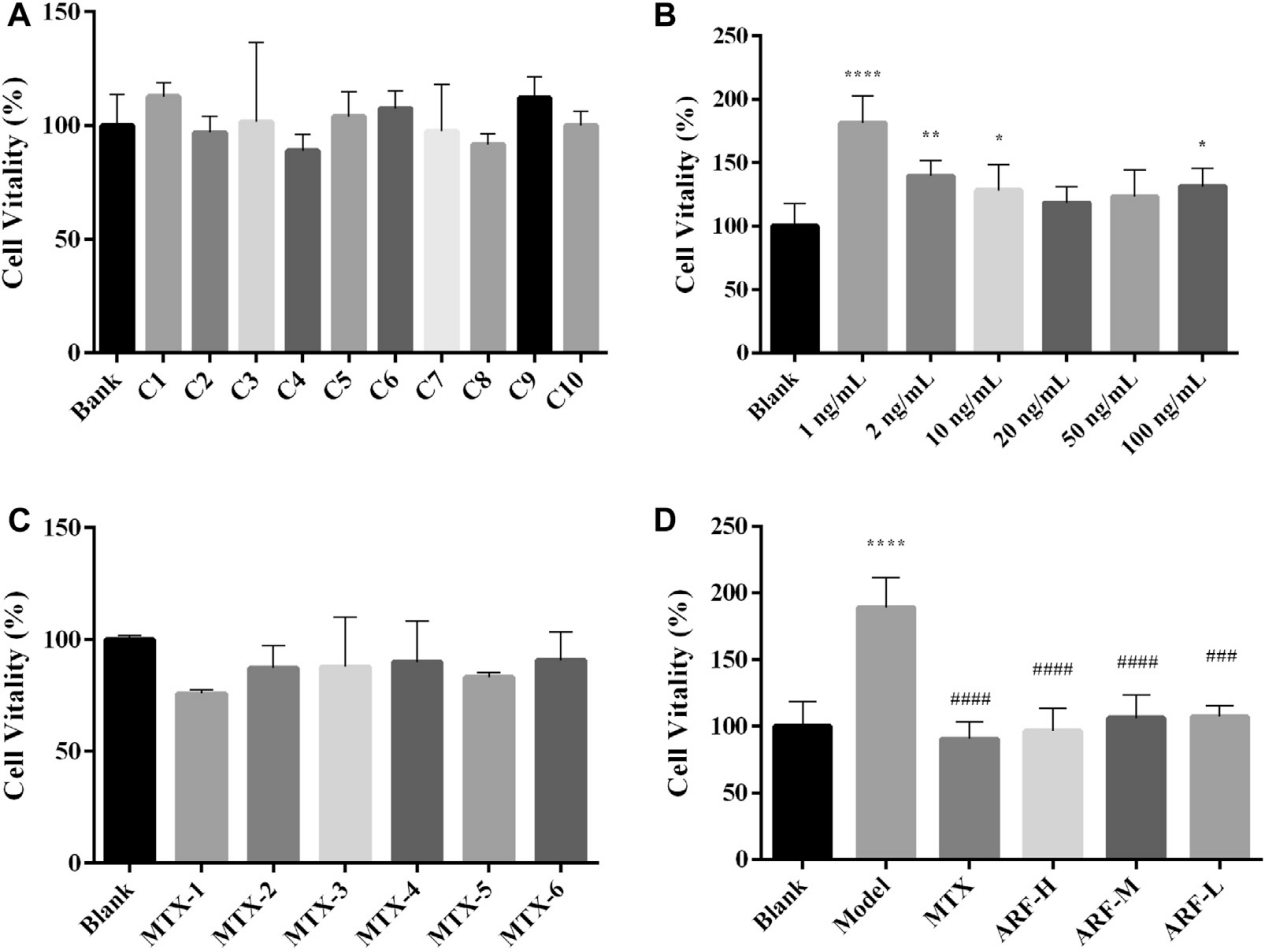
Effects of ARF and MTX on cell viability of HFLS-RA with or without IL-1β stimulation. **(A)**: HFLS-RA cultured with different concentration (3.3252–0.0065 mg/ml) of ARF for 48 h. **(B)**: IL-1β-induced proliferation in HFLS-RA were investigated after culturing 48 h. **(C)**: HFLS-RA incubated with six concentrations of MTX for 48 h. **(D)**: IL-1β-stimulated HFLS-RA were treated with ARF-H (1.6626 mg/ml), ARF-M (0.4157 mg/ml), ARF-L (0.0519 mg/ml) or MTX (1 × 10^−5^ μM) for 48 h. Results are showed as mean ± SD (*n* = 6). Cell viability was monitored by MTT assay. Blank group represents the cells without drug treatment. ^*^ *p* < 0.05, ^**^ *p* < 0.01, ^****^ *p* < 0.001, versus Blank group; ^###^ *p* < 0.005 and ^####^ *p* < 0.001, versus IL-1β-stimulated group (Model group).

It is widely known that the IL-1β–stimulated HFLS-RA model is used to develop intermediary target for RA therapy. The effect of IL-1β (1, 2, 10, 20, 50, and 100 ng/ml) on the proliferation of HFLS-RA was first investigated with cell viability as the index. As shown in Figure 8A, 1, 2, 10, and 100 ng/ml IL-1β stimulation apparently enhanced the cell proliferative viability of HFLS-RA (compared with the Blank group, *p* < 0.001, 0.01, 0.05, and 0.05, respectively), indicating that IL-1β can induce HFLS-RA proliferation. In this present study, IL-1β (1 ng/ml) incubation with the cells for 48 h was selected to finally induce inflammation. The potential cytotoxic effects of ARF on HFLS-RA *in vitro* were tested for safety and effectivity. As shown in Figures 8B,C, the results of the MTT assay illustrated that treatment with ARF (0.0065–3.3252 mg/ml) and MTX (1 × 10^−6^ to 5 × 10^−3^ μM) had no remarkable inhibition capability on cell viability of HFLS-RA. ARF (0.0519–1.6626 mg/ml) exhibited prominent suppression on IL-1β–induced hyperplasia of HFLS-RA (compared with the Model group, *p* < 0.001, Figure 8D). The findings demonstrate that ARF can safely and effectively restrain proliferation of IL-1β–induced HFLS-RA in a dose-dependent manner.

### Anti-Rheumatic Arthritis Fraction Regulates EGFR, MMP9, IL2, MAPK14, and KDR Expression

**FIGURE 9:**
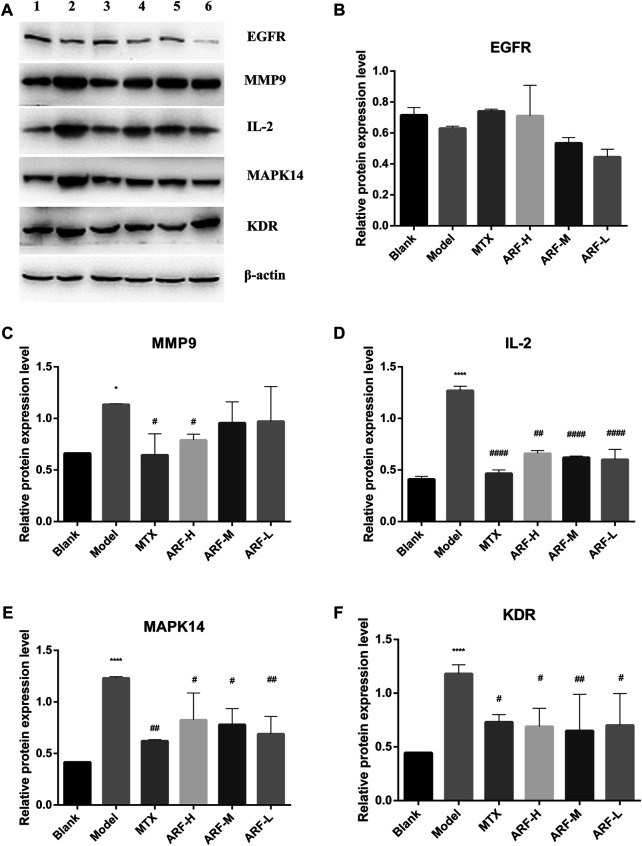
Consequences of ARF on the five key target proteins production of IL-1β–induced HFLS-RA. **(A)** Expressions of EGFR, MMP9, IL2, MAPK14, and KDR protein detected by Western blot (1, Blank group; 2, Model group; 3, MTX group; 4, ARF-H group; 5, ARF-M group; 6, ARF-L group). **(B)** Effect of ARF on the expression of EGFR. **(C)** Effect of ARF on the expression of MMP9. **(D)** Effect of ARF on the expression of IL2. **(E)** Effect of ARF on the expression of MAPK14. **(F)** Effect of ARF on the expression of KDR. ^*^ *p* < 0.05 and ^****^ *p* < 0.001 stand for notable differences in comparison to the control group (Blank group); ^#^ *p* < 0.05, ^##^ *p* < 0.01, or ^####^ *p* < 0.001 confirm a striking difference compared with the IL-1β–stimulated group (Model group).

According to the order of DV, NBC, and NCC values in Table 2, together with the importance and relevance in the process of suppressing RA, five target proteins, namely, EGFR, MMP9, IL2, MAPK14, and KDR, were selected to verify the outcomes of network pharmacology analyses and the mechanism of action of ARF in remedying RA. Western blot was adopted to monitor the expression levels of EGFR, MMP9, IL2, MAPK14, and KDR. The bands are shown in Figure 9A. Gray scale analysis results are shown in Figure 9B–9F. The levels of MMP9, IL2, MAPK14, and KDR in HFLS-RA in the Model group were quite higher than those in the Blank group (*p* < 0.05). The expression of IL-2, MAPK14, and KDR in ARF-L, ARF-M, ARF-H, and MTX groups was decreased (*p* < 0.05). The expression levels of MMP9 in ARF-H and MTX groups were significantly downregulated (*p* < 0.05). More interestingly, compared with that of the Blank group, the expression level of EGFR in IL-1β–induced HFLS-RA was reduced without significance (*p* > 0.05). The expression level of EGFR in ARF-H and MTX group was upregulated after operation, and the expression level was still less than that found for the Blank group (*p* > 0.05). The expression content of EGFR in the ARF-M and ARF-L groups was not obviously upregulated compared with that in the ARF-H group (*p* > 0.05). The data shows that ARF could effectively downregulate the content of MMP9, IL2, MAPK14, and KDR in HFLS-RA, prompting that ARF may alleviate the symptoms of RA by targeting MMP9/IL2/MAPK14/KDR proteins.

## Discussion

Currently, the main therapeutic approaches for RA are basically focused on relieving symptoms and changing the way of life. Over the past two decades, from relying on broadly active traditional medications, the focus has now shifted toward more specific agents that often target a single receptor, cytokine, or cell type, using monoclonal antibodies, fusion proteins, or targeted small molecules (Davis et al., 2020). This change has transformed the treatment of RA, but along with the benefits have come risks, such as infectious complications (Schett et al., 2020). There is no effective strategy on developing more new drugs for the cure of RA at present. It is considerably difficult to interpret and reveal the mechanisms of TC/EMs because of their complicated and varied chemical compositions. With the combination of ML and network pharmacology, a TC/EM development strategy based on global tracking orientation from compounds to mechanisms has been proposed and needs to be further explored. This strategy coincides with the overall development of TC/EMs. Dianbaizhu, as one of Chinese herbs, has been applied in treating RA for a long time. The systematic pharmacological estimation of the curative effect of ARF on RA was not heretofore investigated.

The disassemble research framework of this study and the relationship between them, as well as the crucial results, were shown in Graphical Abstract. In the present study, the components of ARF were firstly identified by using UHPLC-LTQ-Orbitrap-MS^n^ in the negative ion mode. A total of 48 compounds were characterized in ARF. Subsequently, according to the ADME properties data of known compounds, the QSAR models of ADME properties prediction or classification were established by a series of statistical methods. The ADME parameters of these ingredients in ARF were evaluated using the QSAR models. It has been indicated that ARF had eminent potential biological and pharmacological activities, evincing an excellent drug likeness. In the modeling process, it should be noted that most of the models based on PCA total data set had a long calculation time and poor quality. Therefore, the data matrixes, named PCA-80, were adopted to research on training and modeling by each algorithm. Thus, a total of 50 ADME properties classification prediction models with reliable and correct statistics have been developed. In addition, the simulation results of practical examples have testified that compared with the five single classifiers, the method of the five single classifiers trained by the GA can improve the calculation accuracy and the speed of the convergence process of the models. The results also confirm that the type of properties and single classifiers both have a certain influence on improving the scalability of the GA for the models. Regrettably, among these properties and single classifiers, only RF combined with the GA was more effective in classifying the PGPS of compounds, which plays an important role in QSAR model establishment. The appropriate selection of descriptors by the GA is helpful to the scalability of the models. Too many variables in the model not only have a large amount of calculation, but also may lead to the instability of the side model. In order to solve this problem, variable screening should be carried out. The GA can deal with any form of objective function and constraint, whether linear or nonlinear, discrete or continuous (Peng et al., 2014). It is suggested that the interaction among multiple factors should be comprehensively analyzed and compared for the QSAR model structure in the future, not just a simple technical superposition. Computational approach and models used in this study could be used in predicting the ADME performance of other drugs in future.

The underlying action mechanisms and experimental verification *in vitro* of ARF on RA through network pharmacology prediction were explored. It is worth mentioning that rutin and isoquercitrin can be detected in ARF incubated by simulated gastrointestinal liquid and human intestinal bacteria *in vitro* (Wang et al., 2019). They are the main metabolites of flavonoids in ARF. They have been reported to have significant anti-RA effects (Adhikary et al., 2018; Teixeira et al., 2020). Therefore, based on the idea of ARF playing a role as a whole, rutin and isoquercitrin were also selected as the main constituents to promote the investigation of treatment target prediction and validation of ARF on RA. About 10 direct or indirect therapeutic core targets of ARF for RA treatment were screened through PPI network topology analyses, such as EGFR, MMP 9, IL-2, MAPK14, KDR, ACE, NOS2, ADAM17, KIT, and AKR1B1. It has been speculated that they may promote the improvement of RA at the gene level through the protein phosphorylation process and by regulating receptor signal protein tyrosine kinase activity. Peculiarly, recent studies have confirmed that these targets play a variety of roles in RA (Ren et al., 2019). The correlation between these five core targets and the inflammatory factors also verified the accuracy and rationality of the results in the *in vivo* experiment, that is, ARF can play an anti-RA role by downregulating the contents of IL-6, IL-1, IL-2, and TNF-α in the plasma of AIA model rats. The results suggest that ARF can affect the occurrence and development of RA by regulating the expression of these five targets, and thereby play a therapeutic role.

Moreover, there are few KEGG pathways enriched by the core target/gene of ARF treatment on RA, mainly including proteoglycans in cancer, the Rap1 signaling pathway, the PI3K-Akt signaling pathway, pathways in cancer, the RAS signaling pathway, and so on (Supplementary Table S17). It has been reported that Ras and PI3K-Akt signaling pathways were closely related to RA by activating various downstream target proteins and making inflammation persist in tissues due to induced apoptosis, respectively (Fu et al., 2020). It is speculated that the key target of ARF plays a synergistic role in anti-RA by participating in multiple pathways. Additionally, the number of enriched pathways and targets were relatively small in general, which may be related to the fact that the chemical components of ARF are rarely reported in the literature. This also shows that there are some limitations in the database of TC/EMs network pharmacology research. There are many open source databases that can be used, but it also increases the difficulty and inaccuracy of target and pathway prediction. In this article, the flavonoids and methyl salicylate glycosides were more related to the core target and pathway, followed by other components, organic acids, and lignans in ARF. The result reflects the way of interaction among multiple components, suggesting the importance of flavonoids and methyl salicylate glycosides of the ARF against RA effect. These component types are basically consistent with those of ARF and its metabolites in the gastrointestinal tract, which indicates that ARF as a whole plays the role of anti-RA. This integrity has been reflected in the multiple component–target–pathway. The results clearly show us that ARF exerts its pharmacodynamics effect on RA through methyl salicylate glycosides, flavonoids, organic acids, lignans, and other components on 10 core targets from the PI3K-Akt signaling pathway, Ras signaling pathway, and other pathways. This research may raise a new insight and preclinical basis in preventing and treating RA.

## Conclusion

In this work, 48 major constituents of ARF were characterized by using UHPLC-LTQ-Orbitrap-MS^n^. The 50 QSAR models on the ADME properties of these constituents were established on the basis of the ML algorithms. The final optimized model of the five properties was successfully applied to predict the biological activity of ARF. The results showed that the GA was not beneficial to every ML in building and screening the QSAR models, and its advantages depend more on the properties of the components alone. It also needs to be further assessed by extending the number of compounds in the future. The network pharmacology analyses of ARF suggests that methyl salicylate glycosides and flavonoids in ARF are more related to treatment of RA (Figure 7A). Further experimental validations also offered convincing evidence that ARF may attenuate RA partially by restoring the expression level of EGFR, MMP 9, IL2, MAPK14, and KDR and in reversing the pathological events during RA progression by regulating inflammatory factors. *Gaultheria leucocarpa* var. *yunnanensis* is a folk medicine that the research group of this study have been developing in the past decade, but its pharmacodynamics constituents and mechanism have not been excavated. In the present study, the anti-RA compounds as well as their potential mechanisms were defined, which can be potentially used as a therapeutic option in the treatment of RA without side effects.

For the first time, this investigation offered an integrative analysis by combining drug target and ADME properties prognosis with network analyses to expound the pharmacological mechanisms of ARF in relieving RA. This systematic research is helpful in revealing the rule of TC/EMs of this complex disease of unknown etiology. Owing to the approaching graduation time and unexpected situation with COVID-19, the corresponding ADME properties predicted by the QSAR models in this study, together with the related key targets and pathways predicted by network pharmacology on the anti-RA effect of *G*. *yunnanensis* have not been fully tested and verified, which will be further investigated in a follow-up research of this study.

## Data Availability

The original contributions presented in this study are included in the article/Supplementary Material, further inquiries can be directed to the corresponding authors.

## Glossary

ARF: anti–rheumatic arthritis fraction
ADME: absorption, distribution, metabolism, and elimination
PCA: principal component analysis
TRS: training set
TES: test set
VAS: validation set
GA: genetic algorithm
FBS: fetal bovine serum
PGPI: P-gp inhibitor
PGPS: P-gp substance
HIA: human intestinal absorption
OB: oral bioavailability
QSAR: quantitative structure–activity relationship
ML: machine learnings
RF: random forest
KNN: K-nearest neighbors
PLS-DA: Partial Least Squares-Discriminant Analysis
EBPT: error back propagation training
SVM: support vector machine
SMOTE: synthetic minority oversampling technique
IR: imbalance index
LOO: leave-one-out Cross
5 FCV: 5 folds cross validation
AUC: area under the curve
RMSE: root mean square error
ER: error rate
MCC: Matthew's correlation coefficient
OA: overall prediction accuracy
SE: sensitivity
SP: specificity
ROC: receiver operating characteristic curve
AD: application domain
SR: standardized residuals
HFLS-RA: human fibroblast-like synoviocytes of rheumatoid arthritis
DV: degree value
NDD: node degree distribution
NBC: node betweenness centrality
NCC: node closeness centrality
MSTG-B: methyl benzoate-2-O-β-D-glucopyranosyl(1→2)-[O-β-D-xylopyranosyl(1→6)]-O-β-D-glucopyranoside
MSTG-A: methyl salicylate-2-O-β-D-xylopyranosyl(1→2)-[O-β-D-xylopyranosyl(1→6)]-O-β-D-glucopyranoside
Gualtherin: methyl salicylate-2-O-β-D-xylopyranosyl(1→6)-O-β-D-glucopyranoside
