# Supplementary material for: Gaultheria leucocarpa var. yunnanensis for Treating Rheumatoid Arthritis—An Assessment Combining Machine Learning–Guided ADME Properties Prediction, Network Pharmacology, and Pharmacological Assessment
Source: Front Pharmacol. 2021 Oct 4;12:704040. doi: 10.3389/fphar.2021.704040 (PMC8520986; doi:10.3389/fphar.2021.704040)
Supplement: Supplementary file 2 [file DataSheet1.docx]

**Supplementary Material**

**Supplementary methods**

**ADME evaluation**

***Data collection***

In this study, a large and diverse data sets from some published articles and online dataset on the 5 ADME properties were collected and carefully validated the quality of the data sets from multiple sources. After a series of pretreatments, 1100 compounds and their properties values were finally collected as further analysis.

*PGPS data*

The P-gp substrate data for this study are primarily derived from 1700 collected compounds published literature [1-5]. Excluding repetition, a total of 837 compounds were obtained. According to whether they are PGPS, the compounds were divided into two types: PGPS (+) and PGPS (-), which have 190 and 647 compounds respectively (As shown as in Table S1).

*PGPI data*

Data on PGPI were obtained from the papers published by the previous research [1, 5-9]. A total of 851 compounds were obtained by excluding duplicates. According to whether they are PGPI or not, they were divided into two categories: PGPI (+) and PGPI (-), with 295 and 556 compounds respectively.

*Caco 2 data*

The data on the Caco 2 cell permeability of compound were mainly collected from 6 published literatures [10-15] and an online data [5]. The dataset was filtered to remove compounds with permeability values greater than 10^-4^ cm/s or less than 10^-8^ cm/s because of their potential unreliability. With the value of permeability coefficient (2 × 10^-6^ cm/s) as the critical value, all compounds were and divided into two categories, represented by Caco 2 (+) and Caco 2 (-), and there were 102 and 648 compounds respectively.

*HIA data*

HIA data for compounds studied in this section were derived from data collected from 650 compounds modeled in the ADMETlab database [5]. According to the references, 30% is used as the threshold value of HIA to classify the categories, which are respectively called HIA (+) and HIA (-), which contain 573 and 77 compounds respectively.

*OB data*

The oral bioavailability plays a critical role in the efficiency of drug distribution to systemic circulatory system, which directly determines the pharmacodynamics parameters. The compound OB data in this section is mainly derived from more than 50 papers: including ADMETlab database [5] and other scientific research teams [16] [17]. A total of 871 compounds were collected, with 30% as OB threshold, and the collected compounds were divided into two categories. If OB of a compound is less than or equal to 30%, it is classified as OB (-), otherwise it is OB (+). There are 486 and 385 compounds respectively.

To sum up, all data of the 5 characteristics are summarized, and the general table is shown in Table S1. The imbalance index (IR) of all properties is calculated to describe the distribution of the data set. According to the definition in the literature [18], when IR > 1.5, it is judged as unbalanced data. As shown as in Table 1, only the OB data set has a relatively small IR value, while the other four properties data shown a strong imbalance, especially the Caco 2 permeability and HIA data set. Therefore, in order to prevent the overfitting of the model, use the synthetic minority oversampling technique (SMOTE) to improve the imbalance of the data [19, 20].

Table S1 Summary of the data set information and IR values of 5 ADME properties

| Data sets | The number of compounds | The number of MD | Major compound / (+) | Subclass compound / (-) | IR |
| --- | --- | --- | --- | --- | --- |
| PGPS | 837 | 1444 | 190 | 647 | 3.40 |
| PGPI | 851 | 1444 | 295 | 556 | 2.15 |
| Caco 2 | 750 | 1444 | 102 | 648 | 6.35 |
| HIA | 650 | 1444 | 573 | 77 | 7.44 |
| OB | 871 | 1444 | 486 | 385 | 1.26 |

***MD calculation and pruning***

One-dimensional (1D) and two-dimensional (2D) molecular descriptors (MDs) were used to be the molecular features to describe all the compounds. Firstly, all the compound information was integrated, and the 2D structures of them were saved into the SMILES format. PaDEL-Descriptor software was applied to generate 1D and 2D MDs [21]. A total of 1,447 MDs were obtained for each compound. Secondly, all MDs were checked to ensure that each descriptor value is available for each molecule. Two pretreatments were performed to delete some uninformative descriptors before further selection: 1) delete the descriptors whose variance is 0 or approaches 0 [22]; 2) if the correlation coefficient between two descriptors is higher than 0.90, only one was reserved [23]. Finally, 741 descriptors were used to further perform variable selection and QSAR modeling.

***Data dimension reduction processing***

All the calculated molecular features cannot be used for the QSAR modeling due to the curse of dimension for high-dimensional data and the phenomenon of over-fitting. Feature selection has been widely used for feature preprocessing in machine learning. Removing irrelevant and redundant information can improve the performance of models. Using Matlab 2017a software, the MDs and ADME properties parameters of various compounds were both analyzed by principal component analysis (PCA). The rationality of subset selection of data was investigated by observing the distribution of data set samples in three dimensional. The MDs with a relatively large correlation with 5 properties parameters were selected as the basis for the next analysis [24].

***Data splitting strategy***

A three-dataset (training/validation/test datasets) splitting strategy was used and implemented with Randperm Function in Matlab 2017a software. The data was divided into three subsets in this paper: Training Set (TRS, 50%), Test Set (TES, 25%) and Validation Set (VAS, 25%). The TRS is for training models, and the VAS is for tuning hyper-parameters to find the best model. The accuracy of the TES shows the prediction ability on unknown data.

***Modeling Method***

Five machine learning methods with or without GA were introduced to construct QSAR models, including SVM, RF, KNN, PLS-DA and EBPT [25-29]. Firstly, these classification models were trained using the related package with Matlab 2017a. The variables involved in the model construction were evaluated and ranked based on the prediction results. Subsequently, for variable selection issue, the binary coding form of each chromosomeis adopted with 1 and 0 representing selected and non-selected descriptors, respectively. The different number of the important variable were screened out for each ADME properties by GA. The optimal models were preliminary selected with the accuracy, and were used to predict TES data [30]. Then, GA was combined with 5 algorithms to repeat the previous steps. The prediction property of the models constructed by different numbers of the MDs were statistically analyzed to achieve the optimal descriptor set. Based on the chemical space characterized by the optimal descriptor set, hierarchical clustering method was used to conduct clustering research on the compounds in TRS. Finally, the final result was optimal solutions and mutual independence, and the QSAR models were derived from Matlab software for the properties [31]. The GA parameters were set as follows: single point crossover probability and single point mutation probability were set as 0.8 and 0.05, respectively; the initial population size was set as 40 ~ 50; the maximum evolutionary algebra and random search were set as 50 and 50, respectively.

***Model evaluation and validation***

The models were all preliminarily evaluated and screened by synthesizing the parameters. Their forecast ability of TRS, TES and VAS data subsets before and after GA optimization were comprehensive evaluated. [32] . In order to ensure those models derived from the training set has good generalization ability, the leave-one-out Cross Validation (LOO) and 5 folds Cross Validation (5 FCV) were utilized for the evaluation and validation purpose. In the process of screening variables with GA, LOO was used to calculate fitness function. In order to improve the limitations of LOO method, irrelevant test set method and 5 FCV method were used to jointly evaluate the predictive ability of the models [33]. The main model evaluation parameters include: Precision, Recall, F-measure, root mean Square error (RMSE), determination coefficient (R^2^, R-square), error rate (ER), Matthew's Correlation Coefficient (MCC), overall prediction accuracy (OA), sensitivity (SE), and specificity (SP). And the receiver operating characteristic curve (ROC) was plotted against the sensitivity of all possible cutoff points (False-positive rate) to obtain the statistical arithmetic parameter, named area under the receiver operating characteristic curve (AUC). AUC is also used as a reliable statistical parameter to assess the accuracy of model predictions. The main parameter calculation formulas used in this study are as follows:

$RMSE=\sqrt{\frac{1}{m}\sum_{i=1}^{m} (y_{i}-\mathop{y_{i}}\limits^{\wedge} )^{2}}$ （1）

$R^{2}=1-\frac{\sum_{i=1}^{m} \text{（}y_{i}-\mathop{y}\limits^{\wedge} _{i})^{2}}{\sum_{i=1}^{m} \text{（}y_{i}-\mathop{y}\limits^{-} _{i})^{2}}$ （2）

$P\text{recision}=\frac{TP}{TP+FP}\times100\%$ （3）

$F-\text{measure}=\frac{2\times P\text{recision}\times R\text{ecall}}{P\text{recision}+R\text{ecall}}\times100\%$ （4）

$SE=R\text{ecall}=\frac{TP}{TP+FN}\times100\%$ （5）

$SP=\frac{TN}{TN+FP}\times100\%$ （6）

$OA=\frac{TP+TN}{TP+TN+FP+FN}\times100\%$ （7）

$ER=(1-\frac{TP+TN}{TP+TN+FP+FN})\times100\%$ （8）

$MCC=\frac{TP\times TN-FN\times FP}{\sqrt{(TP+FN)(TP+FP)(TN+FN)(TN+FP)}}$ （9）

Among them, TP, TN, FP and FN respectively represent the true positive number, true negative number, false positive number and false negative number of the 5 compound properties predicted by the model. $y_{i}$, $\mathop{y_{i}}\limits^{\wedge}$ and $\mathop{y}\limits^{-} _{i}$ represent the real value, predicted value and average value of the sample, respectively; *m* is the number of samples in the data set. SE and SP represent the percentage of correct prediction in positive samples (major compounds) and negative samples (minor compounds), respectively. OA represents the percentage of compounds that are predicted correctly. The range of MCC is [-1, 1]. -1 and 1 respectively represent that the predicted value is completely opposite to the true value and completely correct to the true value, while 0 represents random prediction. The higher the MCC value, the better the predictive ability of the model.

$SS_{i}(h)=\sum_{i=1}^{n} (y_{ij}-\mathop{y}\limits^{\wedge} _{ij}(h))^{2}$ （10）

$PRESS_{j}(h)=\sum_{i=1}^{\text{n}} (y_{ij}-\mathop{y_{(i)j}(h)}\limits^{\wedge} )^{2}$ （11）

$Q_{h}^{2}=1-\frac{PRESS_{(h)}}{SS_{(h-1)}}$ （12）

Where, $\mathop{y}\limits^{\wedge} _{(i)j}h$represents the predicted value at the *i* observation point. PRESS*_j_h* is the square sum of the prediction errors of the *j* dependent variable *y_j_* (*j* = 1, 2, , *p*) when *h* components are extracted. $\mathop{y}\limits^{\wedge} _{ij}h$ means the predicted value of the *i* sample obtained from the regression equation fitting with *h* components. SS is the squared errors sum of $y_{j}$. Before the end of each step of modeling calculation, cross validation is carried out. If *Q_h_*^2^ < 1-0.95^2^ = 0.0985 is met in step *h*, the model meets the accuracy requirements, and the extraction of components can be stopped. When *Q_h_*^2^ ≥ 0.0975, the marginal contribution of *t_h_* component extracted in step *h* is significant, so the calculation in step *h* + 1 should be continued.

***Application domain (AD) analysis***

Since the applicability of the models to predictive compounds is limited, the AD of the models should be defined. The Leverage method is used to define the AD of the models in this study. The Leverage value (*h*) and critical value (*h*^*^) of each model were calculated. Williams diagrams were drawn with *h* and standardized residuals (SR) as abscissa and ordinate respectively. The calculation formula of *h* and *h*^*^ were as follows:

$h_{i}=x_{i}（X^{T}X）^{-1}x_{i}^{T}$ *（i=1, 2, 3......n）* （13）

$h^{*}=3(p+1)/m$ （14）

Where, *x_i_* represents the MD matrix (i.e., the eigenmatrix) of the predicted sample *i*; *X* represents the eigenmatrix of TRS sample; *X^T^* represents the transpose matrix of *X*; and *n* represents the number of the predicted samples [34]. The *p* represents the number of MD in the model; and *m* represents the number of samples in TRS. The two horizontal dashed lines represent triple SR (± 3*δ*), and the vertical dashed lines represent *h*^*^ values in the Williams diagram. The region bounded by the *Y*-axis and three dotted lines represents the AD of the model, and the region range can be expressed as *h* ≤ *h*^*^, and -3*δ* ≤ SR ≤ 3*δ*.

**Supplementary results**

Table S2 Characterization of chemical constituents in ARF by UPLC-LTQ-Orbitrap-MS^n^

| No. | *t*_R_ (min) | Expected values (m/z) | Measured  (*m/z*) | delta ppm | MS^2^ fragment ions  (*m/z*) | Identification | Molecular Formula | M |
| --- | --- | --- | --- | --- | --- | --- | --- | --- |
|
| C **1** | 1.10 | 191.01863 | 191.01906 [M-H]^-^ | 0.431 | 191.02022, 173.00967, 129.01949, 111.00903 | Citric acid | C_6_H_8_O_7_ | 192 |
| C **2** | 2.50 | 169.01315 | 169.01416 [M-H]^-^ | 1.010 | 169.01463, 125.02464 | Gallic acid | C_7_H_6_O_5_ | 170 |
| C **3** | 3.45/8.61 | 331.10236 | 331.10345 [M-H]^-^ | 1.091 | 331.06769, 313.05701, 287.07703, 169.01424, 154.12590, 154.02716, 169.05064, 287.09277, 313.07214, 331.08322 | 4-hydroxy-2,6-dimethoxyphenol-1-*O*-beta-*D*-glucopyranoside | C_14_H_20_O_9_ | 332 |
| C **4** | 4.17 | 167.03389 | 167.03473 [M-H]^-^ | 0.845 | 123.04534, 122.06926, 167.03543 | Vanillic acid | C_8_H_8_O_4_ | 168 |
| C **5** | 5.09 | 153.01824 | 153.01920 [M-H]^-^ | 0.965 | 153.01968, 109.02972, | Protocatechuic acid | C_7_H_6_O_4_ | 154 |
| C **6** | 6.00 | 211.06010 | 211.06097 [M-H]^-^ | 0.870 | 211.06186, 195.02954, 181.05130, 167.08257, 139.04013 | 3,4,5-Trimethoxy-benzoic acid | C_10_H_12_O_5_ | 212 |
| C **7** | 6.44 | 353.08671 | 353.08725 [M-H]^-^ | 0.541 | 353.08893, 179.03574, 191.05675, 173.04582, 135.04550, 180.03862 | Neochlorogenic acid | C_16_H_18_O_9_ | 354 |
| C **8** | 8.16 | 337.09179 | 337.09201 [M-H]^-^ | 0.216 | 337.09402, 173.04588, 191.05650, 163.04066, 119.05059 | 4-*O*-*p*-coumaroyl quinic acid | C_16_H_18_O_7_ | 322 |

Table S2 Characterization of chemical constituents in ARF by UPLC-LTQ-Orbitrap-MS^n^（Continued）

| No. | *t*_R_ (min) | Expected values (m/z) | Measured  (*m/z*) | delta ppm | MS^2^ fragment ions  (*m/z*) | Identification | Molecular Formula | M |
| --- | --- | --- | --- | --- | --- | --- | --- | --- |
|
| C **9** | 8.64 | 475.14462 | 475.14520 [M-H]^-^ | 1.227 | 475.14523, 437.14545, 429.14072, 413.14609, 373.11499, 331.10440, 357.11932, 343.14001, 313.09311, 293.08777, 267.08759,121.02976, 138.19635, 151.04048, | methyl salicylate gentiobioside | C_20_H_28_O_13_ | 476 |
| C **10** | 8.88 | 353.08671 | 353.08676 [M-H]^-^ | 0.051 | 353.08896, 179.03549, 191.05675, 173.04585, 135.04550, 161.02477, 180.03584, 182.03357 | chlorogenic acid | C_16_H_18_O_9_ | 354 |
| C **11** | 8.90 | 289.07066 | 289.07108 [M-H]^-^ | 0.415 | 289.07236, 271.06171, 245.08278, 205.05109, 179.03540, 109.02982 | (+)-Catechin | C_15_H_14_O_6_ | 290 |
| C **12** | 9.07 | 475.14462 | 475.14520 [M-H]^-^ | 1.227 | 443.12094, 281.06750, 151.04059, 137.02504, 323.09958, 353.08905, 331.18018, 399.13101 | methyl salicylate lactoside | C_20_H_28_O_13_ | 476 |

Table S2 Characterization of chemical constituents in ARF by UPLC-LTQ-Orbitrap-MS^n^（Continued）

| No. | *t*_R_ (min) | Expected values (*m*/*z*) | Measured  (*m/z*) | delta ppm | MS^2^ fragment ions  (*m/z*) | Identification | Molecular Formula | M |
| --- | --- | --- | --- | --- | --- | --- | --- | --- |
|
| C **13** | 9.29 | 607.18688 | 607.18604 [M-H]^-^ | 1.225 | 575.15455, 531.17316, 455.14185, 413.11011, 323.09930, 293.28865, 221.06732, 191.05672, 281.06775, 353.08914, 531.17316, 607.20825 | MSTG-B | C_25_H_36_O_17_ | 608 |
| C **14** | 10.11 | 343.24790 | 343.24744 [M+CHOO]^-^ | -0.461 | 343.14102, 325.12985, 299.05859, 296.97104, 281.10355 | 9-Octadecenic acid | C_18_H_34_O_3_ | 298 |
| C **15** | 10.25 | 445.13405 | 445.13495 [M-H]^-^ | 2.016 | 445.13635, 413.11035, 353.08917, 191.05663, 151.04071, 137.02509 | methyl salicylate vicianoside | C_19_H_26_O_12_ | 446 |
| C **16** | 10.29 | 445.13405 | 445.13470 [M-H]^-^ | 1.455 | 445.13635, 353.08917, 413.11035, 299.07843, 137.02509, 151.04071 | Gaultherin | C_19_H_26_O_12_ | 446 |
| C **17** | 10.39 | 385.18569 | 385.18582 [M-H]^-^ | 0.326 | 223.13501, 205.12428, 153.09283, 161.04619,179.06656, 151.07697 | Roseoside | C19H30O8 | 386 |
| C **18** | 10.62 | 577.17631 | 577.17688 [M-H]^-^ | 0.568 | 623.18359, 577.18024, 545.15448, 463.23419, 293.08887, 445.13873, 413.10333 | MSTG-A | C_24_H_34_O_16_ | 578 |

Table S2 Characterization of chemical constituents in ARF by UPLC-LTQ-Orbitrap-MS^n^（Continued）

| No. | *t*_R_ (min) | Expected values (*m*/*z*) | Measured  (*m/z*) | delta ppm | MS^2^ fragment ions  (*m/z*) | Identification | Molecular Formula | M |
| --- | --- | --- | --- | --- | --- | --- | --- | --- |
|
| C **19** | 10.89 | 337.09179 | 337.09268 [M-H]^-^ | 0.886 | 191.05672, 163.04056, 173.04585, 119.05048 | 5-*O*-*p*-coumaroyl quinic acid | C_16_H_18_O_8_ | 338 |
| C **20** | 10.99 | 289.07066 | 289.07156 [M-H]^-^ | 0.895 | 289.07260, 271.06192, 205.05118, 245.08298, 179.03546, 109.02985 | (-)-Catechin | C_15_H_14_O_6_ | 290 |
| C **21** | 11.24 | 359.09727 | 359.09787 [M-H]^-^ | 1.662 | 359.11279, 313.09354, 281.06711, 197.04561, 151.04301, 137.02455 | Methyl salicylate-*β*-glucoside | C_14_H_18_O8 | 314 |
| C **22** | 11.29 | 347.07614 | 347.07605 [M-H]^-^ | -0.270 | 347.07861, 329.06793, 303.08826, 285.07730, 259.09842, | (+)-Homoeriodictyol | C_16_H_14_O_6_ | 302 |
| C **23** | 11.35 | 347.07614 | 347.07614 [M-H]^-^ | -0.011 | 347.07712, 301.09296, 329.06689, 303.08740, 285.07678，163.04030，153.05585，149.02461，177.05598，135.04555，117.19879, 151.04028, | Hesperetin | C_16_H_14_O_6_ | 302 |
| C **24** | 11.39 | 419.17004 | 419.16672 [M-H]^-^ | 2.965 | 419.17221, 404.14923, 373.12970, 389.12457 | (+)-Lyoniresinol | C_22_H_28_O_8_ | 420 |
| C **25** | 12.09 | 209.04445 | 209.04495 [M-H]^-^ | 0.500 | 180.98932, 209.04620, 165.05614, 121.02977 | *p*-Coumaric acid | C_9_H8O_3_ | 164 |

Table S2 Characterization of chemical constituents in ARF by UPLC-LTQ-Orbitrap-MS^n^（Continued）

| No. | *t*_R_ (min) | Expected values (*m*/*z*) | Measured  (*m/z*) | delta ppm | MS^2^ fragment ions  (*m/z*) | Identification | Molecular Formula | M |
| --- | --- | --- | --- | --- | --- | --- | --- | --- |
|
| C **26** | 12.67 | 581.22287 | 581.22388 [M-H]^-^ | 1.742 | 581.22668, 419.17340, 566.20258, 404.14957, 386.13843, 355.12003, 373.13068 | (+)-Lyoniresinol-2-*α*-*O*-*β*-D-glucopyranoside | C_28_H_38_O_13_ | 582 |
| C **27** | 13.87 | 551.21230 | 551.21246 [M-H]^-^ | 0.285 | 551.21588, 419.17328, 536.19214, 505.26678, 401.16263, 386.13831 | (+)-Lyoniresinol-2*α*-*O*-*β*-l-arabinopyranoside | C_27_H_36_O_12_ | 552 |
| C **28** | 14.06/16.96/17.75 | 491.19117 | 491.19019 [M-H]^-^ | -2.002 | 491.17953, 473.16885, 429.17880, 359.15149, 315.14627, 267.07339, 249.06279 | (-)-Isolariciresinol-2*α*-*O*-*β*-d-xylopyranoside | C_25_H_32_O_10_ | 492 |
| C **29** | 14.16 | 521.20174 | 521.20056 [M-H]^-^ | -2.261 | 521.20567, 506.18051, 359.15204, 341.14078 | (-)-5'-Methoxyisolariciresinol-2*α*-*O*-*β*-d-xylopyranoside | C_26_H_34_O_11_ | 522 |
| C **30** | 14.32 | 477.06367 | 477.06683 [M-H]^-^ | 0.971 | 431.22992, 301.03641, 331.06863, 179.29710 | quercetin-3-*O*-glucuronide | C_21_H_18_O_13_ | 478 |
| C **31** | 14.51 | 463.08710 | 463.08612 [M-H]^-^ | -2.121 | 463.09021, 301.03653, 417.25125, 331.17731, 178.99916, 151.00421 | hyperoside | C_21_H_20_O_12_ | 464 |

Table S2 Characterization of chemical constituents in ARF by UPLC-LTQ-Orbitrap-MS^n^（Continued）

| No. | *t*_R_ (min) | Expected values (*m*/*z*) | Measured  (*m/z*) | delta ppm | MS^2^ fragment ions  (*m/z*) | Identification | Molecular Formula | M |
| --- | --- | --- | --- | --- | --- | --- | --- | --- |
|
| C **32** | 14.73 | 301.03428 | 301.03397[M-H]^-^ | -1.027 | 301.03622, 273.04108, 229.05130, 193.01474, 178.99945, 151.00438, 121.02998, 301.03641, 273.04108, 257.04611, 229.05112, 193.01468, 178.99939, 151.00432, 121.02995 | Quercetin | C_15_H_10_O_7_ | 302 |
| C **33** | 14.78 | 479.15479 | 479.15378 [M-H]^-^ | -2.104 | 525.19928, 507.18961, 479.07559, 327.10938 | Paeoniflorin | C_23_H_28_O_11_ | 480 |
| C **34** | 15.35 | 137.02332 | 137.02393 [M-H]^-^ | 4.448 | 136.86319, 93.03454 | 4-hydroxybenzoic acid | C_7_H_6_O_3_ | 138 |
| C **35** | 15.67 | 433.07654 | 433.07559 [M-H]^-^ | -2.188 | 433.07834, 415.06693, 373.05664, 300.02844, 301.03610, 178.99876, 151.00394 | Avicularin | C_20_H_18_O_11_ | 434 |
| C **36** | 15.71 | 145.08592 | 145.08659 [M-H]^-^ | 4.612 | 145.08736, 99.08159, 127.07652, 104.03097 | Hexanal/3-Hexen-1-ol | C6H12O | 100 |
| C **37** | 16.01 | 461.07145 | 461.07108 [M-H]^-^ | -0.807 | 285.04092, 153.01994, 179.03601 | Kaempferol-3-*O*-*β*-d-glucuronide | C_21_H_18_O_12_ | 462 |
| C **38** | 16.05 | 291.08631 | 291.08618 [M-H]^-^ | -0.463 | 291.08643, 273.01583, 247.09706, 245.08250, 227.07155, 201.09239, 203.07170 | Fraxinellonone | C_14_H_14_O_4_ | 246 |

Table S2 Characterization of chemical constituents in ARF by UPLC-LTQ-Orbitrap-MS^n^（Continued）

| No. | *t*_R_ (min) | Expected values (*m*/*z*) | Measured  (*m/z*) | delta ppm | MS^2^ fragment ions  (*m/z*) | Identification | Molecular Formula | M |
| --- | --- | --- | --- | --- | --- | --- | --- | --- |
|
| C **39** | 16.46 | 447.09219 | 447.09204 [M-H]^-^ | -0.331 | 447.09518, 301.03638, 429.08362, 373.05682, 343.04642, 321.06192, 285.04221, 255.03050, 178.99910, 151.00412 | Quercitrin | C_21_H_20_O_11_ | 448 |
| C **40** | 17.45 | 317.08671 | 317.08670 [M-H]^-^ | -0.009 | 109.02970, 151.00378, 178.99883, 271.02469, 317.03073 | Catechol-*β*-d-glucopyranoside /Arbutin | C_12_H_16_O_7_ | 272 |
| C **41** | 17.50 | 317.02919 | 317.02927 [M-H]^-^ | 0.241 | 317.03052, 178.99881, 151.00380, 137.02458 | Myricetin | C_15_H_10_O_8_ | 318 |
| C **42** | 17.77 | 419.17004 | 419.16672 [M-H]^-^ | 2.965 | 419.17221, 404.14923, 373.12970, 389.12457, 297.06158, 375.04999, 315.05298, | (+)-Lyoniresinol | C_22_H_28_O_8_ | 420 |
| C **43** | 17.88 | 473.18061 | 473.17972 [M-H]^-^ | -1.879 | 473.16830, 429.17831, 413.14749, 369.15576 | Gaultherin A | C_25_H_30_O_9_ | 474 |
| C **44** | 23.17 | 285.03936 | 285.03992 [M-H]^-^ | 1.949 | 285.04092, 257.04556, 229.05063, 213.05573, 151.00377, 121.23774, 285.04083, 153.01994, 178.99236, 151.00372, 244.98427 | Kaempferol | C_15_H_10_O_6_ | 286 |

Table S2 Characterization of chemical constituents in ARF by UPLC-LTQ-Orbitrap-MS^n^（Continued）

| No. | *t*_R_ (min) | Expected values (*m*/*z*) | Measured  (*m/z*) | delta ppm | MS^2^ fragment ions  (*m/z*) | Identification | Molecular Formula | M |
| --- | --- | --- | --- | --- | --- | --- | --- | --- |
|
| C **45** | 23.53 | 401.15948 | 401.15924 [M-H]^-^ | -0.597 | 401.08911, 357.06311, 313.07312, 225.05623, 121.13014 | Gaultherin C | C_22_H_26_O_7_ | 402 |
| C **46** | 24.18 | 207.10157 | 207.10214 [M-H]^-^ | 2.748 | 207.10307, 179.10796 | Elemicine | C_12_H_16_O_3_ | 208 |
| C **47** | 25.26 | 195.13796 | 195.13881 [M-H]^-^ | 4.374 | 195.13962, 167.14441, 151.03993 | Bornyl acetate/Geranyl acetate | C_12_H_20_O_2_ | 196 |
| C **48** | 30.55 | 165.05462 | 165.05525 [M-H]^-^ | 3.813 | 165.04126, 149.00974, 146.96861, 122.03593 | 2-Hydroxy-4-methoxyacetophenone | C_9_H_10_O_3_ | 166 |

**Establishment of PGPI prediction model**

***PGPI data distribution and feature selection***

PCA was conducted according to whether a compound was PGPI or not. The results indicate that the two classes of compounds showed an overall trend of separation but not significant, with the cumulative contribution rate of 26 principal components reaching 80% and that of 52 principal components reaching 90%. As shown in Figure S1A, the distribution of TRS, TES and VAS data subset of compound PGPI property are exhibited in the 3D projection diagram, a scatter diagram that the top three PC factors three-dimensional space. Although the contribution rate of descending order by a 3D chart the clustering phenomenon is not obvious, the distribution of sample points in three data sets are evenly distributed in the whole space, indicate that the selected data sets in this study are feasible. The specimen in TRS cover the whole range of PGPI, illustrates the specimens in TRS have good representativeness. The principal component contribution rate of the top ten of principal components (PC) is depicted in Figure S2A. Among them, the variance contribution rate of the first principal component (PC_1_), the second principal component (PC_2_), the third principal component (PC_3_) and the fourth principal component (PC_4_) is 27.28%, 8.99%, 6.45% and 5.49%, respectively. Since PC_5_, the variance contribution rate is less than 5%. The cumulative variance contribution rate of the first 26 PCs reached 80.14%, indicating that the first 26 principal components could reflect the information of 80.14% of the original data in the experiment.

**
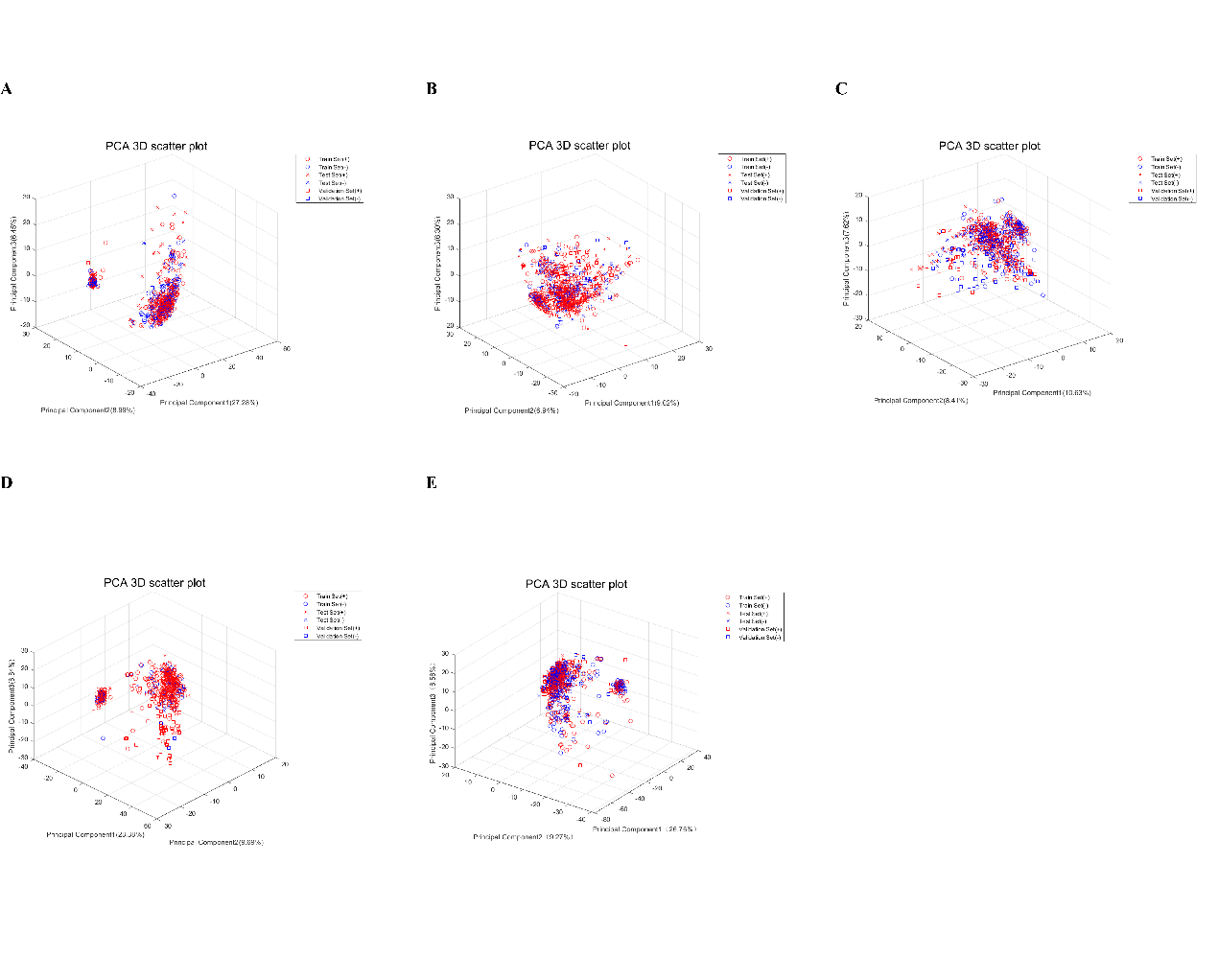
**

**Figure S1 3D score plot of 5 ADME properties data set.** A: PGPI, B: PGPS, C: Caco 2, D: HIA, E: OB. Molecule distribution for the molecules selected for this study in the three datasets.


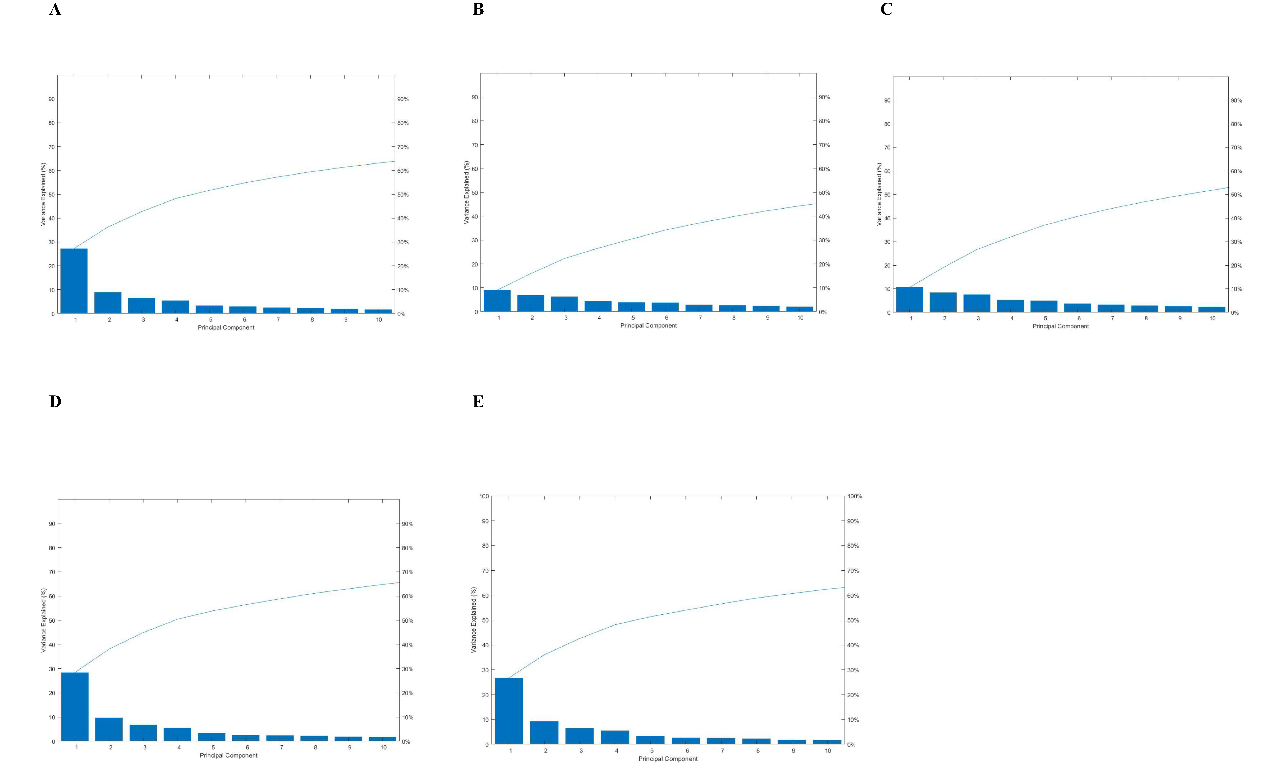


**Figure S2 Principal component contribution rate of the top ten principal components of MDs of 5 ADME properties.** A: PGPI, B: PGPS, C: Caco 2, D: HIA, E: OB.

***Selection and optimization of PGPI prediction models***

In total, there are six types of machine learning methods. MLs and resampling methods were used simultaneously for ameliorating the reliability and robustness of the models. Since there are still huge amounts of data after PCA processing, which leads to a long running time, the data with the cumulative contribution rate of principal components of 80% was selected to carry out the next calculation. GA was applied to screen PC factors. Then, EBPT, RF, SVM, KNN and PLS-DA algorithm were respectively operated separately or combined with GA which already run optimization operation for many times. AUC, Precision, Recall and F-measure were used to evaluate the model. OA was used as the index to simply evaluate the classification effect on the prediction model building by the different data sets and ways of using classifiers. The fitness function, the prediction accuracy of the five-point cross validation of the model, were adopted to evaluate the impact of GA on single-classifier modeling [93]. For the PLS-DA algorithm, LOO test was applied to evaluate the predictive ability of PLS-DA model and PLS-DA+GA model with AUC, RMSE, R^2^ and cross validity parameters (*Q_h_*^2^).

As shown in Table S3 and Table S4, compared with those models optimized by GA, the 6 static parameters of PGPI-EBPT, PGPI-RF and PGPI-KNN have not been improved, which indicates that GA has no promoting effect on the prediction ability of the three prediction models of PGPI properties. The Precision and Recall value of PGPI-EBPT model are 96.61% and 98.95%, while that of PGPI-EBPT+GA model were 98.06% and 64.78%, respectively. The change trend of Precision and Recall values of the two models is opposite. At this time, F-measure index, as an evaluation index integrating recall and precision, was payed more attention to be applied in comparing with two models. The F-measure value of PGPI-EBPT model is 98.04%, which higher than that of PGPI-EBPT+GA model (76.83%). The results indicate that the performance of PGPI-EBPT model is better than PGPI-EBPT+GA model. The OA of PGPI-SVM+GA and PGPI-PLS-DA+GA models was 83.81% and 92.09%, respectively. They are higher than the OA value of PGPI-SVM and PGPI-PLS-DA models. The results imply that for SVM and PLS-DA algorithms, the prediction ability of the models established by these two algorithms has been improved by using GA optimization method. PLS-DA even shows more noticeable predominance as indicated by those statistical evaluations when combined with GA.

Table S3 Statistic evaluations, ER, Precision, Recall, F-measure, AUC and OA evaluated by 4 single classifiers alone or combined with GA in the test set of PGPI

| Model | ER (%) | Precision (%) | Recall (%) | F-measure (%) | AUC | OA (%) |
| --- | --- | --- | --- | --- | --- | --- |
| PGPI-EBPT | 13.28 | 96.61 | 98.95 | 98.04 | 0.5648 | 86.72 |
| PGPI-EBPT+GA | 22.50 | 98.06 | 64.78 | 76.83 | 0.6290 | 77.50 |
| PGPI-SVM | 19.81 | 84.78 | 60.94 | 70.91 | 0.8377 | 80.19 |
| PGPI-SVM+GA | 16.19 | 84.27 | 66.96 | 74.63 | 0.5870 | 83.81 |
| PGPI-RF | 14.01 | 84.27 | 71.43 | 77.32 | 0.6664 | 85.99 |
| PGPI-RF+GA | 16.46 | 78.89 | 68.27 | 73.20 | 0.6215 | 83.54 |
| PGPI-KNN | 9.25 | 79.81 | 73.45 | 76.50 | 0.9103 | 90.75 |
| PGPI-KNN+GA | 10.91 | 69.15 | 61.90 | 65.33 | 0.8944 | 89.09 |

Table S4 Statistic evaluations, *R*^2^, *Q_h_*^2^, RMSE, AUC and OA evaluated by PLS-DA alone or combined with GA in the test set of 5 ADME properties

| Model | *R*^2^ | *Q_h_*^2^ | RMSE | AUC | OA (%) |
| --- | --- | --- | --- | --- | --- |
| PGPI-PLS-DA | 0.4474 | 0.0234 | 0.3360 | 0.6602 | 89.97 |
| PGPI-PLS-DA+GA | 0.3632 | 0.0082 | 0.2824 | 0.7524 | 92.09 |
| PGPS-PLS-DA | 0.3567 | 0.0823 | 0.2726 | 0.9959 | 90.57 |
| PGPS-GA+PLS-DA | 0.2020 | 0.0418 | 0.3254 | 0.9958 | 93.96 |
| Caco 2-PLS-DA | 0.3926 | 0.0384 | 0.3368 | 0.9935 | 89.58 |
| Caco 2-PLS-DA+GA | 0.0271 | 0.0025 | 0.0874 | 0.9934 | 97.21 |
| HIA-PLS-DA | 0.5918 | 0.0714 | 0.2140 | 0.9140 | 96.63 |
| HIA-PLS-DA+GA | 0.5820 | 0.0772 | 0.2304 | 0.8974 | 95.93 |
| OB-PLS-DA | 0.3075 | 0.0182 | 0.3714 | 0.9931 | 91.84 |
| OB-PLS-DA+GA | 0.1935 | 0.0000 | 0.3072 | 0.9932 | 93.42 |

***Evaluation of the PGPI prediction models***

After finishing all above work, ten different MLs (SVM, PLS-DA, KNN, EBPT, RF, GA-SVM, GA-PLS-DA, GA-RF, GA-KNN and GA-EBPT) are applied on the dataset and their performances are compared with detailed statistics summarized in Table S5. MCC, SE, SP, OA and ER were used as indicators to evaluate the predictive performance of the model on TRS, TES and VAS respectively. The predictive ability of each single classification is inferior to that established by the corresponding model combined with GA. Except for the PGPI-EBPT and PGPI-EBPT+GA models, the other models have good predictive performance at discrimination PGPI. The prediction accuracy of TRS, TES and VAS data sets in those models were all over 78.71%. For PLS-DA and EBPT algorithm, the models optimized by GA have better forecast ability. While for SVM, RF and KNN algorithm, the method of feature selected based on GA is not enough conducive to improve the OA and robustness of the models.

***AD analysis of the PGPI prediction models***

The purpose of determining the AD of the model is to test the predictive reliability of the model to the sample. The leverage method described in the literature was used to define the AD of the model [94]. The AD of the PGPI property prediction models obtained in this study were characterized by Figure S3. For the PGPI-EBPT model and PGPI-EBPT+GA model, the standardized residual values of compounds in the training set and the validation set are in a range of −8 ~ 8 (Figure S3A ~ S3B), implying that there is no response outlier. While for the other 8 models, the standardized residual values of compounds in two datasets are in a range of −6 ~ 6 (Figure S3C ~ S3J). It can be seen that all the samples in TRS and VAS of the five models of PGPI, namely PGPI-PLS-DA, PGPI-SVM, PGPI-RF, PGPI-EBPT and PGPI-KNN, fall within the effective range of applicability (0 ≤ *h* ≤ 0.0952). GA+EBPT, GA+SVM, GA+PLS-DA, GA+RF and GA+KNN model samples fall within the effective range of 0 ≤ *h* ≤ 0.0493, 0 ≤ *h* ≤ 0.0317, 0 ≤ *h* ≤ 0.0529, 0 ≤ *h* ≤ 0.0670, and 0 ≤ *h* ≤ 0.0952, respectively. In addition, within the range of AD, no statistical outliers appeared in the TRS and VAS of the 10 models of PGPI, indicating that they all had good fitting and prediction abilities. There were no outliers outside the effective range of AD, indicating that the model was of good generality. To sum up, the prediction models of compound PGPI property established in this study are all effective, statistically correct and reliable models.


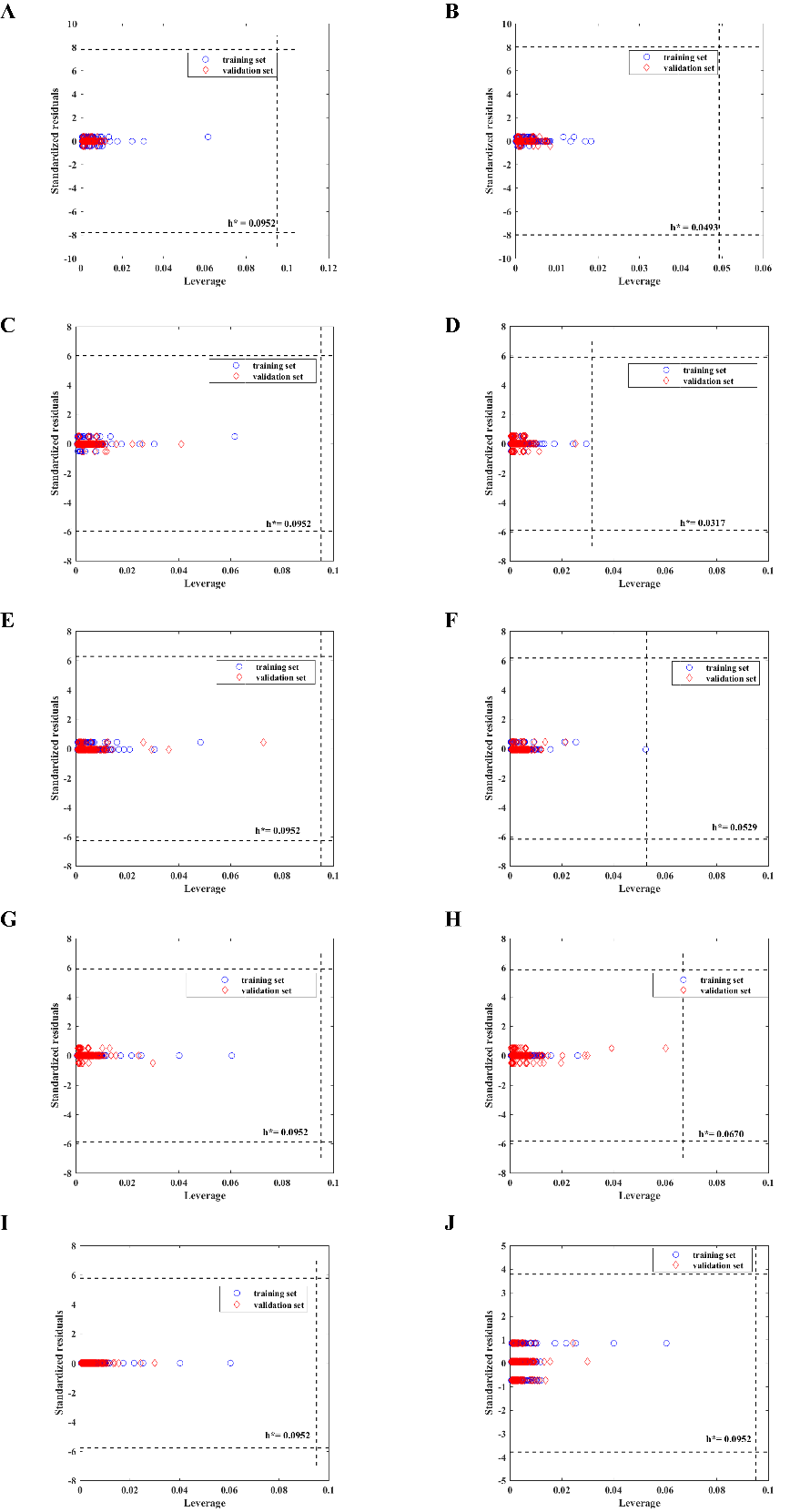


**Figure S3 Williams plots of standardized residuals versus leverage values (*h*) for the PGPI discrimination models.** (*h*^*^ is the warning leverage value.) A: Model PGPI-EBPT, B: Model PGPI-EBPT +GA, C: Model PGPI-SVM, D: Model PGPI-SVM+GA, E: Model PGPI-PLS-DA, F: Model PGPI-PLS-DA+GA, G: Model PGPI-RF, H: Model PGPI-RF+GA, I: Model PGPI-KNN, J: Model PGPI-KNN+GA.

Table S5 Performance evaluation of each prediction model of PGPI

| Model | Data Set | OA (%) | SP (%) | SE (%) | MCC | ER (%) |
| --- | --- | --- | --- | --- | --- | --- |
| PGPI-PLS-DA | TRS | 88.37 | 100.00 | 67.75 | 0.7571 | 11.63 |
|  | TES | 86.45 | 100.00 | 58.00 | 0.6952 | 13.55 |
|  | VAS | 90.97 | 100.00 | 73.58 | 0.8044 | 9.03 |
| PGPI-PLS-DA+GA | TRS | 89.42 | 100.00 | 72.81 | 0.7878 | 10.58 |
|  | TES | 92.02 | 100.00 | 75.93 | 0.8236 | 7.98 |
|  | VAS | 92.02 | 100.00 | 74.51 | 0.8171 | 7.98 |
| PGPI-SVM | TRS | 89.07 | 96.30 | 74.65 | 0.7502 | 10.93 |
|  | TES | 93.75 | 97.96 | 87.10 | 0.8688 | 6.25 |
|  | VAS | 93.13 | 97.25 | 84.31 | 0.8398 | 6.88 |
| PGPI-SVM+GA | TRS | 94.24 | 97.40 | 88.78 | 0.8755 | 5.76 |
|  | TES | 83.97 | 92.23 | 67.92 | 0.6332 | 16.03 |
|  | VAS | 78.71 | 86.41 | 63.46 | 0.5123 | 21.29 |
| PGPI-RF | TRS | 100.00 | 100.00 | 100.00 | 1.0000 | 0.00 |
|  | TES | 85.35 | 94.12 | 69.09 | 0.6714 | 14.65 |
|  | VAS | 86.62 | 92.52 | 74.00 | 0.6854 | 13.38 |
| PGPI-RF+GA | TRS | 100.00 | 100.00 | 100.00 | 1.0000 | 0.00 |
|  | TES | 85.44 | 92.08 | 73.68 | 0.6790 | 14.56 |
|  | VAS | 81.65 | 90.09 | 61.70 | 0.5445 | 18.35 |
| PGPI-EBPT | TRS | 57.87 | 0.00 | 92.20 | -0.1747 | 42.13 |
|  | TES | 69.57 | 72.41 | 66.67 | 0.3915 | 30.43 |
|  | VAS | 64.91 | 26.09 | 91.18 | 0.2323 | 35.09 |
| PGPI-EBPT+GA | TRS | 58.14 | 0.00 | 63.56 | -0.2159 | 41.86 |
|  | TES | 80.87 | 77.78 | 83.61 | 0.6155 | 19.13 |
|  | VAS | 78.95 | 96.43 | 62.07 | 0.6204 | 21.05 |
| PGPI-KNN | TRS | 90.75 | 83.81 | 97.29 | 0.8211 | 9.25 |
|  | TES | 100.00 | 100.00 | 100.00 | 1.0000 | 0.00 |
|  | VAS | 99.32 | 0.00 | 100.00 | 1.0000 | 0.68 |
| PGPI-KNN+GA | TRS | 89.09 | 80.94 | 96.78 | 0.7899 | 10.91 |
|  | TES | 100.00 | 100.00 | 100.00 | 1.0000 | 0.00 |
|  | VAS | 99.39 | 0.00 | 100.00 | 1.0000 | 0.61 |

**Establishment of PGPS prediction model**

***PGPS data distribution and feature selection***

To investigate the classification effect and data distribution of PGPS (+) and PGPS (-), PCA was applied for their TRS, TES, VAS data sets, respectively. The 3D score plot is shown in Figure S1B. The overlapping area of TRS, TES, VAS between two types compounds are larger indicates that overall classification effect are poorer. The sample of TRS, TES and VAS can be evenly distributed in this 3D space, and the sample of TRS can cover the whole range of PGPS. It suggests that the data set division used in this study and the representativeness of each sample of TRS are reasonable and good. After eliminating descriptors with high correlation and missing values, it provided the first 52 important variables by PCA for the following operation, named PCA-80, which can explain 80% of the data set variable information in total. Figure S2B shows that the top ten principal components (PCs) in the contribution rate. The variance contribution rate of the top three PC was 9.02%, 6.94% and 6.31%, respectively, and the others are less than 5%. Principal component factor 1 ~ 52 can be pitched as important features for further analysis.

***Construction and optimization of PGPS prediction models***

The method of model established and optimization on PGPS were the same as that of PGPI. The advantages and disadvantages of single classification with or without GA models based on PCA-80 modeling data of PGPS were preliminarily screened. In addition to PGPS-PLS-DA model and PGPS-PLS-DA+GA model (Table S4), the comprehensive evaluation was carried out with 6 statistical parameters for the remaining models, such as AUC, ER, OA, Precision, Recall and F-measure (Table S6). For the PGPS properties of compounds, except SVM, the accuracy of the models established by the other four algorithms combined with GA have not been improved compared with the models obtained by single classifications. The overall OA of the PGPS-SVM and PGPS-SVM+GA two models ranged from 94.86% to 98.87%, with AUC values of 0.9960. The Precision, Recall and F-measure of PGPS-SVM+GA model were 100.00%, 97.81% and 98.89% respectively, which are slightly higher than those of SVM model, indicating that SVM+GA model had better precision and recall. The results showed that the classification model established by using GA+SVM has higher AUC and OA value than SVM algorithm alone. There is no significant difference between the single classifier model and ensemble model. The detail statistic evaluation parameters of 10 PGPS prediction models were listed in Table S4 and Table S6.

Table S6 Statistic evaluations, ER, Precision, Recall, F-measure, AUC and OA evaluated by 4 single classifiers alone or combined with GA in the test set of PGPS

| Model | ER (%) | Precision (%) | Recall (%) | F-measure (%) | AUC | OA (%) |
| --- | --- | --- | --- | --- | --- | --- |
| PGPS-EBPT | 2.13 | 96.61 | 98.95 | 98.04 | 0.9856 | 97.87 |
| PGPS-EBPT+GA | 10.22 | 97.84 | 99.74 | 98.95 | 0.9859 | 89.78 |
| PGPS-SVM | 5.14 | 100.00 | 90.55 | 95.04 | 0.9960 | 94.86 |
| PGPS-SVM+GA | 1.23 | 100.00 | 97.81 | 98.89 | 0.9960 | 98.87 |
| PGPS-RF | 0.58 | 99.64 | 99.29 | 99.47 | 0.9958 | 99.42 |
| PGPS-RF+GA | 0.59 | 98.86 | 100.00 | 99.43 | 0.9960 | 99.41 |
| PGPS-KNN | 9.59 | 89.39 | 98.99 | 93.95 | 0.9975 | 90.41 |
| PGPS-KNN+GA | 12.94 | 88.66 | 96.27 | 92.31 | 0.9968 | 87.06 |

***Evaluation of the PGPS prediction models***

In this study, 10 prediction models for PGPS property of compounds were evaluated and analyzed. To this end we tested the models on ability of PGPS character recognition and investigated the performance of single classifier models in comparison to a number of alternative combination strategies. Compared with PGPS-EBPT and PGPS-EBPT+GA models, the other models have better prediction ability for the PGPS property. The accuracy of TRS, TES and VAS predicted by the eight models are all above 81.24%. The prediction performance of PGPS-PLS-DA model and PGPS-KNN model both are slightly better than that of their models combined with GA, while the prediction performance of PGPS-EBPT model, PGPS-SVM model and PGPS-RF model are not as good as that of corresponding model constructed by combining with GA. Among them, the prediction performance of PGPS-RF+GA model and PGPS-RF+GA model are prominent. To a certain extent, this algorithm combined GA with RF, called GA+RF, which realizes the combination of GA's global search capability and RF algorithm's important features selection performance for PGPS. On the whole, the results demonstrate that significant gains hadn’t be obtained by integrating genetic algorithm into the classifier systems. The performance evaluation index results of each model are shown in Table S7.

Table S7 Prediction performance evaluation of each model of PGPS

| Model | Data Set | OA (%) | SP (%) | SE (%) | MCC | ER (%) |
| --- | --- | --- | --- | --- | --- | --- |
| PGPS-PLS-DA | TRS | 91.68 | 100.00 | 84.88 | 0.8464 | 8.32 |
|  | TES | 92.78 | 100.00 | 85.61 | 0.8647 | 7.22 |
|  | VAS | 92.37 | 100.00 | 86.58 | 0.8577 | 7.63 |
| PDPS-PLS-DA+GA | TRS | 91.90 | 100.00 | 84.51 | 0.8499 | 8.10 |
|  | TES | 91.32 | 100.00 | 84.03 | 0.8403 | 8.68 |
|  | VAS | 87.50 | 100.00 | 77.55 | 0.7778 | 12.50 |
| PGPS-SVM | TRS | 98.22 | 100.00 | 96.70 | 0.9650 | 1.78 |
|  | TES | 97.08 | 100.00 | 94.97 | 0.9423 | 2.92 |
|  | VAS | 98.91 | 100.00 | 97.84 | 0.9783 | 1.09 |
| PGPS-SVM+GA | TRS | 100.00 | 100.00 | 100.00 | 1.0000 | 0.00 |
|  | TES | 99.61 | 100.00 | 99.24 | 0.9923 | 0.39 |
|  | VAS | 96.90 | 100.00 | 94.16 | 0.9398 | 3.10 |
| PGPS-RF | TRS | 100.00 | 100.00 | 100.00 | 1.0000 | 0.00 |
|  | TES | 98.84 | 99.19 | 98.53 | 0.9768 | 1.16 |
|  | VAS | 100.00 | 100.00 | 100.00 | 1.0000 | 0.00 |
| PGPS-RF+GA | TRS | 100.00 | 100.00 | 100.00 | 1.0000 | 0.00 |
|  | TES | 99.61 | 99.21 | 100.00 | 0.9922 | 0.39 |
|  | VAS | 99.21 | 98.33 | 100.00 | 0.9843 | 0.79 |
| PGPS-EBPT | TRS | 21.28 | 0.00 | 21.84 | -0.2899 | 78.72 |
|  | TES | 88.57 | 82.26 | 93.59 | 0.7690 | 11.43 |
|  | VAS | 54.29 | 96.97 | 16.22 | 0.2194 | 45.71 |
| PGPS-EBPT+GA | TRS | 2.46 | 0.00 | 2.50 | -0.6143 | 97.54 |
|  | TES | 87.86 | 82.81 | 92.11 | 0.7558 | 12.14 |
|  | VAS | 51.43 | 100.00 | 2.86 | 0.1204 | 48.57 |
| PGPS-KNN | TRS | 90.41 | 82.53 | 97.11 | 0.8119 | 9.59 |
|  | TES | 85.52 | 0.00 | 98.21 | -0.0485 | 14.48 |
|  | VAS | 87.61 | 0.00 | 98.45 | -0.0415 | 12.39 |
| PGPS-KNN+GA | TRS | 87.06 | 77.28 | 95.39 | 0.7461 | 12.94 |
|  | TES | 81.24 | 0.00 | 99.19 | -0.0384 | 18.76 |
|  | VAS | 82.17 | 0.00 | 98.44 | -0.0511 | 17.83 |

***AD analysis of the PGPS prediction models***

The AD of the compound PGPS property prediction models obtained in this research are depicted in Figure S4. For the PGPS-EBPT model and PGPS-EBPT+GA model, as characterized by Figure S4A and Figure S4B, the SR values of both compounds in the TRS and the VAS are in a range of −7 ~ 7, implying that there is no response outlier. The outlier wasn’t observed in the other 8 models (Figure S4C ~ S4J). The results showed that the *h* * values of 5 single classifiers model of compound PGPS all were 0.1130. The *h* * values of model PGPS-EBPT+GA, PGPS-SVM+GA, PGPS-PLS-DA+GA, PGPS-RF+GA and PGPS-KNN+GA were 0.0618, 0.0405, 0.0661, 0.0853 and 0.1130, respectively. Most of the samples of TRS and VAS of ten models fall within the scope of valid applicability, but there are different numbers of X exceptional points. Except for PGPS-RF+GA model, the number of X exception points in TRS and VAS of other 9 models was no more than 2, exhibits that all the compounds are within these models applicability domain. To sum up, the samples of training set and validation set of most of those models were within the range of AD, which illustrated that the prediction models established in this study had excellent validity for sample prediction, good model fitting ability and prediction ability.


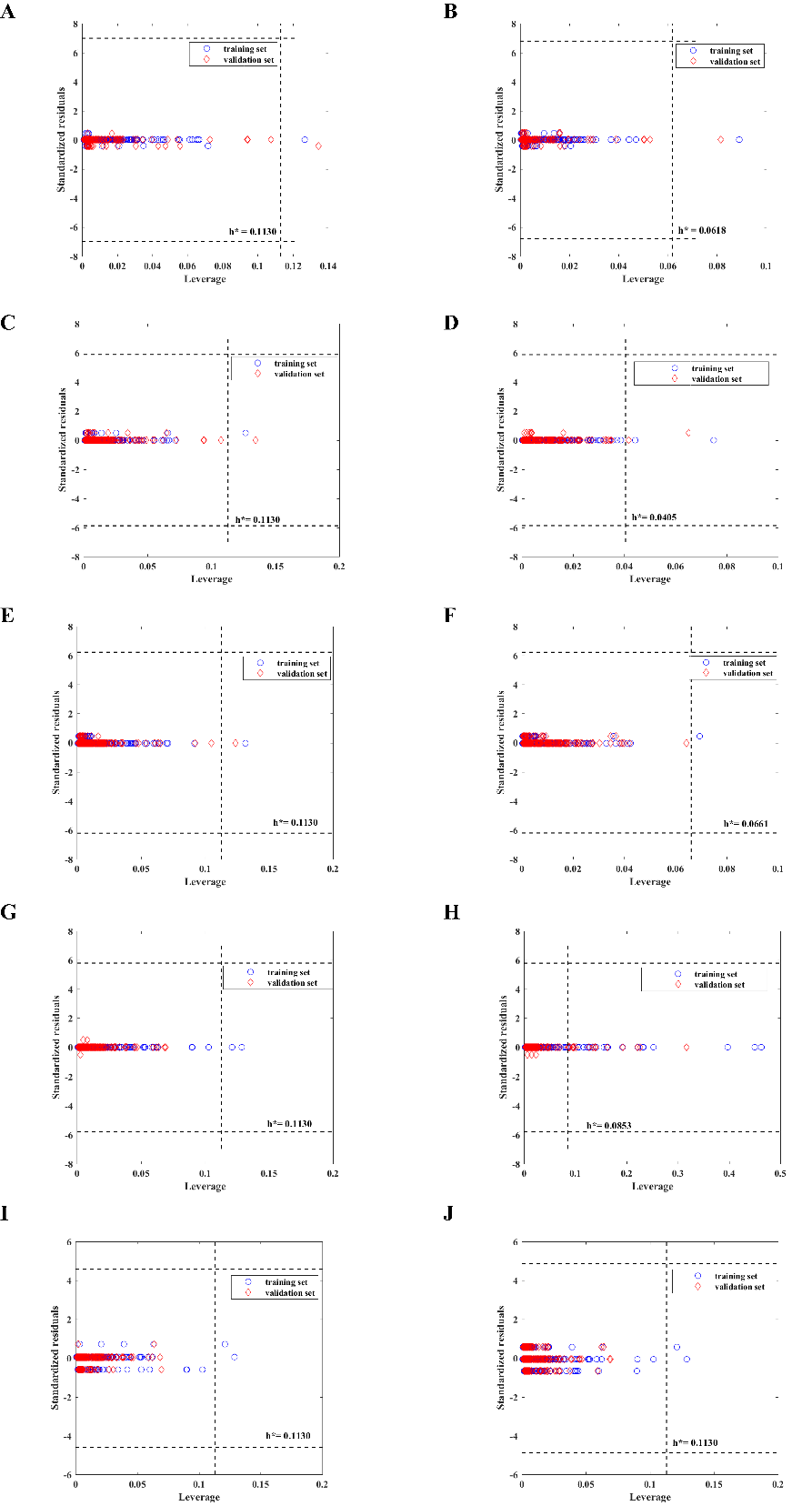


**Figure S4 Williams plots of standardized residuals versus leverage values (*h*) for the PGPS discrimination models.** (*h*^*^ is the warning leverage value.) A: Model PGPS-EBPT, B: Model PGPS-EBPT+GA, C: Model PGPS-SVM, D: Model PGPS-SVM+GA, E: Model PGPS-PLS-DA, F: Model PGPS-PLS-DA+GA, G: Model PGPS-RF, H: Model PGPS-RF+GA, I: Model PGPS-KNN, J: Model PGPS-KNN+GA.

**Establishment of Caco 2 prediction model**

***Caco 2 permeability data distribution and feature selection***

PCA was applied to the pretreated Caco 2 data, which was carried out for two kinds of compounds, called Caco 2 (+) and Caco 2 (+). The results showed that the separation trend of the two kinds of compounds is not obvious (Figure S1C). The histogram of top ten PCs variance contribution rate is shown in Figure S2C. The variance contribution rate of the first PC is 10.63%. The variance contribution rate of the second, third and fourth PC is 8.41%, 7.62% and 5.30% respectively, while the variance contribution rate of the rest PC is less than 5%. The sum of variance contribution rate of the first 34 principal components is 80.26%, which indicates that these 34 PCs can be used to represent 80.26% of the original data in the experiment. They are collectively referred to as PCA-80. According to the 3D score chart of Figure 6C, the clustering phenomenon of samples in TRS, TES and VAS all were not significant, which depicts the configuration of the samples on the first three PCs, we can observe that the four groups of diesel samples are not clearly identified. The samples of three data subsets are evenly distributed in the whole space, which reckons that the data set used in this study is divided reasonably, and that each sample in TRS is well represented. Finally, the 34 principal components were selected for further analysis and used as modeling data.

***Construction and optimization of Caco 2 prediction models***

The establishment and preliminary evaluation of single classifier model and ensemble model based on PCA-80 data of compound Caco 2 permeability were carried out. The modeling data were calculated by EBPT, RF, SVM, KNN, PLS-DA algorithm separately or combined with GA optimization operation for several times, respectively. The best classification model was screened with the prediction accuracy as the index. The constructed models were named Caco 2-EBPT, Caco 2-RF, Caco 2-SVM, Caco 2-KNN, Caco 2-PLS-DA, Caco 2-EBPT+GA, Caco 2-RF+GA, Caco 2-SVM+GA, Caco 2-KNN+GA and Caco 2-PLS-DA+GA, respectively. As far as EBPT algorithm is concerned, the Caco 2-EBPT model has better performance. After undergoing GA optimization, the prediction accuracy, Precision, Recall, F-measure and AUC values of the model have not been significantly improved. The quality and accuracy of the prediction model established by the other four algorithms combined with GA have been improved than their single classifier model. They had the better search capability and higher degree of convergence for discriminating the Caco 2 permeability of a compound. Detailed data are shown in Table S8 and Table S4.

Table S8 Statistic evaluations, ER, Precision, Recall, F-measure, AUC and OA evaluated by 4 single classifiers alone or combined with GA in the test set of Caco 2

| Model | ER (%) | Precision (%) | Recall (%) | F-measure (%) | AUC | OA (%) |
| --- | --- | --- | --- | --- | --- | --- |
| Caco 2-EBPT | 6.53 | 97.08 | 91.16 | 94.46 | 0.9659 | 93.47 |
| Caco 2-EBPT+GA | 12.07 | 83.45 | 79.85 | 83.87 | 0.9598 | 87.93 |
| Caco 2-SVM | 8.95 | 98.56 | 86.50 | 92.13 | 0.9909 | 91.05 |
| Caco 2-SVM+GA | 4.19 | 96.37 | 96.76 | 96.57 | 0.9919 | 95.81 |
| Caco 2-RF | 7.29 | 97.78 | 90.53 | 94.02 | 0.9896 | 92.71 |
| Caco 2-RF+GA | 6.35 | 95.28 | 94.07 | 94.67 | 0.9895 | 93.65 |
| Caco 2-KNN | 13.20 | 99.47 | 74.40 | 85.13 | 0.9915 | 86.80 |
| Caco 2-KNN+GA | 13.07 | 96.43 | 81.12 | 88.11 | 0.9836 | 86.93 |

***Evaluation of the Caco 2 prediction models***

At first, the evaluation index set of the 10 prediction models had been formed, which consist of five factors and based on comprehensive analysis principles. Specifically, it is carried out for TRS, TES and VAS three data sets respectively by calculating the values of MCC, SE, SP, OA and ER. The static parameters evaluated by the derived models were listed in Table S9. It realizes that the classification ability of GA optimized models is better than that based on single classifier for the Caco 2 permeability properties of compounds in this paper. Compared with Caco 2-EBPT model and Caco 2-EBPT+GA model, other models have good prediction performance for Caco 2 permeability property discrimination, and their prediction accuracy for TRS and VAS data sets are above 86.80%. Except for Caco 2-KNN model (73.66%), the prediction accuracy of other 7 models for TES data set is more than 87.37%. In a word, except for SVM, the predictive performance of each single classifier is inferior to that established by the corresponding model combined with GA, suggesting that GA is benefit to improve the ability of those Caco 2 permeability forecasting models in this research.

***AD analysis of the Caco 2 prediction models***

AD analysis was conducted on the QSAR models of Caco 2 permeability obtained in this study, and the Williams plots are shown in Figure S5. The *h** values of 5 single classifiers models of compounds Caco 2 permeability are all 0.0994. The *h** values of model EBPT+GA, SVM+GA, PLS-DA+GA, RF+GA and KNN+GA were 0.0653, 0.0455, 0.0170, 0.0739 and 0.0994, respectively. As characterized in Figure S5G ~ S5H, for Caco 2-RF, Caco 2-RF+GA, Caco 2-KNN and Caco 2-KNN+GA models, the SR values of compounds in TRS and VAS are all in a scope of –6 ~ 6, indicating that there is no response outlier. As characterized in Figure S5G ~ S5H, For Caco 2-KNN and Caco 2-KNN+GA models, the SR values of compounds in TRS and VAS are within a region of −3 ~ 3 (Figure S5I ~ S5J), respectively, implying that there is no Y outlier. The above results exhibit that samples of the two data sets (TRS and VAS) of KNN, GA+KNN, RF and RF+GA all fallen within the effective range of AD. For the EBPT, SVM, PLS-DA, EBPT+GA, SVM+GA, PLS-DA+GA models of Caco 2, the SR values of samples in two datasets are in an area of –8 ~ 8, –6 ~ 6 and –7 ~ 7, respectively, and there is no Y outlier. The *h* values for only a few individual compounds in TRS and VAS of the 6 models are less than the warning leverage value (*h*^*^), infers that most of the samples in TRS and VAS of the 6 models fall within the appropriate AD. Except for about 7 X exceptional points in Caco 2-PLS-DA+GA model, there are only 1 X exception point in TRS and VAS of the other five models. Those X outliers are not far beyond the critical value and are within the dotted line range, indicating that they are not statistical outliers. To sum up, the results show that the developed 10 Caco 2 permeability prediction models covers diverse compounds, and are reliable and robustness.

Table S9 Prediction performance evaluation of each model of Caco 2 permeability

| Model | Data Set | OA (%) | SP (%) | SE (%) | MCC | ER (%) |
| --- | --- | --- | --- | --- | --- | --- |
| Caco 2-PLS-DA | TRS | 86.84 | 100.00 | 78.21 | 0.7661 | 13.16 |
|  | TES | 87.37 | 100.00 | 78.38 | 0.7753 | 12.63 |
|  | VAS | 89.95 | 100.00 | 83.48 | 0.8150 | 10.05 |
| Caco 2-PLS-DA+GA | TRS | 99.43 | 100.00 | 99.09 | 0.9880 | 0.57 |
|  | TES | 98.98 | 100.00 | 98.33 | 0.9790 | 1.02 |
|  | VAS | 99.49 | 100.00 | 99.17 | 0.9893 | 0.51 |
| Caco 2-SVM | TRS | 96.69 | 100.00 | 94.57 | 0.9336 | 3.31 |
|  | TES | 95.48 | 97.37 | 94.31 | 0.9066 | 4.52 |
|  | VAS | 96.48 | 97.22 | 96.06 | 0.9250 | 3.52 |
| Caco 2-SVM+GA | TRS | 99.43 | 100.00 | 99.07 | 0.9881 | 0.57 |
|  | TES | 94.68 | 91.43 | 96.61 | 0.8858 | 5.32 |
|  | VAS | 94.65 | 88.89 | 98.26 | 0.8874 | 5.35 |
| Caco 2-RF | TRS | 100.00 | 100.00 | 100.00 | 1.0000 | 0.00 |
|  | TES | 93.75 | 96.00 | 92.31 | 0.8724 | 6.25 |
|  | VAS | 91.67 | 96.97 | 88.89 | 0.8303 | 8.33 |
| Caco 2-RF+GA | TRS | 100.00 | 100.00 | 100.00 | 1.0000 | 0.00 |
|  | TES | 93.91 | 93.06 | 94.40 | 0.8696 | 6.09 |
|  | VAS | 93.40 | 93.02 | 93.69 | 0.8661 | 6.60 |
| Caco 2-EBPT | TRS | 52.55 | 0.00 | 58.80 | -0.2632 | 47.45 |
|  | TES | 70.75 | 57.89 | 77.94 | 0.3605 | 29.25 |
|  | VAS | 67.92 | 86.36 | 54.84 | 0.4188 | 32.08 |
| Caco 2-EBPT+GA | TRS | 32.74 | 0.00 | 33.95 | -0.2549 | 67.26 |
|  | TES | 83.96 | 66.67 | 95.31 | 0.6664 | 16.04 |
|  | VAS | 54.72 | 84.00 | 28.57 | 0.1500 | 45.28 |
| Caco 2-KNN | TRS | 86.80 | 17.65 | 97.69 | 0.2563 | 13.20 |
|  | TES | 73.66 | 0.00 | 94.16 | -0.1155 | 26.34 |
|  | VAS | 86.89 | 0.00 | 97.55 | -0.0524 | 13.11 |
| Caco 2-KNN+GA | TRS | 86.93 | 17.65 | 97.84 | 0.2627 | 13.07 |
|  | TES | 99.70 | 100.00 | 99.70 | 1.0000 | 0.30 |
|  | VAS | 100.00 | 100.00 | 100.00 | 1.0000 | 0.00 |


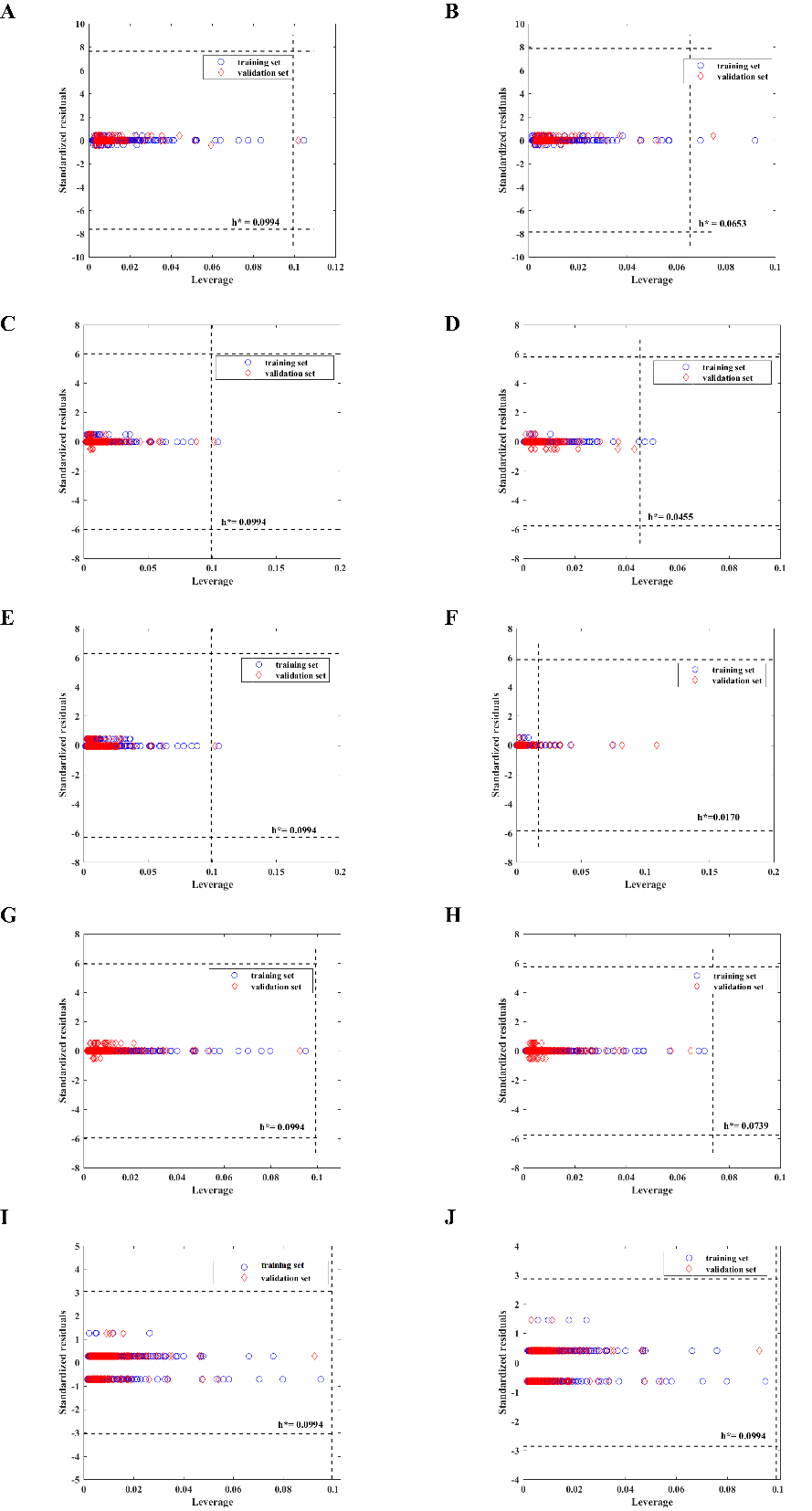


**Figure S5 Williams plots of standardized residuals versus leverage values (*h*) for the Caco 2 discrimination models.** (*h*^*^ is the warning leverage value.) A: Model Caco 2-EBPT, B: Model Caco 2-EBPT+GA, C: Model Caco 2-SVM, D: Model Caco 2-SVM+GA, E: Model Caco 2-PLS-DA, F: Model Caco 2-PLS-DA+GA, G: Model Caco 2-RF, H: Model Caco 2-RF+GA, I: Model Caco 2-KNN, J: Model Caco 2-KNN+GA.

**Establishment of HIA prediction model**

***HIA data distribution and feature selection***

PCA was used to investigate reduced-dimension and the rationality of data sets division of HIA characteristics of the HIA (+) and HIA (-) categories compounds. As shown as Figure S1D, a good trend of separation isn’t found among the three data sets of two categories, and there are some overlaps. The sample points of TRS can basically cover the whole range, indicating that the rationality of data set division in this study and the representativeness of sample points in TRS are relatively good. The variance contribution rate of each principal component was calculated, and the top ten principal components with higher variance contribution rate were drawn as shown in Figure S2D. The variance contribution rates of PC_1_, PC_2_, PC_3_ and PC_4_ decreased in turn, which are 28.38%, 9.69%, 6.84% and 5.50%, respectively. The variance contribution rates of other PCs are less than 5%. The cumulative variance contribution rate of the first 24 PCs is 80.55%, indicating that the first 24 PCs could reflect 80.55% of the original data in the experiment. The 24 significant latent PCs were selected to established the QSAR model of HIA property.

***Construction and optimization of HIA prediction models***

The modeling data of HIA were calculated by EBPT, RF, SVM, KNN, PLS-DA algorithm separately or combined with the optimization operation of GA for many times to comprehensively evaluate and select the best HIA property prediction model. Those models were recorded as HIA-EBPT, HIA-SVM, HIA-RF, HIA-KNN, HIA-PLS-DA, HIA-EBPT+GA, HIA-SVM+GA, HIA-RF+GA, HIA-KNN+GA and HIA-PLS-DA+GA respectively. OA, ER, AUC, Precision, Recall and F-measure were used to evaluate the impact of GA on the classification effectiveness of the QSAR model.

The OA of HIA-EBPT, HIA-SVM, HIA-RF and PGPI-PLS-DA models are 99.22%, 100.00%, 98.90% and 96.63%, respectively, which are higher than those of the corresponding models established by their combined application with GA. The results display that the HIA property prediction models established by these four single classifiers had better performance. The OA of HIA-KNN and HIA-KNN+GA models are 99.22% and 99.48%, respectively. The value of Precision and Recall of HIA-KNN model are 100.00% and 93.04%, which are higher than that of HIA-KNN+GA model. While F-measure and AUC values of HIA-KNN+GA model are 93.98% and 0.9931 respectively, which are greater than that of HIA-KNN model (88.11% and 0.9924). Although the little difference between HIA-KNN and HIA-KNN+GA, the results demonstrate that HIA-KNN+GA model is more effective on HIA property classification. For HIA, GA algorithm has no significant improvement on the performance of the five single classifiers used in this study. Detailed data are shown in Table S10 and Table S4.

Table S10 Statistic evaluations, ER, Precision, Recall, F-measure, AUC and OA evaluated by 4 single classifiers alone or combined with GA in the test set of HIA

| Model | ER (%) | Precision (%) | Recall (%) | F-measure (%) | AUC | OA (%) |
| --- | --- | --- | --- | --- | --- | --- |
| HIA-EBPT | 0.78 | 100.00 | 98.40 | 99.19 | 0.8862 | 99.22 |
| HIA-EBPT+GA | 6.43 | 94.36 | 94.49 | 94.32 | 0.9133 | 93.57 |
| HIA-SVM | 0.00 | 100.00 | 100.00 | 100.00 | 0.9952 | 100.00 |
| HIA-SVM+GA | 0.24 | 99.52 | 100.00 | 99.52 | 0.9803 | 99.76 |
| HIA-RF | 0.00 | 100.00 | 97.47 | 98.72 | 0.9747 | 98.80 |
| HIA-RF+GA | 1.69 | 99.05 | 97.66 | 98.35 | 0.9668 | 98.31 |
| HIA-KNN | 0.78 | 100.00 | 93.04 | 88.11 | 0.9924 | 99.22 |
| HIA-KNN+GA | 0.52 | 98.98 | 89.45 | 93.98 | 0.9931 | 99.48 |

***Evaluation of the HIA prediction model***

MCC, SE, SP, OA and ER were selected as indicators to evaluate the predictive ability of 10 models in the field of predicting the HIA property of compounds using TRS, TES and VAS data sets. The comparison results of performance evaluation indexes of each prediction model are shown in Table S11.

Compared with HIA-EBPT, HIA-EBPT+GA, HIA-KNN and HIA-KNN+GA models, the OA values of TRS, TES and VAS HIA-PLS-DA+GA, HIA-SVM, HIA-SVM+GA, HIA-RF and HIA-RF+GA models are all more than 94.69%. These results indicate that the prediction ability of these models for the HIA property of compounds in this study is almost the same, and these models can be used to classify future data objects. The forecast performance of HIA-KNN model is better than that of HIA-KNN+GA model, especially for TES and VAS data sets. To sum up, ten types of module achieved high accuracy on prediction and can be totally put into practice and can solve problem of HIA property prediction trouble of unknown compound. On the whole, compared with the ensemble models based on the features selected by GA, the prediction accuracy and the remaining four performance indicators (MCC, SE, SP and ER) of the models established by the single classifiers exhibit a better trend, but the difference is not significant.

***AD analysis of the HIA prediction models***

The application domains of the 10 prediction models for the HIA property of compounds were investigated. For the HIA-EBPT model and HIA-EBPT+GA model, as illustrated in Figure S6A ~ S6B, the standardized residual values of components in TRS and VAS are in a section of −8 ~ 8, indicating that there is no Y outlier. For the HIA-SVM, HIA-PLS-DA, HIA-RF, HIA-SVM, HIA-PLS-DA, HIA-RF and HIA-KNN model, as characterized by Figure S6C ~S6I, their SR values of samples in two datasets are in a range of −6 ~ 6, referring that the response outlier is also nonexistent in those models. The SR region of HIA-KNN+GA model is minimal interval, from −4 ~ 4, the Y outlier is not observed (Figure S6J).

The *h*^*^ values of HIA-EBPT, HIA-SVM, HIA-PLS-DA, HIA-RF and HIA-KNN models are all 0.0653, and the *h*^*^ values of HIA-PLS-DA+GA, HIA-SVM+GA, HIA-RF+GA, HIA-EBPT+GA and HIA-KNN+GA are 0.0575, 0.0392, 0.0444, 0.0392 and 0.0653, respectively. All the samples in TRS and VAS fell within the range of effective AD, which indicates that the 10 models established in this study are robustness and reliable for HIA prediction. They have good and effective internal forecasting ability.

Table S11 Prediction performance evaluation of each model of HIA

| Model | Data Set | OA (%) | SP (%) | SE (%) | MCC | ER (%) |
| --- | --- | --- | --- | --- | --- | --- |
| HIA-PLS-DA | TRS | 96.52 | 100.00 | 92.96 | 0.9325 | 3.48 |
|  | TES | 94.71 | 100.00 | 90.35 | 0.8994 | 5.29 |
|  | VAS | 96.14 | 100.00 | 92.52 | 0.9256 | 3.86 |
| HIA-PLS-DA+GA | TRS | 95.99 | 100.00 | 91.67 | 0.9225 | 4.01 |
|  | TES | 94.69 | 100.00 | 89.66 | 0.8991 | 5.31 |
|  | VAS | 94.69 | 100.00 | 89.83 | 0.8992 | 5.31 |
| HIA-SVM | TRS | 100.00 | 100.00 | 100.00 | 1.0000 | 0.00 |
|  | TES | 100.00 | 100.00 | 100.00 | 1.0000 | 0.00 |
|  | VAS | 100.00 | 100.00 | 100.00 | 1.0000 | 0.00 |
| HIA-SVM+GA | TRS | 99.91 | 100.00 | 99.82 | 0.9983 | 0.09 |
|  | TES | 99.01 | 98.92 | 99.09 | 0.9802 | 0.99 |
|  | VAS | 99.01 | 98.95 | 99.07 | 0.9802 | 0.99 |
| HIA-RF | TRS | 100.00 | 100.00 | 100.00 | 1.0000 | 0.00 |
|  | TES | 98.08 | 100.00 | 95.70 | 0.9617 | 1.92 |
|  | VAS | 99.52 | 100.00 | 99.05 | 0.9904 | 0.48 |
| HIA-RF+GA | TRS | 100.00 | 100.00 | 100.00 | 1.0000 | 0.00 |
|  | TES | 97.58 | 98.11 | 97.03 | 0.9517 | 2.42 |
|  | VAS | 99.03 | 100.00 | 98.23 | 0.9806 | 0.97 |
| HIA-EBPT | TRS | 40.31 | 0.00 | 41.29 | -0.1810 | 59.69 |
|  | TES | 86.96 | 86.76 | 87.23 | 0.7338 | 13.04 |
|  | VAS | 59.65 | 100.00 | 30.30 | 0.3934 | 40.35 |
| HIA-EBPT+GA | TRS | 70.19 | 0.00 | 85.71 | -0.1712 | 29.81 |
|  | TES | 92.17 | 96.49 | 87.93 | 0.8468 | 7.83 |
|  | VAS | 78.95 | 77.78 | 80.95 | 0.5709 | 21.05 |
| HIA-KNN | TRS | 97.82 | 100.00 | 95.64 | 0.9574 | 2.18 |
|  | TES | 100.00 | 100.00 | 100.00 | 1.0000 | 0.00 |
|  | VAS | 99.34 | 0.00 | 100.00 | 1.0000 | 0.66 |
| HIA-KNN+GA | TRS | 96.86 | 100.00 | 93.73 | 0.9391 | 3.14 |
|  | TES | 51.74 | 0.00 | 91.78 | -0.1939 | 48.26 |
|  | VAS | 50.97 | 0.00 | 91.67 | -0.1970 | 49.03 |


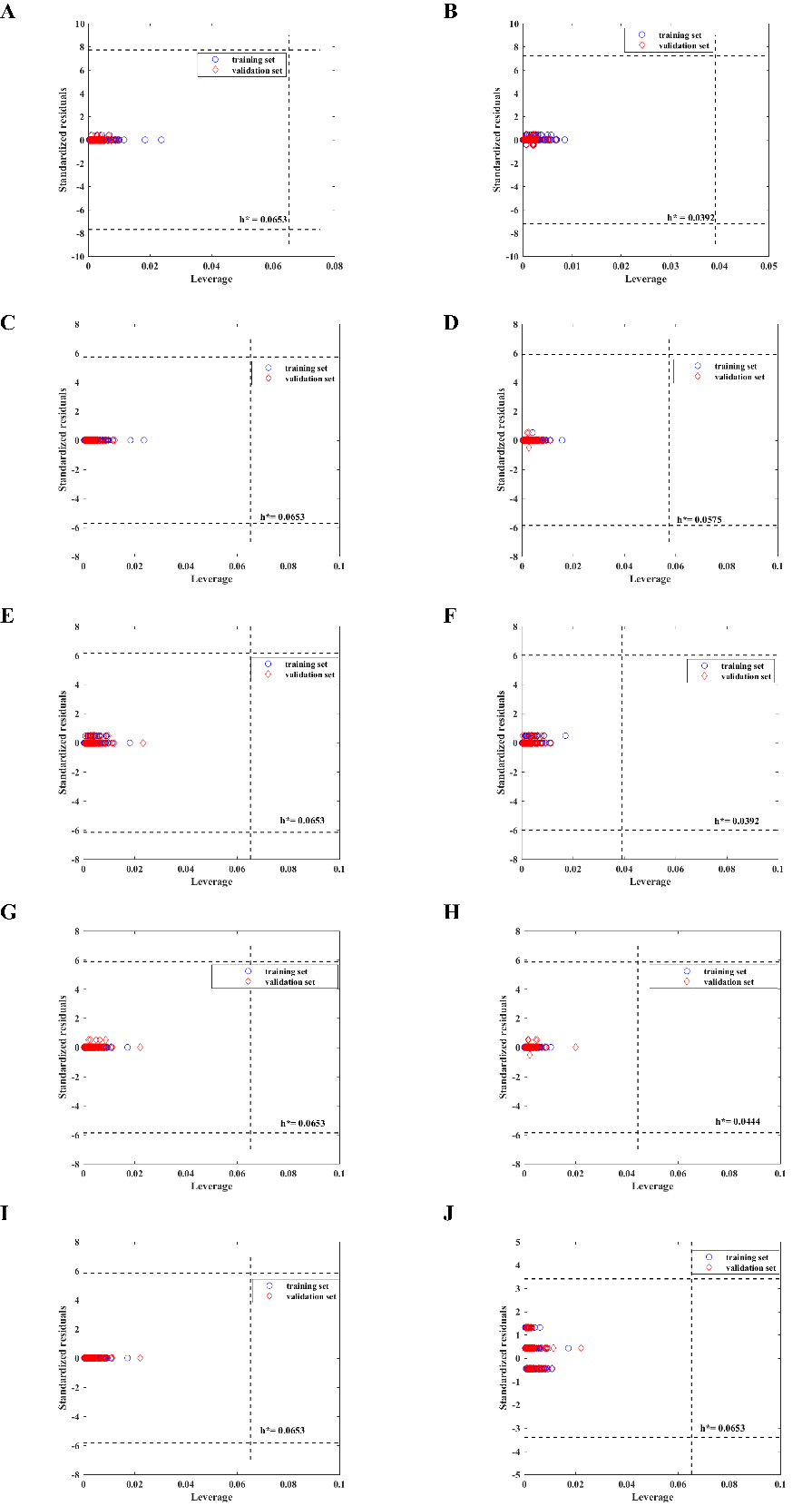


**Figure S6 Williams plots of standardized residuals versus leverage values (*h*) for the HIA discrimination models.** (*h*^*^ is the warning leverage value.) A: Model HIA-EBPT, B: Model HIA-EBPT+GA, C: Model HIA-SVM, D: Model HIA-SVM+GA, E: Model HIA-PLS-DA, F: Model HIA-PLS-DA+GA, G: Model HIA-RF, H: Model HIA-RF+GA, I: Model HIA-KNN, J: Model HIA-KNN+GA.

**Establishment of OB prediction models**

***OB data distribution and feature selection***

The results of PCA for the two kinds of compounds are shown in Figure S1E. The results reflect that although OB (+) and OB (-) tended to be separated, they are overlap to some extent. The variance contribution rates of PC_1_, PC_2_, PC_3_ and PC_4_ were 26.76%, 9.27%, 6.56% and 5.52%, respectively (Figure S2E). The variance contribution rates of the other PCs are less than 5%. The cumulative variance contribution rate of the first 27 PCs is 80.35%, pointing out that they could reflect 80.35% of the original data in the experiment. The 27 PCs were selected to progress the next analysis.

***Construction and optimization of OB prediction models***

The establishment and screening method of models were consistent with that of PGPI prediction model. Based on EBPT, KNN and GA algorithm, the QSAR models for the TRS dataset of OB attributes of compounds were respectively recorded as OB-EBPT, OB-EBPT+GA, OB-KNN and OB-KNN+GA. The OA values of the OB-EBPT and OB-KNN model are 76.35% and 72.33%, which are higher than that of their corresponding GA optimized models. For SVM, RF and PLS-DA, compared with their model obtained by the three individual classifiers, the values of Precision, Recall, F-measure, AUC and OA of the models optimized by GA are all basically improved. The statistic evaluations of OB-PLS-DA model and OB-PLS-DA+GA model are greater than that of the other 8 models. The results reckon that GA algorithm is helpful for the three individual classifiers to calculate a better OB property forecast model. Detailed data are shown in Table S12 and Table S4.

Table S12 Statistic evaluations, ER, Precision, Recall, F-measure, AUC and OA evaluated by 4 single classifiers alone or combined with GA in the test set of OB

| Model | ER (%) | Precision (%) | Recall (%) | F-measure (%) | AUC | OA (%) |
| --- | --- | --- | --- | --- | --- | --- |
| OB-EBPT | 13.28 | 70.54 | 80.63 | 79.68 | 0.8627 | 76.35 |
| OB-EBPT+GA | 32.34 | 52.97 | 86.03 | 77.93 | 0.8843 | 67.66 |
| OB-SVM | 29.35 | 75.14 | 73.89 | 74.51 | 0.9148 | 71.65 |
| OB-SVM+GA | 25.47 | 76.22 | 79.21 | 77.69 | 0.8699 | 74.53 |
| OB-RF | 29.97 | 70.92 | 75.96 | 73.35 | 0.9045 | 70.03 |
| OB-RF+GA | 28.12 | 71.92 | 82.49 | 76.84 | 0.9192 | 71.88 |
| OB-KNN | 29.21 | 70.79 | 73.33 | 72.13 | 0.8993 | 72.33 |
| OB-KNN+GA | 33.51 | 66.49 | 75.30 | 70.62 | 0.8873 | 72.10 |

***Evaluation of the OB prediction models***

In order to evaluate the forecast ability of 10 models for OB property prediction, three data sets (TRS, TES and VAS) were used to evaluate the predictive performance of the models with MCC, SE, SP, OA and ER as indicators. The results are shown in Table S13. As can be appreciated from Table S13, compared with other models, OB-PLS-DA+GA, OB-KNN and OB-KNN+GA have better prediction performance. These models' prediction accuracy for TRS, TES and VAS data sets are all above 72.10%. The seven models, OB-EBPT, OB-EBPT+GA, OB-PLS-DA, OB-SVM, OB-SVM+GA, OB-RF and OB-RF+GA, have poor prediction performance for the OB property of compounds, especially OB-EBPT. Similar to PGPI, the prediction performance and robustness of the models constructed by single classifier combined with GA, such as PLS-DA and EBPT, show a better trend for OB property, but the difference is not significant. The models established by the other three individual MLs are more reliable and robust compared with their correlated ensemble models.

Table S13 Prediction performance evaluation of each model of OB

| Model | Data Set | OA (%) | SP (%) | SE (%) | MCC | ER (%) |
| --- | --- | --- | --- | --- | --- | --- |
| OB-PLS-DA | TRS | 100.00 | 100.00 | 100.00 | 1.0000 | 0.00 |
|  | TES | 64.60 | 100.00 | 32.94 | 0.4339 | 35.40 |
|  | VAS | 68.32 | 100.00 | 45.74 | 0.5096 | 31.68 |
| OB-PLS-DA+GA | TRS | 94.14 | 100.00 | 89.13 | 0.8893 | 5.86 |
|  | TES | 91.76 | 100.00 | 85.42 | 0.8475 | 8.24 |
|  | VAS | 89.35 | 100.00 | 81.25 | 0.8073 | 10.65 |
| OB-SVM | TRS | 70.03 | 64.32 | 74.25 | 0.3862 | 29.97 |
|  | TES | 67.07 | 64.10 | 69.66 | 0.3379 | 32.93 |
|  | VAS | 75.90 | 61.33 | 87.91 | 0.5161 | 24.10 |
| OB-SVM+GA | TRS | 97.82 | 99.45 | 96.65 | 0.9560 | 2.18 |
|  | TES | 66.88 | 73.61 | 61.18 | 0.3483 | 33.12 |
|  | VAS | 60.90 | 68.00 | 54.32 | 0.2249 | 39.10 |
| OB-RF | TRS | 100.00 | 100.00 | 100.00 | 1.0000 | 0.00 |
|  | TES | 70.41 | 65.71 | 73.74 | 0.3930 | 29.59 |
|  | VAS | 69.64 | 60.71 | 78.57 | 0.3993 | 30.36 |
| OB-RF+GA | TRS | 100.00 | 100.00 | 100.00 | 1.0000 | 0.00 |
|  | TES | 68.79 | 47.62 | 82.98 | 0.3295 | 31.21 |
|  | VAS | 75.00 | 67.12 | 81.93 | 0.4976 | 25.00 |
| OB-EBPT | TRS | 28.54 | 0.00 | 30.60 | -0.3633 | 71.46 |
|  | TES | 62.07 | 83.78 | 46.00 | 0.3124 | 37.93 |
|  | VAS | 48.84 | 81.25 | 29.63 | 0.1205 | 51.16 |
| OB-EBPT+GA | TRS | 50.95 | 0.00 | 60.57 | -0.3061 | 49.05 |
|  | TES | 66.67 | 75.68 | 60.00 | 0.3547 | 33.33 |
|  | VAS | 72.09 | 72.73 | 71.43 | 0.4416 | 27.91 |
| OB-KNN | TRS | 72.33 | 61.20 | 81.11 | 0.4338 | 27.67 |
|  | TES | 100.00 | 100.00 | 100.00 | 1.0000 | 0.00 |
|  | VAS | 100.00 | 100.00 | 100.00 | 1.0000 | 0.00 |
| OB-KNN+GA | TRS | 72.10 | 61.46 | 80.49 | 0.4291 | 27.90 |
|  | TES | 100.00 | 100.00 | 100.00 | 1.0000 | 0.00 |
|  | VAS | 100.00 | 100.00 | 100.00 | 1.0000 | 0.00 |

***AD analysis of the OB prediction models***

After finishing all above work, AD analysis was carried out on ten different MLs (SVM, PLS-DA, KNN, EBPT, RF, GA+SVM, GA+PLS-DA, GA+RF, GA+KNN and GA+EBPT) of OB property. The Williams diagram are shown in Figure S7.

For the OB-EBPT, OB-PLS-DA and OB-PLS-DA+GA model, as characterized by Figure S7A, S7E and S7F, the SR values of samples in TRS and VAS are in a sphere of –7 ~ 7, indicating that there is no statistic outlier. For the OB-EBPT+GA, OB-SVM and OB-KNN+GA model, as represented by Figure S7B, S7C and S7J, the SR values of components in TRS and VAS are in a sphere of –5 ~ 5, displays that there is no Y outlier. For the OB-SVM+GA, OB-RF, OB-RF+GA and OB-KNN model, as illustrated by Figure S7D, S7G, S7H and S7I, the SR values of samples in TRS and VAS are in a sphere of –6 ~ 6, exhibits that there is no response outlier. The *h** of OB-EBPT, OB-PLS-DA, OB-SVM, OB-RF and OB-KNN models are all 0.0964. The *h** of model OB-EBPT+GA, OB-SVM+GA, OB-PLS-DA+GA, OB-RF+GA, OB-KNN+GA are 0.0344, 0.0482, 0.0310, 0.0620 and 0.0964, respectively. The data in TRS and VAS of model OB-EBPT, OB-PLS-DA, OB-SVM, OB-RF, OB-KNN, OB-EBPT+GA, OB-RF+GA, OB-KNN+GA are all within the valid range of AD. *h* values for a compound in the TRS and VAS of OB-PLS-DA+GA model is outside of the warning leverage value 0.0482 (Figure S7F), which displays that there is one X exception point. Evidenced by the same token, there are two X outliers in OB-SVM+GA model. These X exception points are close to the critical value and were within the dotted line range. The above results suggest that the developed 10 prediction models have good applicability and internal prediction, which covers diverse compounds.


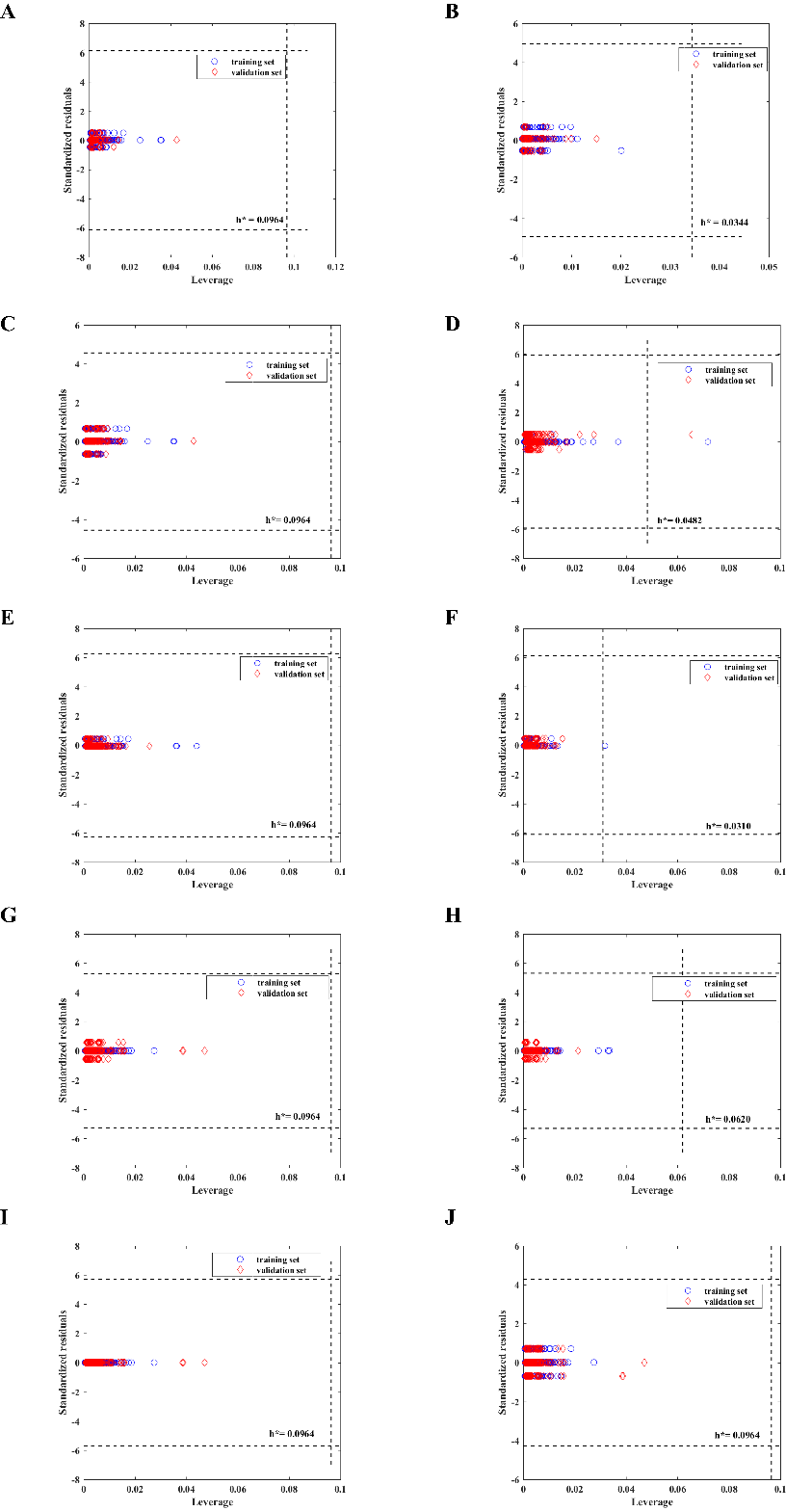


**Figure S7 Williams plots of standardized residuals versus leverage values (*h*) for the OB discrimination models.** (*h*^*^ is the warning leverage value.) A: Model OB-EBPT, B: Model OB-EBPT+GA, C: Model OB-SVM, D: Model OB-SVM+GA, E: Model OB-PLS-DA, F: Model OB-PLS-DA+GA, G: Model OB-RF, H: Model OB-RF+GA, I: Model OB-KNN, J: Model OB-KNN+GA.

Table S14 Prediction results of ADME properties chemical constituents in ARF

| Components | Caco 2 Permeability | P-gp substrate | P-gp inhibitor | OB | HIA |
| --- | --- | --- | --- | --- | --- |
| *p*-coumaric acid | +^[35]^ | -^[36]^ | - | +^[37]^ | +^[37]^ |
| catechol-*β*-D-glucopyranoside / arbutin | + | + | - | + | +^[38]^ |
| vanillic acid | - | + | - | + | +^[39]^ |
| citric acid | + | - | - | + | + |
| 3,4,5-trimethoxy-benzoic acid | + | - | - | + | + |
| gallic acid | +^[40]^ | -^[41]^ | - | + | + |
| protocatechuic acid | - | - | + | + | +^[39]^ |
| 9-octadecenic acid | + | - | - | + | + |
| (+)-catechin | +^[42]^ | -^[43]^ | - | +^[44]^ | + |
| 4-*O*-*p*-coumaroyl quinic acid | + | - | - | + | + |
| neochlorogenic acid | +^[45]^ | - | + | + | +^[46]^ |
| 4-hydroxy-2,6-dimethoxyphenol-1-*O*-*β*-D-glucopyranoside | + | + | - | + | + |
| quercetin-3-*O*-rutose-7-*O*-glucoside | +^[47]^ | - | + | + | + |
| MSTG-B | + | - | + | - | + |
| methyl salicylate lactoside/methyl salicylate gentiobioside | + | + | + | - | + |
| roseoside | + | + | + | - | + |
| MSTG-A | + | + | + | - | + |
| gaultherin | + | - | - | + | + |
| chlorogenic acid | + | - | + | +^[48, 49]^ | + |
| (-)-catechin | + | - | - | + | + |
| 5-*O*-*p*-coumaroyl quinic acid | - | - | + | + | + |
| (+)-lyoniresinol | + | - | - | + | + |
| (+)-homoeriodictyol | + | - | - | + | + |
| (-)-isolariciresinol-2-*α*-*O*-*β*-D-xylopyranoside | + | + | + | - | + |
| methyl salicylate vicianoside | + | + | - | - | + |
| methyl salicylate-*β*-glucoside | + | + | - | + | + |
| (+)-lyoniresinol-2-*α*-*O*-*β*-D-glucopyranoside | + | - | - | - | + |
| hesperetin | +^[50]^ | - | +^[51]^ | +^[52, 53]^ | +^[54]^ |
| paeoniflorin | +^[55]^ | +^[56]^ | - | -^[57-59]^ | +^[60]^ |
| (-)-5'-methoxyisolariciresinol-2*α*-*O*-*β*-D-xylopyranoside | + | + | + | - | + |

Table S14 Prediction results of ADME properties chemical constituents in ARF (Continued)

| Components | Caco 2 Permeability | P-gp substrate | P-gp inhibitor | OB | HIA |
| --- | --- | --- | --- | --- | --- |
| (+)-lyoniresinol-2-*α*-*O*-*β*-L-arabinopyranoside | + | + | + | - | + |
| myricetin | + | - | +^[61]^ | + | +^[62]^ |
| 2-hydroxy-4-methoxyacetophenone | + | + | - | + | + |
| quercetin | +^[63]^ | -^[64]^ | +^[65]^ | +^[63]^ | +^[62]^ |
| hyperoside | +^[66]^ | -^[67]^ | - | -^[68-70]^ | + |
| fraxinellonone | - | + | + | + | + |
| quercetin-3-*O*-glucuronide | + | - | - | - | + |
| avicularin | + | - | + | + | + |
| kaempferol-3-*O*-*β*-D-glucuronide | + | - | + | + | + |
| quercitrin | +^[47]^ | -^[71]^ | - | -^[72, 73]^ | +^[74]^ |
| 4-hydroxybenzoic acid | + | - | - | + | + |
| gaultherin A | + | - | + | + | + |
| Hexanal / 3-hexen-1-ol | + | - | - | + | + |
| kaempferol | +^[135]^ | -^[103]^ | +^[136]^ | + | +^[137]^ |
| gaultherin C | + | + | - | + | + |
| elemicine | + | + | + | +^[78]^ | + |
| bornyl acetate / geranyl acetate | - | -^[79]^ | -^[79]^ | - | + |

Note: "+", represents the large category of each property; "-", represents the small category of each property.

Table S17 KEGG Pathway information of ARF core targets for the treatment of RA

| Pathway ID | Pathway description | Count | Percentage (%) | Genes | *P*-value | Fold enrichment | Benjamini | FDR |
| --- | --- | --- | --- | --- | --- | --- | --- | --- |
| hsa05142 | Chagas disease (American trypanosomiasis) | 4 | 40 | ACE, MAPK14, NOS2, IL2 | 2.64E-04 | 2.65E+01 | 2.35E-02 | 2.87E-01 |
| hsa05205 | Proteoglycans in cancer | 4 | 40 | EGFR, MAPK14, MMP9, KDR | 1.79E-03 | 1.38E+01 | 7.73E-02 | 1.93E+00 |
| hsa04015 | Rap1 signaling pathway | 4 | 40 | EGFR, MAPK14, KIT, KDR | 2.06E-03 | 1.31E+01 | 5.99E-02 | 2.21E+00 |
| hsa05120 | Epithelial cell signaling in Helicobacter pylori infection | 3 | 30 | EGFR, MAPK14, ADAM17 | 3.22E-03 | 3.08E+01 | 7.00E-02 | 3.45E+00 |
| hsa04151 | PI3K-Akt signaling pathway | 4 | 40 | EGFR, KIT, KDR, IL2 | 8.38E-03 | 7.98E+00 | 1.41E-01 | 8.75E+00 |
| hsa05200 | Pathways in cancer | 4 | 40 | EGFR, MMP9, KIT, NOS2 | 1.20E-02 | 7.00E+00 | 1.66E-01 | 1.23E+01 |
| hsa04014 | Ras signaling pathway | 3 | 30 | EGFR, KIT, KDR | 3.32E-02 | 9.13E+00 | 3.52E-01 | 3.08E+01 |
| hsa05219 | Bladder cancer | 2 | 20 | EGFR, MMP9 | 5.24E-02 | 3.36E+01 | 4.54E-01 | 4.43E+01 |
| hsa04370 | VEGF signaling pathway | 2 | 20 | MAPK14, KDR | 7.71E-02 | 2.26E+01 | 5.52E-01 | 5.82E+01 |
| hsa05230 | Central carbon metabolism in cancer | 2 | 20 | EGFR, KIT | 8.07E-02 | 2.15E+01 | 5.31E-01 | 6.00E+01 |
| hsa05140 | Leishmaniasis | 2 | 20 | MAPK14, NOS2 | 8.92E-02 | 1.94E+01 | 5.34E-01 | 6.38E+01 |

Table S17 KEGG Pathway information of ARF core targets for the treatment of RA (Continued)

| Pathway ID | Pathway description | Count | Percentage (%) | Genes | *P*-value | Fold enrichment | Benjamini | FDR |
| --- | --- | --- | --- | --- | --- | --- | --- | --- |
| hsa05133 | Pertussis | 2 | 20 | MAPK14, NOS2 | 9.40E-02 | 1.83E+01 | 5.23E-01 | 6.58E+01 |
| hsa05132 | Salmonella infection | 2 | 20 | MAPK14, NOS2 | 1.04E-01 | 1.66E+01 | 5.31E-01 | 6.96E+01 |
| hsa04912 | GnRH signaling pathway | 2 | 20 | EGFR, MAPK14 | 1.13E-01 | 1.51E+01 | 5.37E-01 | 7.29E+01 |
| hsa04066 | HIF-1 signaling pathway | 2 | 20 | EGFR, NOS2 | 1.19E-01 | 1.43E+01 | 5.32E-01 | 7.48E+01 |
| hsa04915 | Estrogen signaling pathway | 2 | 20 | EGFR, MMP9 | 1.22E-01 | 1.39E+01 | 5.20E-01 | 7.58E+01 |
| hsa04660 | T cell receptor signaling pathway | 2 | 20 | MAPK14, IL2 | 1.24E-01 | 1.38E+01 | 5.02E-01 | 7.62E+01 |
| hsa04668 | TNF signaling pathway | 2 | 20 | MAPK14, MMP9 | 1.32E-01 | 1.29E+01 | 5.06E-01 | 7.85E+01 |
| hsa05145 | Toxoplasmosis | 2 | 20 | MAPK14, NOS2 | 1.35E-01 | 1.25E+01 | 4.97E-01 | 7.94E+01 |
| hsa04670 | Leukocyte transendothelial migration | 2 | 20 | MAPK14, MMP9 | 1.41E-01 | 1.20E+01 | 4.95E-01 | 8.08E+01 |
| hsa05160 | Hepatitis C | 2 | 20 | EGFR, MAPK14 | 1.61E-01 | 1.03E+01 | 5.29E-01 | 8.52E+01 |
| hsa04068 | FoxO signaling pathway | 2 | 20 | EGFR, MAPK14 | 1.62E-01 | 1.03E+01 | 5.16E-01 | 8.54E+01 |

Table S17 KEGG Pathway information of ARF core targets for the treatment of RA (Continued)

| Pathway ID | Pathway description | Count | Percentage (%) | Genes | *P*-value | Fold enrichment | Benjamini | FDR |
| --- | --- | --- | --- | --- | --- | --- | --- | --- |
| hsa05152 | Tuberculosis | 2 | 20 | MAPK14, NOS2 | 2.09E-01 | 7.77E+00 | 6.01E-01 | 9.22E+01 |
| hsa04020 | Calcium signaling pathway | 2 | 20 | EGFR, NOS2 | 2.11E-01 | 7.69E+00 | 5.89E-01 | 9.24E+01 |
| hsa04510 | Focal adhesion | 2 | 20 | EGFR, KDR | 2.40E-01 | 6.68E+00 | 6.27E-01 | 9.49E+01 |
| hsa04010 | MAPK signaling pathway | 2 | 20 | EGFR, MAPK14 | 2.86E-01 | 5.44E+00 | 6.89E-01 | 9.75E+01 |
| hsa05206 | MicroRNAs in cancer | 2 | 20 | EGFR, MMP9 | 3.18E-01 | 4.81E+00 | 7.20E-01 | 9.84E+01 |

Table S18 GO item information of ARF core target for the treatment of RA

| Category | Term | Description | Count | Percentage (%) | Genes | *P-*Value | Fold Enrichment | Benjamini | FDR |
| --- | --- | --- | --- | --- | --- | --- | --- | --- | --- |
| Molecular Function | GO:0004716 | receptor signaling protein tyrosine kinase activity | 3 | 30 | EGFR, KIT, KDR | 1.13E-05 | 5.06E+02 | 8.96E-04 | 1.20E-02 |
| Biological Process | GO:0001934 | positive regulation of protein phosphorylation | 4 | 40 | EGFR, MMP9, ADAM17, KDR | 3.43E-05 | 5.29E+01 | 1.23E-02 | 4.71E-02 |
| Biological Process | GO:0008284 | positive regulation of cell proliferation | 5 | 50 | EGFR, ADAM17, KIT, KDR, IL2 | 6.60E-05 | 1.80E+01 | 1.18E-02 | 9.07E-02 |
| Biological Process | GO:0030335 | positive regulation of cell migration | 4 | 40 | EGFR, ADAM17, KIT, KDR | 1.04E-04 | 3.65E+01 | 1.24E-02 | 1.42E-01 |
| Molecular Function | GO:0004714 | transmembrane receptor protein tyrosine kinase activity | 3 | 30 | EGFR, KIT, KDR | 1.76E-04 | 1.33E+02 | 6.92E-03 | 1.86E-01 |
| Cellular Component | GO:0005615 | extracellular space | 6 | 60 | EGFR, ACE, MMP9, AKR1B1, KIT, IL2 | 2.14E-04 | 8.12E+00 | 1.17E-02 | 2.11E-01 |
| Molecular Function | GO:0008237 | metallopeptidase activity | 3 | 30 | ACE, MMP9, ADAM17 | 8.01E-04 | 6.25E+01 | 2.09E-02 | 8.46E-01 |
| Biological Process | GO:0030307 | positive regulation of cell growth | 3 | 30 | EGFR, ADAM17, IL2 | 8.70E-04 | 6.00E+01 | 7.54E-02 | 1.19E+00 |
| Biological Process | GO:0007169 | transmembrane receptor protein tyrosine kinase signaling pathway | 3 | 30 | EGFR, KIT, KDR | 1.13E-03 | 5.25E+01 | 7.85E-02 | 1.55E+00 |
| Molecular Function | GO:0005178 | integrin binding | 3 | 30 | EGFR, ADAM17, KDR | 1.34E-03 | 4.82E+01 | 2.62E-02 | 1.41E+00 |

Table S18 GO item information of ARF core target for the treatment of RA (Continued)

| Category | Term | Description | Count | Percentage (%) | Genes | *P-*Value | Fold Enrichment | Benjamini | FDR |
| --- | --- | --- | --- | --- | --- | --- | --- | --- | --- |
| Biological Process | GO:0043066 | negative regulation of apoptotic process | 4 | 40 | EGFR, MMP9, KDR, IL2 | 1.47E-03 | 1.48E+01 | 8.45E-02 | 2.00E+00 |
| Molecular Function | GO:0005088 | Ras guanyl-nucleotide exchange factor activity | 3 | 30 | EGFR, KIT, IL2 | 1.61E-03 | 4.40E+01 | 2.51E-02 | 1.69E+00 |
| Molecular Function | GO:0004713 | protein tyrosine kinase activity | 3 | 30 | EGFR, KIT, KDR | 2.14E-03 | 3.81E+01 | 2.78E-02 | 2.25E+00 |
| Biological Process | GO:1900015 | regulation of cytokine production involved in inflammatory response | 2 | 20 | MAPK14, NOS2 | 2.14E-03 | 8.40E+02 | 1.04E-01 | 2.90E+00 |
| Biological Process | GO:0018108 | peptidyl-tyrosine phosphorylation | 3 | 30 | EGFR, KIT, KDR | 2.85E-03 | 3.29E+01 | 1.20E-01 | 3.84E+00 |
| Biological Process | GO:0046777 | protein autophosphorylation | 3 | 30 | EGFR, KIT, KDR | 3.58E-03 | 2.93E+01 | 1.34E-01 | 4.81E+00 |
| Biological Process | GO:0031659 | positive regulation of cyclin-dependent protein serine/threonine kinase activity involved in G1/S transition of mitotic cell cycle | 2 | 20 | EGFR, ADAM17 | 4.28E-03 | 4.20E+02 | 1.43E-01 | 5.72E+00 |
| Cellular Component | GO:0045121 | membrane raft | 3 | 30 | EGFR, ADAM17, KDR | 4.34E-03 | 2.65E+01 | 1.13E-01 | 4.19E+00 |
| Cellular Component | GO:0005768 | endosome | 3 | 30 | EGFR, ACE, KDR | 5.16E-03 | 2.43E+01 | 9.05E-02 | 4.96E+00 |
| Biological Process | GO:0002446 | neutrophil mediated immunity | 2 | 20 | ACE, ADAM17 | 5.88E-03 | 3.05E+02 | 1.76E-01 | 7.78E+00 |
| Biological Process | GO:0000165 | MAPK cascade | 3 | 30 | EGFR, KIT, IL2 | 8.12E-03 | 1.92E+01 | 2.17E-01 | 1.06E+01 |
| Biological Process | GO:0035162 | embryonic hemopoiesis | 2 | 20 | KIT, KDR | 8.54E-03 | 2.10E+02 | 2.12E-01 | 1.11E+01 |

Table S18 GO item information of ARF core target for the treatment of RA (Continued)

| Category | Term | Description | Count | Percentage (%) | Genes | *P-*Value | Fold Enrichment | Benjamini | FDR |
| --- | --- | --- | --- | --- | --- | --- | --- | --- | --- |
| Biological Process | GO:0042523 | positive regulation of tyrosine phosphorylation of Stat5 protein | 2 | 20 | KIT, IL2 | 9.08E-03 | 1.98E+02 | 2.09E-01 | 1.18E+01 |
| Cellular Component | GO:0042629 | mast cell granule | 2 | 20 | AKR1B1, KIT | 1.03E-02 | 1.74E+02 | 1.33E-01 | 9.70E+00 |
| Biological Process | GO:0046427 | positive regulation of JAK-STAT cascade | 2 | 20 | AKR1B1, KIT | 1.17E-02 | 1.53E+02 | 2.47E-01 | 1.50E+01 |
| Biological Process | GO:0035924 | cellular response to vascular endothelial growth factor stimulus | 2 | 20 | MAPK14, KDR | 1.23E-02 | 1.46E+02 | 2.42E-01 | 1.56E+01 |
| Biological Process | GO:0045909 | positive regulation of vasodilation | 2 | 20 | EGFR, NOS2 | 1.54E-02 | 1.16E+02 | 2.81E-01 | 1.92E+01 |
| Biological Process | GO:0030217 | T cell differentiation | 2 | 20 | KIT, IL2 | 1.60E-02 | 1.12E+02 | 2.75E-01 | 1.98E+01 |
| Biological Process | GO:0042177 | negative regulation of protein catabolic process | 2 | 20 | EGFR, NOS2 | 1.70E-02 | 1.05E+02 | 2.78E-01 | 2.10E+01 |
| Molecular Function | GO:0004175 | endopeptidase activity | 2 | 20 | ACE, MMP9 | 2.84E-02 | 6.25E+01 | 2.78E-01 | 2.64E+01 |
| Biological Process | GO:0007173 | epidermal growth factor receptor signaling pathway | 2 | 20 | EGFR, ADAM17 | 2.96E-02 | 6.00E+01 | 4.18E-01 | 3.38E+01 |
| Biological Process | GO:0043406 | positive regulation of MAP kinase activity | 2 | 20 | EGFR, KIT | 3.12E-02 | 5.69E+01 | 4.19E-01 | 3.53E+01 |
| Biological Process | GO:0048661 | positive regulation of smooth muscle cell proliferation | 2 | 20 | EGFR, AKR1B1 | 3.17E-02 | 5.60E+01 | 4.10E-01 | 3.58E+01 |
| Biological Process | GO:0006950 | response to stress | 2 | 20 | EGFR, AKR1B1 | 3.22E-02 | 5.51E+01 | 4.01E-01 | 3.62E+01 |

Table S18 GO item information of ARF core target for the treatment of RA (Continued)

| Category | Term | Description | Count | Percentage (%) | Genes | *P-*Value | Fold Enrichment | Benjamini | FDR |
| --- | --- | --- | --- | --- | --- | --- | --- | --- | --- |
| Molecular Function | GO:0046934 | phosphatidylinositol-4,5-bisphosphate 3-kinase activity | 2 | 20 | EGFR, KIT | 3.26E-02 | 5.45E+01 | 2.79E-01 | 2.96E+01 |
| Molecular Function | GO:0019903 | protein phosphatase binding | 2 | 20 | EGFR, MAPK14 | 3.31E-02 | 5.36E+01 | 2.56E-01 | 3.00E+01 |
| Biological Process | GO:0014068 | positive regulation of phosphatidylinositol 3-kinase signaling | 2 | 20 | KIT, KDR | 3.43E-02 | 5.17E+01 | 4.08E-01 | 3.81E+01 |
| Biological Process | GO:0043547 | positive regulation of GTPase activity | 3 | 30 | EGFR, KIT, IL2 | 3.48E-02 | 8.92E+00 | 3.99E-01 | 3.85E+01 |
| Cellular Component | GO:0048471 | perinuclear region of cytoplasm | 3 | 30 | EGFR, AKR1B1, NOS2 | 3.56E-02 | 8.80E+00 | 3.29E-01 | 3.00E+01 |
| Biological Process | GO:0035690 | cellular response to drug | 2 | 20 | EGFR, NOS2 | 3.64E-02 | 4.87E+01 | 4.01E-01 | 3.99E+01 |
| Biological Process | GO:0048010 | vascular endothelial growth factor receptor signaling pathway | 2 | 20 | MAPK14, KDR | 3.79E-02 | 4.66E+01 | 4.03E-01 | 4.12E+01 |
| Biological Process | GO:0050729 | positive regulation of inflammatory response | 2 | 20 | EGFR, IL2 | 3.85E-02 | 4.60E+01 | 3.96E-01 | 4.16E+01 |
| Cellular Component | GO:0005576 | extracellular region | 4 | 40 | ACE, MMP9, KDR, IL2 | 3.85E-02 | 4.53E+00 | 3.03E-01 | 3.21E+01 |
| Molecular Function | GO:0005524 | ATP binding | 4 | 40 | EGFR, MAPK14, KIT, KDR | 3.88E-02 | 4.52E+00 | 2.68E-01 | 3.43E+01 |
| Biological Process | GO:0007623 | circadian rhythm | 2 | 20 | EGFR, NOS2 | 3.95E-02 | 4.48E+01 | 3.94E-01 | 4.25E+01 |

Table S18 GO item information of ARF core target for the treatment of RA (Continued)

| Category | Term | Description | Count | Percentage (%) | Genes | *P-*Value | Fold Enrichment | Benjamini | FDR |
| --- | --- | --- | --- | --- | --- | --- | --- | --- | --- |
| Biological Process | GO:0014066 | regulation of phosphatidylinositol 3-kinase signaling | 2 | 20 | EGFR, KIT | 4.10E-02 | 4.31E+01 | 3.95E-01 | 4.38E+01 |
| Biological Process | GO:0001503 | ossification | 2 | 20 | EGFR, MMP9 | 4.21E-02 | 4.20E+01 | 3.93E-01 | 4.46E+01 |
| Biological Process | GO:0043410 | positive regulation of MAPK cascade | 2 | 20 | KIT, KDR | 4.26E-02 | 4.15E+01 | 3.87E-01 | 4.50E+01 |
| Biological Process | GO:0046854 | phosphatidylinositol phosphorylation | 2 | 20 | EGFR, KIT | 4.93E-02 | 3.57E+01 | 4.24E-01 | 5.00E+01 |
| Biological Process | GO:0048015 | phosphatidylinositol-mediated signaling | 2 | 20 | EGFR, KIT | 5.54E-02 | 3.17E+01 | 4.53E-01 | 5.43E+01 |
| Biological Process | GO:0000187 | activation of MAPK activity | 2 | 20 | MAPK14, KIT | 5.59E-02 | 3.14E+01 | 4.47E-01 | 5.46E+01 |
| Molecular Function | GO:0004222 | metalloendopeptidase activity | 2 | 20 | MMP9, ADAM17 | 5.87E-02 | 2.99E+01 | 3.52E-01 | 4.73E+01 |
| Biological Process | GO:0071222 | cellular response to lipopolysaccharide | 2 | 20 | MAPK14, NOS2 | 5.90E-02 | 2.97E+01 | 4.55E-01 | 5.66E+01 |
| Biological Process | GO:0010629 | negative regulation of gene expression | 2 | 20 | ACE, NOS2 | 7.11E-02 | 2.45E+01 | 5.12E-01 | 6.37E+01 |
| Biological Process | GO:0008360 | regulation of cell shape | 2 | 20 | KIT, KDR | 7.26E-02 | 2.40E+01 | 5.10E-01 | 6.45E+01 |
| Cellular Component | GO:0005911 | cell-cell junction | 2 | 20 | ADAM17, KIT | 8.18E-02 | 2.12E+01 | 4.89E-01 | 5.68E+01 |
| Cellular Component | GO:0016020 | membrane | 4 | 40 | EGFR, ACE, ADAM17, KIT | 8.44E-02 | 3.31E+00 | 4.55E-01 | 5.80E+01 |
| Molecular Function | GO:0046872 | metal ion binding | 4 | 40 | ACE, ADAM17, KIT, NOS2 | 8.76E-02 | 3.26E+00 | 4.53E-01 | 6.22E+01 |

Table S18 GO item information of ARF core target for the treatment of RA (Continued)

| Category | Term | Description | Count | Percentage (%) | Genes | *P-*Value | Fold Enrichment | Benjamini | FDR |
| --- | --- | --- | --- | --- | --- | --- | --- | --- | --- |
| Biological Process | GO:0001666 | response to hypoxia | 2 | 20 | ADAM17, NOS2 | 8.85E-02 | 1.95E+01 | 5.75E-01 | 7.20E+01 |
| Biological Process | GO:0070374 | positive regulation of ERK1 and ERK2 cascade | 2 | 20 | EGFR, KDR | 9.00E-02 | 1.92E+01 | 5.72E-01 | 7.26E+01 |
| Biological Process | GO:0045944 | positive regulation of transcription from RNA polymerase II promoter | 3 | 30 | EGFR, MAPK14, IL2 | 9.34E-02 | 5.14E+00 | 5.77E-01 | 7.40E+01 |
| Biological Process | GO:0042127 | regulation of cell proliferation | 2 | 20 | KIT, NOS2 | 9.49E-02 | 1.82E+01 | 5.75E-01 | 7.46E+01 |
| Molecular Function | GO:0005516 | calmodulin binding | 2 | 20 | EGFR, NOS2 | 9.64E-02 | 1.79E+01 | 4.60E-01 | 6.59E+01 |

Note: Benjamini score was derived from the DAVID database. Percentage values were calculated from the associated genes/total gene.

**Supplementary references**

1. Wang Y.H., In silico modeling of ADME﹣P-glycoprotein and cytochrome P450 3A4 eazyme. Dalian, China: Dalian Institute of Chemical Physics. The Chinese Academy of Sciences; 2006.
2. Yuan H.B., Predict the ADME/T properties of drugs using supporting vector mechine based method. Chengdu, China: Sichuan University; 2007.
3. Zhuang X.M., Study on early and rapid prediction technology of drug metabolic properties (ADME) and evaluation on a series of compounds. Beijing, China: Academy of Military Medical Sciences; 2006.
4. Cianchetta G., Singleton R.W., Zhang M., et al. A pharmacophore hypothesis for P-glycoprotein substrate recognition using GRIND-based 3D-QSAR. J. Med. Chem. 2005; 48: 2927‐2935.
5. Dong J., Wang N.N., Yao Z.J., et al. ADMETlab: a platform for systematic ADMET evaluation based on a comprehensively collected ADMET database. J. Cheminform. 2018; 10: 29.
6. Palmeira A., Sousa E., Vasconcelos M.H., et al. Three decades of P-gp inhibitors: skimming through several generations and scaffolds. Curr. Med. Chem. 2012; 19: 1946‐2025.
7. Broccatelli F., Carosati E., Neri A., et al. A novel approach for predicting P-Glycoprotein (ABCB1) inhibition using molecular interaction fields. J. Med. Chem. 2011. 54: 1740‐1751.
8. Li Z., Zhuang X.M., Li S.Y., et al. Research progress of traditional Chinese medicine inhibitors of P-glycoprotein. Pharm. J. Chin. PLA. 2009; 25: 326-329.
9. Zhang Y.J., Establishment of P-glycoprotein inhibitor screening model and its application to compounds evaluation. Shanghai, China: East China Normal University; 2016.
10. Pham-The H., González-Álvarez I., Bermejo M., et al. The use of rule-based and QSPR approaches in ADME profiling: a case study on Caco-2 permeability. Mol. Inform. 2013; 32: 459‐479.
11. Zhu C., Jiang L., Chen T.M., et al. A comparative study of artificial membrane permeability assay for high throughput profiling of drug absorption potential. Eur. J. Med. Chem. 2002; 37: 399‐407.
12. Yazdanian M., Glynn S.L., Wright J.L., et al. Correlating partitioning and caco-2 cell permeability of structurally diverse small molecular weight compounds. Pharm. Res. 1998; 15: 1490‐1494.
13. Hou T.J., Zhang W., Xia K., et al. ADME evaluation in drug discovery. 5. Correlation of Caco-2 permeation with simple molecular properties. J. Chem. Inf. Comput. Sci. 2004; 44: 1585‐1600.
14. Artursson P., Karlsson J., Correlation between oral drug absorption in humans and apparent drug permeability coefficients in human intestinal epithelial (Caco-2) cells. Biochem. Biophys. Res. Commun. 1991; 175: 880‐885.
15. Camenisch G., Alsenz J., van de Waterbeemd H., et al. Estimation of permeability by passive diffusion through Caco-2 cell monolayers using the drugs' lipophilicity and molecular weight. Eur. J. Pharm. Sci. 1998; 6: 317‐324.
16. Kapetanovic I.M., Muzzio M., Huang Z., et al. Pharmacokinetics, oral bioavailability, and metabolic profile of resveratrol and its dimethylether analog, pterostilbene, in rats. Cancer Chemother. Pharmacol. 2011; 68: 593-601.
17. Reinboth M., Wolffram S., Abraham G., et al. Oral bioavailability of quercetin from different quercetin glycosides in dogs. Br. J. Nutr. 2010; 104: 198-203.
18. Sawardecker E.N., Sales-Pardo M., Amaral L A., Detection of node group membership in networks with group overlap. Eur Phys J B. 2009; 67: 277-284(8).
19. Wang Z., Yang H., Wu Z., et al. *In Silico* prediction of blood-brain barrier permeability of compounds by machine learning and resampling methods. Chem. Med. Chem. 2018; 13: 2189‐2201.
20. Gunturi S.B., Ramamurthi N., A novel approach to generate robust classification models to predict developmental toxicity from imbalanced datasets. SAR QSAR Environ. Res. 2014; 25: 711‐727.
21. Yap C.W., PaDEL-descriptor: an open source software to calculate molecular descriptors and fingerprints. J. Comput. Chem. 2011; 32: 1466‐1474.
22. Przyby#ek M., Cysewski P., Distinguishing cocrystals from simple eutectic mixtures: phenolic acids as potential pharmaceutical coformers. Cryst. Growth Des. 2018; 18: 3524−3534.
23. Chen X.Y., Xie W.C., Yang Y., et al. Discovery of Dual FGFR4 and EGFR Inhibitors by Machine Learning and Biological Evaluation. J. Chem. Inf. Model. 2020; 60: 4640-4652.
24. Lei T.L., Theoretical prediction of drug toxicity based on machine learning approaches. Hangzhou, China: Zhejiang university; 2017.
25. Trevino V., Falciani F., GALGO: an R package for multivariate variable selection using genetic algorithms. Bioinformatics. 2006; 22: 1154-1156.
26. Hu Y.H., Tai C.T., Tsai C.F., et al. Improvement of Adequate Digoxin Dosage: An Application of Machine Learning Approach. J. Healthc. Eng. 2018; 2018: 3948245.
27. Lu Z.Q.J., The Elements of Statistical Learning: Data Mining, Inference, and Prediction. Berlin, Germany: Springer, 2008; 101-649.
28. Wen L., Li Q., Li W., et al. A QSAR Study Based on SVM for the Compound of Hydroxyl Benzoic Esters. Bioinorg. Chem. Appl. 2017; 2017: 4914272.
29. Tripaldi P., Pérez-González A., Rojas C., et al. Classification-based QSAR Models for the Prediction of the Bioactivity of ACE-inhibitor Peptides. Protein Pept. Lett. 2018; 25: 1015‐1023.
30. Sun X.Q., Comparative analysis between the brain disease and sleep-disorder disease syndrome in brain blood differentiation of symptoms and signs. Chin. Archiv. Tradit. Chin. Med. 2012; 30: 2163-2166.
31. Lei L., Wang X.Z., Zhang L., et al. QSAR study on toxicity of chemical components of Chinese materia medica and acute toxicity of rats. Chin. J. Inform. Tradit. Chin. Med. 2016; 23: 43-46.
32. Saavedra L.M., Romanelli G.P., Duchowicz P.R., Quantitative structure–activity relationship (QSAR) analysis of plant-derived compounds with larvicidal activity against Zika Aedes aegypti (Diptera: Culicidae) vector using freely available descriptors. Pest Manag. Sci. 2018; 74: 1608‐1615.
33. Chen S.W., Prediction of antifungal activity and human intestinal absorption of drugs by using support vector machine. Chongqing, China: Sichuan University; 2005.
34. Zhang R., Quantitative structure-property relationship models of drug release from pH-sensitive block copolymer micelles. Guangzhou, China: South China University of Technology; 2017.
35. Bowles S.L., Ntamo Y., Malherbe C.J., et al. Intestinal transport and absorption of bioactive phenolic compounds from a chemically characterized aqueous extract of Athrixia phylicoides[J]. J. Ethnopharmacol. 2017, 200: 45‐50.
36. Cheng F., Li W., Zhou Y., et al. admetSAR: a comprehensive source and free tool for assessment of chemical ADMET properties. J. Chem. Inf. Model. 2012; 52(11): 3099‐3105.
37. Cui Y., The study on variations of chemical components and antioxidant activity of red wine during decanting process. Shenyang, China: Sheyang Pharmaceutical Univercity; 2011.
38. Gracey M., Burke V., Thomas J.A., et al. Effect of microorganisms isolated from the upper gut of malnourished children on intestinal sugar absorption in vivo. Am J. Clin. Nutr. 1975; 28: 841‐845.
39. Liu C.F., Research on the change law of metabolites of cyaniding-3-glucoside in gastrointestinal tract of rats. Hanzhong, China: Shaanxi University of Technology; 2019.
40. Konishi Y., Kobayashi S., Shimizu M., Transepithelial transport of p-coumaric acid and gallic acid in Caco-2 cell monolayers. Biosci. Biotechnol. Biochem. 2003; 67: 2317‐2324.
41. Shaik M., Vanapatla S.R., Enhanced oral bioavailability of linagliptin by the influence of gallic acid and ellagic acid in male Wistar albino rats: involvement of p-glycoprotein inhibition. Drug Metab. Pers. Ther. 2019; 34: 20180020.
42. Song Q., Li D., Zhou Y., et al. Enhanced uptake and transport of (+)-catechin and (-)-epigallocatechin gallate in niosomal formulation by human intestinal Caco-2 cells. Int. J. Nanomed. 2014; 9: 2157‐2165.
43. Tan L.X., Study on the effect of Hordei Fructus Germinatus after “stir-frying” on ingredients content and intestinal absorption. Nanchang, China: Jiangxi University of Traditional Chinese Medicine; 2019.
44. Schneiderová K., Šmejkal K.. Phytochemical profile of *Paulownia tomentosa* (Thunb). Steud. Phytochem. Rev. 2015; 14: 799‐833.
45. Szwajgier D., Paduch R., Kukuła-Koch W., et al. Study on Biological Activity of Bread Enriched with Natural Polyphenols in Terms of Growth Inhibition of Tumor Intestine Cells. J. Med. Food. 2020; 23: 181‐190.
46. Wu P., Li M., Gong Z.P., et al. Analysis of intestinal absorption characteridtics of Inula cappa extract by in situ intestinal circulating perfusion model. Chin. J. Exp. Tradit. Med. Form. 2018; 24: 1-8.
47. Li S.Y., Study on absorption and metabolism of quercetin and its glycosides in Caco-2 cells. Beijing, China: Academy of Military Medical Sciences; 2010.
48. Sun B., Pharmacokinetic study of baicalin and chlorogenic in chicken after oral administion of Manhukang oral liquid. Haerbin, China: Northeast Agricultural University; 2013.
49. Jung J.W., Kim J.M., Jeong J.S., et al. Pharmacokinetics of chlorogenic acid and corydaline in DA-9701, a new botanical gastroprokinetic agent, in rats. Xenobiotica. 2014; 44: 635‐643.
50. Kobayashi S., Tanabe S., Sugiyama M., et al. Transepithelial transport of hesperetin and hesperidin in intestinal Caco-2 cell monolayers. Biochim. Biophys. Acta. 2008; 1778: 33–41.
51. Wen N., Zhang S.Q., Zhu L., et al. Recent progress in the investigation of P-glycoprotein. Chin. Pharm. Aff. 2011; 25: 718-723.
52. Shete G., Pawar Y.B., Thanki K., et al. Oral bioavailability and pharmacodynamic activity of hesperetin nanocrystals generated using a novel bottom-up technology. Mol. Pharm. 2015; 12: 1158‐1170.
53. Gu S.F., Wang L.Y., Tian Y.J., et al. Enhanced water solubility, antioxidant activity, and oral absorption of hesperetin by D-α-tocopheryl polyethylene glycol 1000 succinate and phosphatidylcholine. J. Zhejiang Univ. Sci. B. 2019; 20: 273‐281.
54. Chen R., Xin R., Chen L.H., et al. Study on rat intestinal absorption of naringenin and hesperetin in Situ. Chin J Mod App Pharm. 2013; 30: 465-469.
55. Li H., Cao X., Liu Y., et al. Establishment of modified biopharmaceutics classification system absorption model for oral Traditional Chinese Medicine (Sanye Tablet). J Ethnopharmacol. 2019; 244: 112148.
56. Shui W.B., He Q., Ge Z.W., et al. Studies on absorption of paeoniflorin in rat small intestines by HPLC-MS. Chin. Pharm. J. 2007;14: 1098-1101.
57. Ding G.C., Wang F.F., Ye X.L., Study on pharmacokinetics and bioavailability of paeoniflorin in rats. Northwest Pharm. J. 2009; 24: 124-125.
58. Chen Y.C., Qian J.H., Wang B.H., et al. Effect of different proportions of *Ramulus Cinnamomi* and *Radix Paeoniae Alba* on the pharmacokinetics of peaoniflorin in rats. Chin J Clin. Pharm. Ther. 2017; 22: 1237-1243.
59. Yu J.B., Zhao Z.X., Peng R., et al. Gut microbiota-based pharmacokinetics and the antidepressant mechanism of paeoniflorin. Front. Pharmacol. 2019; 10: 268.
60. Cai Y., Liang W.G., Liu X.Y., The study in situ on rat intestinal absorption kinetics of the active component paeoniflorin in extract of raidix paeoniae alba. China Med. Herald. 2011; 8: 39-42.
61. Lin G.B., Xie Y., Li G.W., Research advances of myricetin. J. Int. Pharm. Res. 2012; 39: 483-487.
62. Xue C.H., Study on absorption and metabolism of flavonoids from Abelmoschus manihot. Nanjing, China: Nanjing University of Chinese Medicine; 2011.
63. He Y., Study on absorption mechanism of quercetin and its derivatives in Caco-2 cells. Nanchang, China: Nanchang University; 2015.
64. Wang Y., Cao J., Zeng S., Establishment of a P-glycoprotein substrate screening model and its preliminary application. World J. Gastroenterol. 2004; 10: 1365‐1368.
65. Yu J., Chen H., Jiang L., et al. Codelivery of adriamycin and P-gp inhibitor quercetin using PEGylated liposomes to overcome cancer drug resistance. J. Pharm. Sci. 2019; 108: 1788‐1799.
66. Wang HL. Study on absorption and metabolism of flavonoids across human intestinal epithelial Caco-2 cells. Changchun, China: Jilin University; 2006.
67. Jia Y., Ma X.M., Yun F., et al. Apparent oil/water partition coefficients of hyperoside and its in situ intestinal absorption kinetics in rats. Chin. Tradit. Herb Drugs. 2012; 43: 934-939.
68. Chen S.S., Effects of different compatibility on the content and pharmacokinetics of hyperoside in *Cuscuta chinensis* Lam. Hefei, China: Anhui University of Chinese Medicine; 2019.
69. Li Z., Meng F., Zhang Y., et al. Simultaneous quantification of hyperin, reynoutrin and guaijaverin in mice plasma by LC-MS/MS: application to a pharmacokinetic study. Biomed. Chromatogr. 2016; 30: 1124‐1130.
70. Chen R., Li L., Shen C., et al. Intestinal transport of HDND-7, a novel hesperetin derivative, in *in vitro* MDCK cell and *in situ* single-pass intestinal perfusion models. Xenobiotica. 2017; 47: 719‐730.
71. Li S.Y., Li Z., Li J.L., et al. Comparison study on absorption characteristics of quercetin and its glycoside derivatives in Caco-2 monolayer cell model. Clinical nutrition branch of Chinese Nutrition Society. Data collection of the 13th National Clinical Nutrition Conference. Clinical nutrition branch of Chinese Nutrition Society: Chinese Nutrition Society; 2011: 155.
72. Zhou D.Q., The preliminary study of the quercetrin sodium chloride injection and pharmacokinetic study of quercetrin. Jinan, China: Shandong University of Traditional Chinese Medicine;2016.
73. Wei J., Zhang Y., Li D., et al. Integrating network pharmacology and component analysis study on anti-atherosclerotic mechanisms of total flavonoids of *Engelhardia roxburghiana Leaves* in Mice. Chem. Biodivers. 2020; 17: e1900629.
74. Hollman P.C., de Vries J.H., van Leeuwen S.D., et al. Absorption of dietary quercetin glycosides and quercetin in healthy ileostomy volunteers. Am. J. Clin. Nutr. 1995; 62: 1276–1282.
75. Tian X.J., Yang X.W., Yang X., et al. Studies of intestinal permeability of 36 flavonoids using Caco-2 cell monolayer model. Int. J. Pharm. 2009; 367: 58-64.
76. Wang Y., Cao J., Zeng S., Involvement of P-glycoprotein in regulating cellular levels of Ginkgo flavonols: quercetin, kaempferol, and isorhamnetin. J. Pharm. Pharmacol. 2005; 57: 751‐758.
77. Tian Y., Jiang X.H., Yu J., et al. Intestinal absorption of kaempferol in rats *in vivo*. J. Sichuan. Univ. (Med. Sci. Ed.). 2008; 03: 503-505.
78. Wang Z., Wang Q., Yang B., et al. GC-MS method for determination and pharmacokinetic study of four phenylpropanoids in rat plasma after oral administration of the essential oil of Acorus tatarinowii Schott rhizomes. J. Ethnopharmacol. 2014; 155: 1134–1140.
79. Yoshida N., Takagi A., Kitazawa H., et al. Inhibition of P-glycoprotein-mediated transport by extracts of and monoterpenoids contained in Zanthoxyli fructus. Toxicol. Appl. Pharmacol. 2005; 209: 167‐173.
